# Continuous diagnosis and prognosis by controlling the update process of deep neural networks

**DOI:** 10.1016/j.patter.2023.100687

**Published:** 2023-02-03

**Authors:** Chenxi Sun, Hongyan Li, Moxian Song, Derun Cai, Baofeng Zhang, Shenda Hong

**Affiliations:** 1 Key Laboratory of Machine Perception (Ministry of Education), Peking University, Beijing 100871, China; 2 School of Intelligence Science and Technology, Peking University, Beijing 100871, China; 3 National Institute of Health Data Science, Peking University, Beijing 100191, China; 4 Institute of Medical Technology, Health Science Center of Peking University, Beijing 100191, China

**Keywords:** diagnosis, prognosis, disease staging, biomarker, sepsis, COVID-19, deep learning, continuous classification, time series

## Abstract

Continuous diagnosis and prognosis are essential for critical patients. They can provide more opportunities for timely treatment and rational allocation. Although deep-learning techniques have demonstrated superiority in many medical tasks, they frequently forget, overfit, and produce results too late when performing continuous diagnosis and prognosis. In this work, we summarize the four requirements; propose a concept, continuous classification of time series (CCTS); and design a training method for deep learning, restricted update strategy (RU). The RU outperforms all baselines and achieves average accuracies of 90%, 97%, and 85% on continuous sepsis prognosis, COVID-19 mortality prediction, and eight disease classifications, respectively. The RU can also endow deep learning with interpretability, exploring disease mechanisms through staging and biomarker discovery. We find four sepsis stages, three COVID-19 stages, and their respective biomarkers. Further, our approach is data and model agnostic. It can be applied to other diseases and even in other fields.

## Highlights

• Restricted update strategy for training deep-learning models is presented
• Method can be used for continuous classification of time series
• Method is effective in continuous diagnosis and prognosis in the medical field
• Method can find disease stages and biomarkers

## The bigger picture

Patients in intensive care require rapid and accurate diagnoses at multiple time points. The quality and speed of these diagnoses can have a major impact on the success of treatment regimes for life-threatening conditions like sepsis or severe COVID-19. There is hope that deep-learning techniques could be used to create computer-based systems that would provide continuous real-time diagnoses, but these techniques face special challenges when dealing with time series data. Here, we develop and describe a method for training deep neural networks for these kinds of challenging applications and show that they can increase accuracy and provide better interpretability to the outputs, making them easier to apply in a clinical setting. Methods such as this one may ultimately help improve automated diagnosis systems in crucial clinical settings and could have applications in other time-sensitive settings that require continuous classification.

## Graphical abstract

**Figure:**
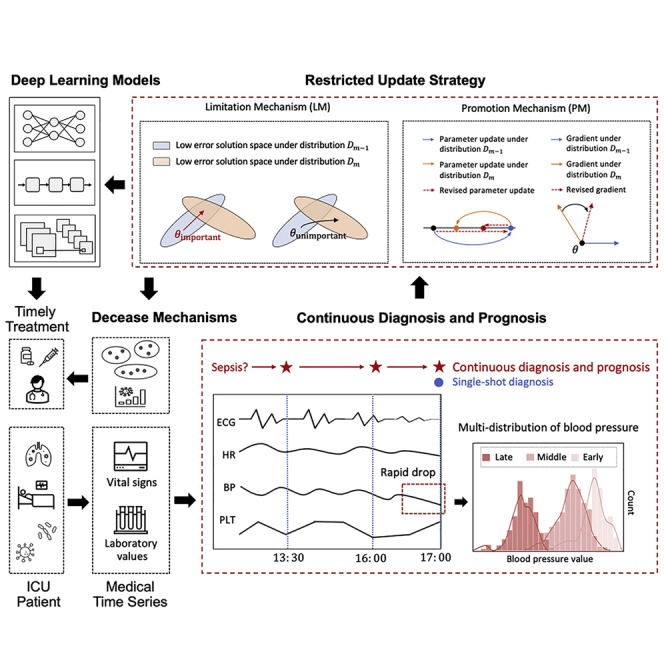

## Teaser

Continuous diagnosis and prognosis are crucial for patients in intensive care. They can provide more opportunities for timely treatment and rational resource allocation. Based on the task of continuous classification of time series, a restricted update strategy for deep neural networks is proposed. It can not only achieve continuous diagnosis and prognosis but can also detect stages and biomarkers of the disease, making the deep-learning model interpretable.

## Introduction

Continuous diagnosis and prognosis are of great significance for timely, personalized treatment and rational allocation of medical resources. Especially in the intensive care unit (ICU), status perception and disease diagnosis are needed at any time as real-time diagnosis provides more opportunities for doctors to rescue lives. For example, sepsis is a life-threatening condition, causing more than half of ICU deaths.^1^ Early detection and antibiotic treatment are critical for improving sepsis outcomes;^2^^,^^3^ COVID-19 outbreaks have caused health concerns worldwide. In the case of a sudden outbreak of a new epidemic, continuous prognosis can help with personalized treatment and rational allocation of scarce resources.^4^^,^^5^

**Figure 1:**
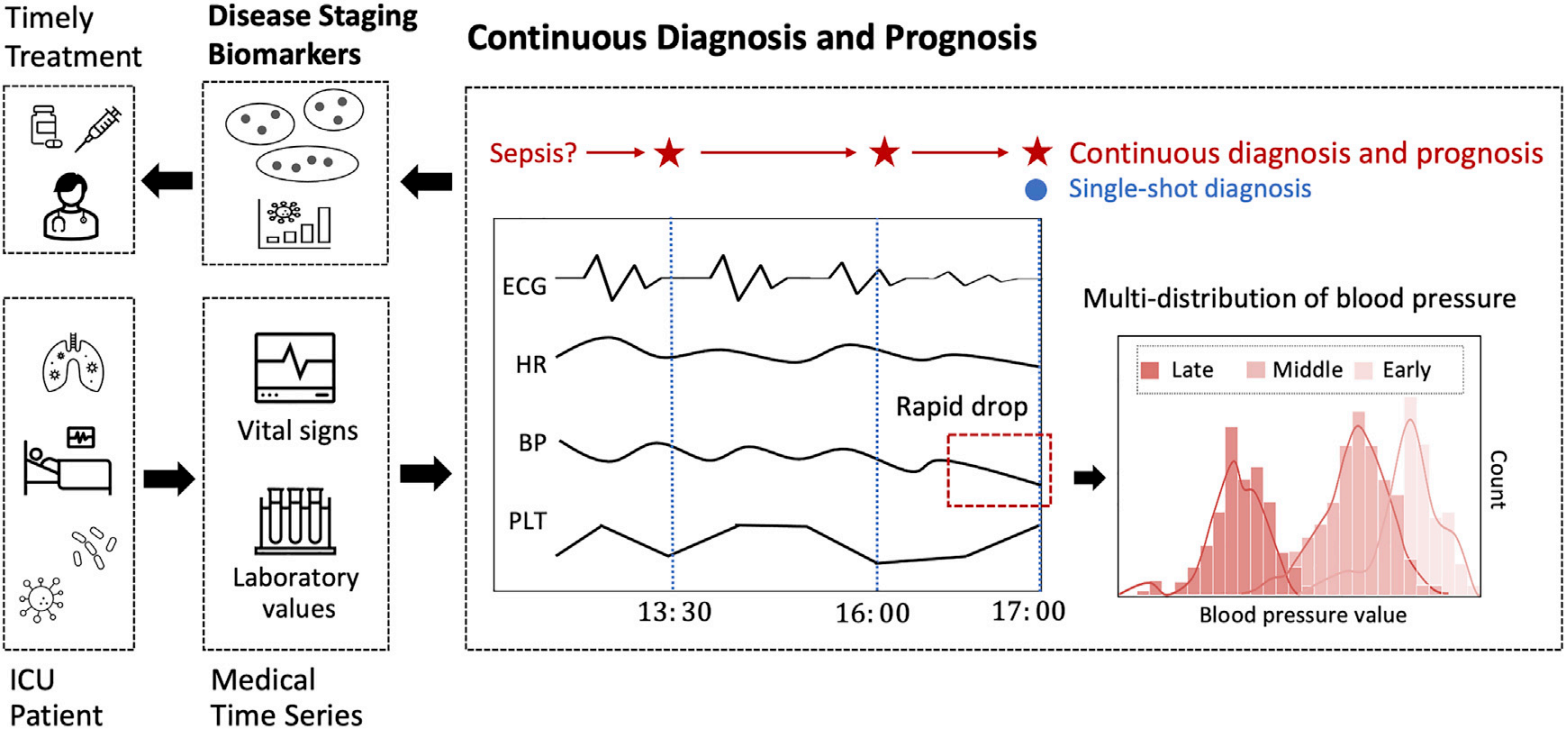
Continuous diagnosis and prognosis with disease staging

Different from the single-shot diagnosis, which is often made for the outpatient, the task of continuous diagnosis and prognosis emphasizes the multiple early diagnoses or prognoses for the inpatient at different stages over time. For example, in Figure 1, a patient in the ICU is monitored for vital signs in real time. Assuming that he will be in sepsis shock at 17:00, the common diagnostic system will give a warning when he is suffering or about to suffer from sepsis at about 17:00 (the single-shot diagnosis, blue dot). This is likely to miss the emergency treatment time for the acute disease, where each hour of delay has been associated with roughly a 4%–8% increase in sepsis mortality.^2^ Thus, we require a continuous prognosis mode for sepsis (red stars), where we can predict the patient outcome 4 h early, 1 h early, etc., at 13:00, 16:00, etc. To meet this practical need, we summarize four requirements for the task of continuous diagnosis and prognosis.

### Requirement 1: The ability to identify symptoms in different time stages before the disease onset

Single-shot diagnosis only needs learning the clinical manifestation, which is easy under the guidance of the gold standard. But continuous prognosis needs to learn the underlying symptoms of the disease in its early stages, which are usually not obvious in the clinic and cannot be judged by clinicians, and the symptoms are not only from a certain stage but from multiple stages before the onset, leading to diversity and hybridity.

### Requirement 2: Potential for earlier diagnosis with guaranteed accuracy

Earlier diagnosis is necessary for many severe illnesses. Each hour of delayed treatment could cause a 4%–8% increase in sepsis mortality,^2^ but basic questions about the limits of early detection remain unanswered. If one wants to pursue higher diagnostic accuracy, it will tend to predict late for clearer features. For example, a rapid drop in blood pressure (a major symptom of sepsis shock; the red dashed box in Figure 1) always occurs just before the shock.^1^ But we expect the continuous mode to diagnose earlier and more accurately than the single-shot mode.

### Requirement 3: Merits of explainability and disease staging

The 22nd article of the European Union’s General Data Protection Regulation stipulates that a subject of algorithmic decisions has a right to meaningful explanation regarding said decisions.^6^ As clinicians always justify a result using medical-domain knowledge familiar to them, the explainable methods will be more popular in practice.^7^

Meanwhile, continuous prognosis is accompanied by disease progression. Disease staging is important to understand disease mechanisms and implement targeted treatment. A clinically useful staging system stratifies patients by their baseline risk of an adverse outcome and their potential to respond to therapy. The best developed and most explicit approach has evolved in oncology,^8^ but it is not clear for critical illnesses. Recently, the stratification for sepsis (sepsis, severe sepsis, and septic shock) has been questioned in the latest sepsis definition,^1^ and there are no criteria for temporal septic stages.

### Requirement 4: Function of offline and sustainable use

In many scenarios, especially in the ICU, we need to directly use the mature system without constant adjustment. A well-informed system can reduce the risk of misjudgment.^9^ Further, in subsequent applications, when obtaining a batch of new data, such as new patients and new clinical observations, we hope to continue to use the current system instead of designing a new one because the data that have the old knowledge may still occur, while the new system cannot handle the old knowledge well.^10^

Nowadays, many studies have shown that deep learning (DL) methods are superior to medical gold standards and experienced doctors in some medical tasks such as outcome prediction and disease diagnosis.^11^^,^^12^ Surprisingly, in these studies, sequential medical records, such as vital signs, multiple blood samples, and serial medical imaging, provided more possibilities for DL models to implement diagnosis and prognosis. We uniformly name such sequential medical records as medical time series data. However, most DL-based models often give the single-shot diagnosis after learning the full-length medical time series but cannot prognose continuously. Although some subdisciplines study the mode of continuous learning, they cannot satisfy the above requirements at the same time (see supplemental related work and concepts).

The labels (mortality, morbidity, etc.) of real-world medical time series are usually determined at the final time. If the model simply learns the full-length time series, it can only give the single-shot result at the onset time. For continuous diagnosis and prognosis, the model needs to learn time series from different advanced stages: when the data change, the model performance needs to maintain. But most medical time series have evolved distribution. In Figure 1, the blood pressure varies among early, middle, and late stages, bringing a triple distribution. DL models lack the ability to learn all distributions simultaneously due to the premise of independent and identical distribution. As shown in Figure S4, learning the new knowledge may lead to forgetting old ones, and learning one distribution frequently may fall into local solutions with overfitting.

Meanwhile, interpretability is an elusive concept, and the artificial intelligence (AI) field holds no consensus regarding its definition.^13^ Although some studies have proposed methods to explain the DL black-box model,^14^ they are not fully interpretable, mostly explaining static models and depending on the actual scenario and task.^15^ Thus, interpretation problems have not yet been fully overcome when using DL for medical applications.^16^ When developing the method, we need to consider the possibility of it being explained and match it to the dynamic process of continuous diagnosis and prognosis.

To this end, we establish a training method for DL models, the restricted update strategy (RU) of neural network parameters. The RU can satisfy the above requirements: for requirement 1, it has the limitation mechanism (LM) to avoid catastrophic forgetting and overfitting; for requirement 2, it has the promotion mechanism (PM) to consolidate the knowledge of early distribution; for requirement 3, we define the importance coefficient of parameters to reveal the model development and achieve disease staging with typical biomarkers; and for requirement 4, we train the model by real-world datasets with separate training and test sets and test continual use. Experimental results show that the RU is more accurate than all baselines, achieving accuracies of 90%, 97%, and 85% on sepsis prognosis, COVID-19 mortality prediction, and eight disease diagnoses, respectively.

The major advantages of our study are 4-fold: (1) for continuous diagnosis and prognosis of time-sensitive illness, we design an RU for the DL model, which outperforms baselines. (2) The RU has the ability to interpret the update of the DL model and the change of medical time series through input indicators and parameter visualization. These side effects make our method attractive in medical applications where model interpretation and marker discovery are required. (3) We extend our method to connect the distribution change of vital signs with the parameter change of the DL model. We find typical disease biomarkers and stages of sepsis and COVID-19. (4) The RU is a data-agnostic, model-agnostic, and easy-to-use plug in. It can be used to train various types of DL models. The continuous prediction mode is needed in most time-sensitive applications, not just in medical tasks. As shown in Figure S2, we define this task as a distinctive concept—continuous classification of time series (CCTS), which is different from existing concepts and tasks as shown in Figure S3.

## Results

We test the RU and eight baselines on six datasets, using 5-fold cross-validation, expressed as the mean and SD (mean ± SD). The classification accuracy is evaluated by the area under the curve of the receiver operating characteristic (AUC-ROC; the higher the better) and the AUC confidence interval. The continuous classification performance is evaluated by backward transfer (BTW) and forward transfer (FWT; the higher the better). The statistical significance is evaluated by the Bonferroni-Dunn test. The learning stability is evaluated by the gradient fluctuation R (the lower the better). Eight baselines are long short-term memory (LSTM), stopping rule (SR),^17^ effective confidence-based early classification (ECEC),^18^ online stochastic recursive gradient-based Frank-Wolfe (ORGFW),^19^ gradient episodic memory (GEM),^20^ elastic weight consolidation (EWC),^21^ continual learning with experience and replay (CLEAR),^22^ and continual learning of physiological signals (CLOPS).^23^ Three medical datasets are SEPSIS,^24^ COVID-19,^25^ and MIMIC-III.^26^ Three additional datasets are United States Historical Climatology Network (USHCN),^27^ University of California Riverside time series classification archive (UCR),^28^ and human ACTIVity dataset (ACTIV)^29^ (more experimental details are in the supplemental experimental procedures).

### An RU to train DL models for continuous diagnosis and prognosis

For continuous medical diagnosis and prognosis, we focus on continuous sepsis prognosis, continuous COVID-19 mortality prediction, and continuous eight disease classification based on medical time series, including vital signs from various monitors, and continuous blood sample records during hospitalization. All used data are available: the SEPSIS dataset^24^ has 30,336 ICU patient records with 2,359 diagnosed with sepsis from three separate hospital systems; the COVID-19 dataset^25^ has 6,877 blood sample records of 485 COVID-19 patients from Tongji Hospital, Wuhan, China; and the MIMIC-III dataset^26^ has 19,993 admission records from 7,537 patients, and we focus on 8 diseases.

**Figure 2:**
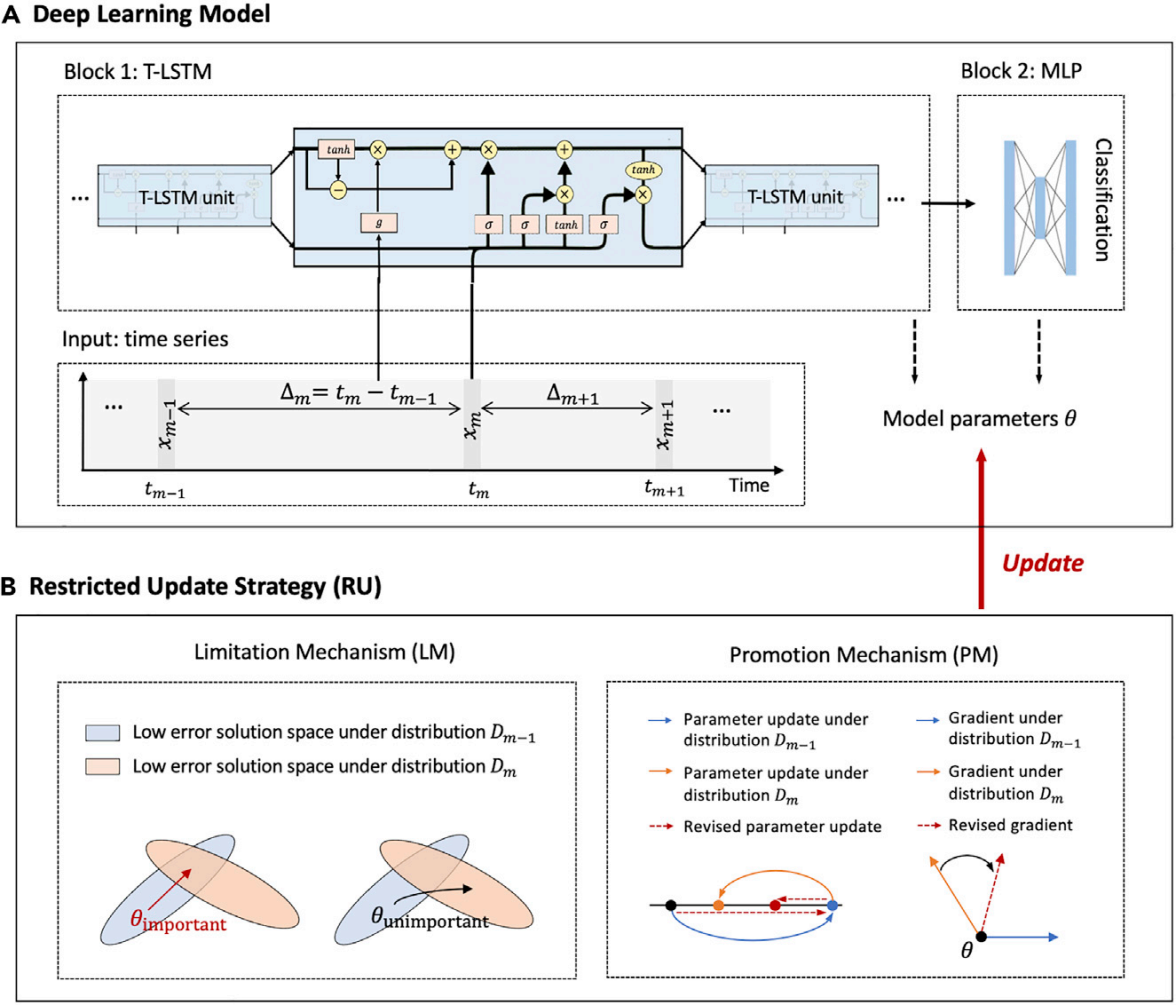
Restricted update strategy (RU) of neural networks for continuous classification of time series (CCTS)

A time series dataset $T={\left\{X^{n}\right\}}_{n=1}^{N}$ has N samples. Each sample $X={\left\{x_{m}\right\}}_{m=1}^{M}$ has M observations with value $x_{m}$ and time $t_{m}$. Multivariate time series can be described by $X={\left\{x_{m}^{d}\right\}}_{d,m=1}^{D,M}$. d is the d-th dimension. DL models have achieved great success in modeling medical time series data,^30^ especially recurrent neural networks (RNNs). However, the real-world time series is usually long and irregularly sampled. For example, critically ill patients are often hospitalized for several months; thus, records often have hundreds of observations. And due to the change in the patient’s health status, the relevant measurement requirements are also changing, which may be several hours or days apart.^31^ Thus, in order to model the long-term dependency and eliminate the impact of uneven time intervals, we implement time-aware LSTM (T-LSTM),^32^ a variant of the RNN. As shown in Figure 2A, our DL architecture has two blocks: block 1 uses T-LSTM to model the input data and represent their hidden features with the consideration of time decay $\Delta_{m}=t_{m}-t_{m-1}$, and block 2 uses multilayer perceptron (MLP) to map features to the class.

**Figure 3:**
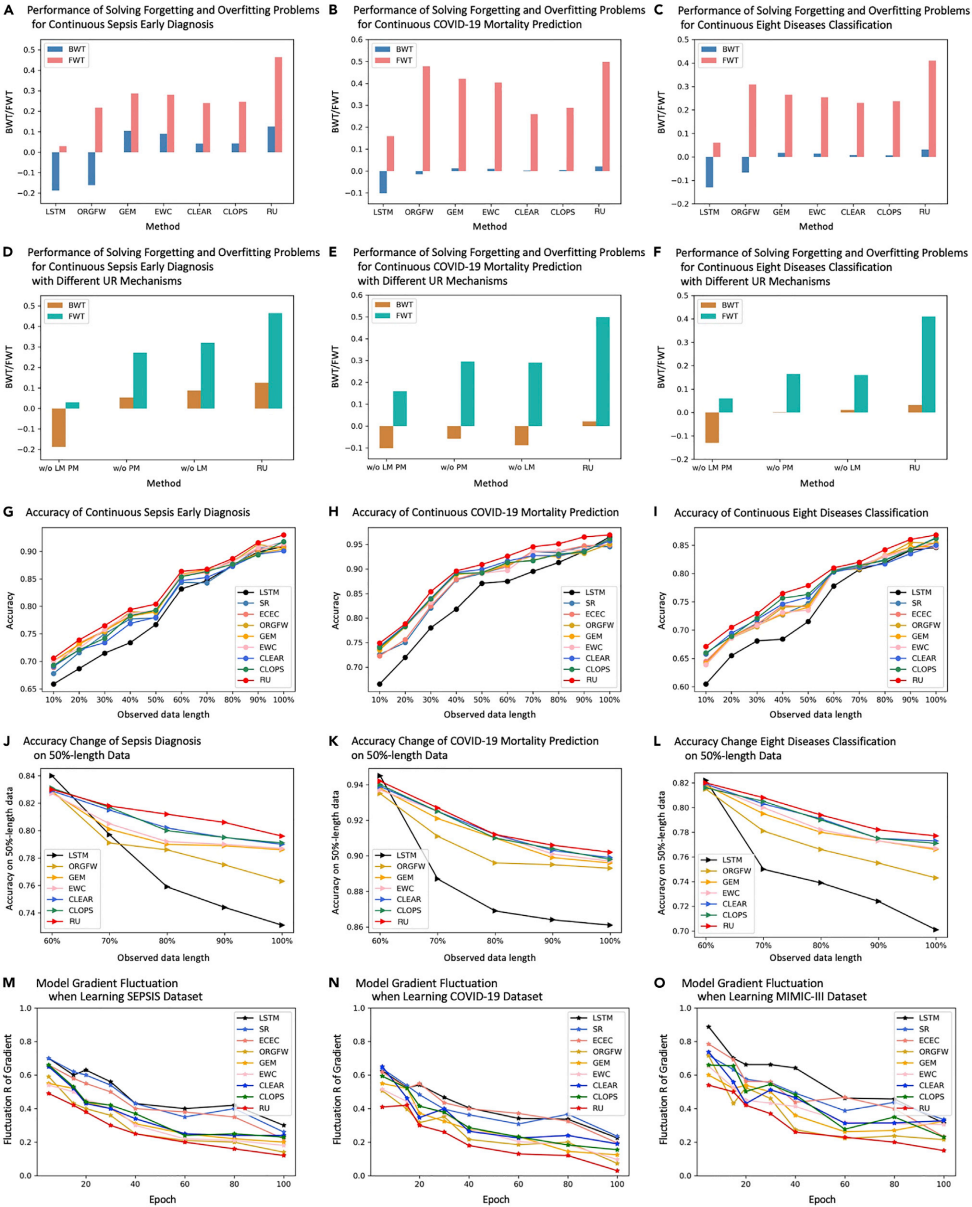
Method performances on continuous diagnosis and prognosis The continuous classification performance is evaluated by backward transfer (BWT ↑) and forward transfer (FWT ↑). The classification accuracy is evaluated by the area under the curve of the receiver operating characteristic (AUC-ROC ↑). The 95% average AUC-ROC confidence intervals of the RU on SEPSIS and COVID-19 are (0.8624,0.8776) and (0.9477,0.9723). The RU is significantly better (p<0.05) than LSTM (p=0.0116); SR^17^(p=0.0293); ECEC^18^(p=0.0321); and ORGFW^19^(p=0.0201) and is better than GEM^20^(p=0.0656); EWC^21^(p=0.0676); CLEAR^22^(p=0.0703); and CLOPS^23^(p=0.0527). And in the Bonferroni-Dunn test, $k=9,n=3,m=5,q_{0.05}=2.724,N=15,\bar{r}=3.53>CD=2.72$. Thus, the accuracy is significantly improved by the RU.

After training T-LSTM by the full-length time series dataset, it can achieve average accuracies of 92%, 97%, and 88% on single-shot diagnosis for sepsis, COVID-19, and eight diseases, respectively. However, when applying it to continuous diagnosis and prognosis, the accuracy drops by more than 15%. Thus, we use the dataset consisting of subsequences of each sample in the original dataset T to train the model. The new dataset is $T^{*}={\left\{X_{1:m}^{n}\right\}}_{n,m=1}^{N,M}$. As the time series changes dynamically, datasets $T_{m-1}^{*}$, $T_{m}^{*}$ in time $t_{m-1}$, $t_{m}$ may form different data distributions $D_{m-1}$, $D_{m}$. To learn such multidistribution, we propose an RU to train the DL model. As shown in Figure 2B, the RU has two mechanisms: LM and PM. The RU has the most stable gradient in the training process. As shown in Figures 3M–3O, it has the smallest R in any epoch, showing its restriction ability in the error back propagation of the DL model.

LM helps the DL model to learn multidistributed data, alleviating problems of catastrophic forgetting and overfitting. Due to the observation of many parameter configurations resulting in the same performance,^33^ we could add a regular term to the loss to restrict model parameters. To this end, when learning a new distribution, LM constrains important parameters $\theta_{\text{important}}$ for the old distribution to stay close to their old values but changes unimportant parameters ($\theta_{\text{unimportant}}$) more. As shown in Figure 2B, when learning distribution $D_{m}$, $\theta_{\text{important}}$ is limited to the low error space of distribution $D_{m-1}$, while $\theta_{\text{unimportant}}$ can be updated to other spaces. In this way, different parameters can be arranged for different distributions. The importance of parameter θ is measured by the importance coefficients α(θ). LM is implemented by using a loss in Equation 5. Its key feature is the use of the diagonal of the Fisher information matrix F to represent the importance coefficient α, allowing for the quantification of parameter importance.

PM helps the DL model to classify time series earlier in time-sensitive applications. It regards the process of a DL model learning early distributions and new distributions as the same continuous optimization problem with regret minimization. PM is projection free and estimates a stochastic recursive estimator to alleviate the complexity and training instability. As shown in Figure 2B, when learning distribution $D_{m}$, PM changes the current gradient from an obtuse angle to an acute angle with the gradient on previous distribution $D_{m-1}$ because when the new gradient and the old gradient are at an acute angle, the model performance on the old distribution will improve, or at least not decrease.^20^ Most importantly, the promotion of learning old distributions has the potential for early classification. PM is implemented by a recursive estimator in Equation 12. It can fill the gap between the optimal regret bound and the low per-round computational cost, holding a nearly optimal regret bound $\tilde{O}\left(\sqrt{M}\right)$, where M is the number of distributions.

Equations 5 and 12 serve as the main conduits for implementing the RU. It is clear that they have no bearing on the model’s structure or tasks. Thus, the RU is a model-agnostic, task-agnostic, and easy-to-use plug in.

### Finding 1: The continuous mode has more potential in medical diagnosis and prognosis than the single-shot mode

For the CCTS task, as shown in Figures 3G–3L, our method, RU, can classify more accurately at every time. It is significantly better than all 8 baselines in the Bonferroni-Dunn test $\left(\bar{r}=3.5>CD=2.724\right)$. The average accuracy is about 2% higher, especially in the early time, being 5% higher for 10%-length data. CCTS is important for time-sensitive applications, especially for acute and critical illnesses. Take sepsis diagnosis as an example: compared with the best baseline, the RU improves the accuracy by 1.4% on average and 2.2% in the early 50% time stage where the key features are unobvious. Each hour of delayed treatment increases mortality by 4%–8%.^2^ With the same accuracy, we can predict 0.972 h in advance.

The RU can alleviate catastrophic forgetting and overfitting when classifying time series continuously. Figure S3 shows that all three medical datasets have multiple distributions in the CCTS task. When learning multidistribution, as shown in Figures 3A–3C, the RU has the highest BWT and FWT, meaning it has the lowest negative influence that learning the new distribution has on old distributions and has the highest positive influence that learning the former data has on the overall task. Meanwhile, both LM and PM of the RU can contribute to the model performance. In Figures 3D–3F and S4 and Table S7, if we remove two mechanisms, respectively, the model performance will decline.

### Finding 2: The change of importance coefficients interprets the learning process of the DL model

When a DL model learns time series in different stages, its parameters are updated constantly. After using the RU, if the model encounters a new data distribution, the importance coefficient is likely to change significantly. Thus, we can explain the learning process of the DL model from the perspective of the change of importance coefficient.

**Figure 4:**
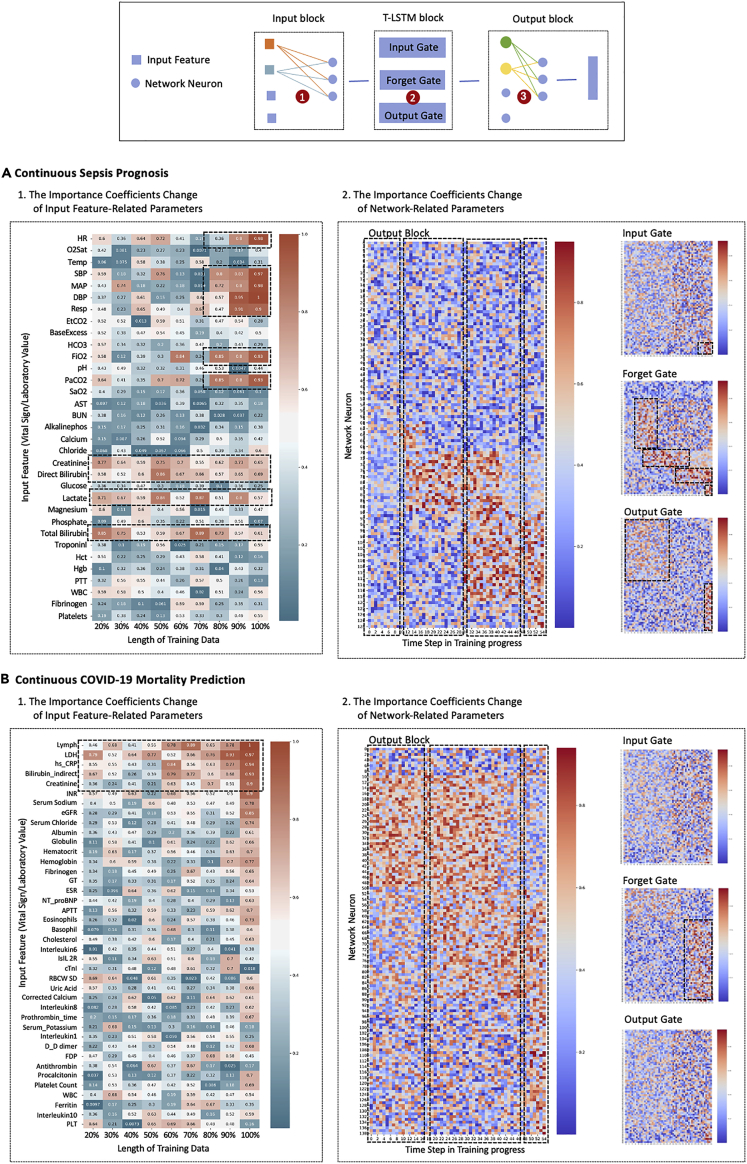
The importance of input features and network parameters

We divide the DL model into three blocks as shown in Figure 4. Block 1 is the input block. We focus on the parameter update process related to input features. For an input feature $x^{d}$ (d-th dimension sequence of input multivariate time series), we use the overall importance coefficient of its related parameters to measure its importance: $\alpha^{*}\left(x^{d}\right)=\sum_{n}\alpha \left(\theta_{x^{d},l_{1,n}}\right)$, where $\theta_{x^{d},l_{1,n}}$ is the weight between input feature $x^{d}$ and the n-th neuron in layer $l_{1}$. Block 2 is the T-LSTM block. We focus on the parameter update process related to different gates. For a gate $G_{i}$, we use the overall importance coefficient of its parameters to measure its importance: $\alpha^{*}\left(G_{i}\right)=\sum_{n}\alpha \left(\theta_{n}\right)$. Block 3 is the output block. We focus on the parameter update process related to network neurons. For the j-th neuron in layer $l_{i}$, we use the overall importance coefficient of its output weights to measure its importance: $\alpha^{*}\left(l_{i,j}\right)=\sum_{n}\alpha \left(\theta_{l_{i,j},l_{i+1,n}}\right)$. The test on three blocks can enhance the interpretability based on the input data and network structures.

When the model learns time series with different lengths (in different time stages), its perceptual sensitivity to input features is different. As shown in Figures 4A1 and 4B1, for sepsis diagnosis, the blood pressure’s $\alpha^{*}$ increases, which means that the model’s perception of blood pressure improved in the later stage. For COVID-19 mortality prediction, lymphocytes’ $\alpha^{*}$ is always high, which means that the model pays attention to this feature at all stages. Thus, $\alpha^{*}$ can be used to evaluate biomarkers.

We regard the importance coefficient of model parameters in the continuous learning process as a sequence and use the Bayesian online changepoint detection (BOCD) model^34^ to find change points in this sequence. N change points divide the sequence into n+1 stages. As shown in Figures 4A2 and 4B2, in the output block, the training process of the model can be roughly divided into four stages for sepsis diagnosis and three stages for COVID-19 mortality prediction. In each stage, the important parameters are different. In the T-LSTM block, this change is obvious for the output gate but not obvious for the input and output gates. These observations reveal the intrinsic mechanism of model learning under the RU: for different stages of time series (different distributions), the DL model activates different neurons to perceive data. This also shows the potential of wide neural networks for CCTS. Networks with more neurons in one layer are more likely to learn multidistributed data.

### Finding 3: Continuous prognosis reveals the disease biomarkers and stages

Semantically, the important feature is the input that has a great impact on the classification results. To quantify them, we define that the important feature is the input with a large overall importance coefficient $\alpha^{*}$. Thus, we can find biomarkers of specific diseases: as shown in Figure 4A1 and 4B1, for sepsis, the biomarkers are heart rate (HR), respiration (Resp), mean arterial pressure (MAP), PaCO2, platelets count, total bilirubin, and creatinine. For COVID-19, the biomarkers are lymphocytes (lymph), lactic dehydrogenase (LDH), high-sensitivity C-reactive protein (hs-CRP), indirect bilirubin, creatinine, etc.

**Figure 5:**
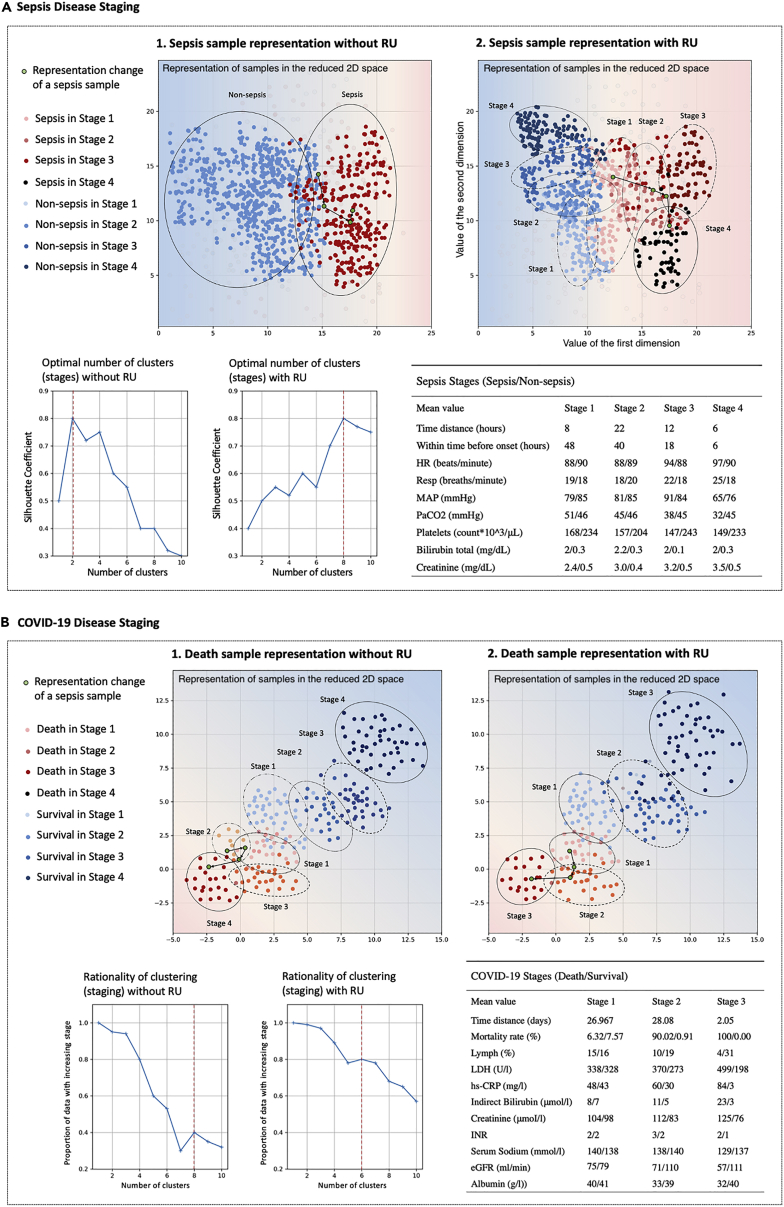
Disease stages and biomarkers of sepsis and COVID-19

The response change of the model when learning disease records continuously can reflect the disease development. After finding change points by BOCD, the training process of the model can be divided into four stages for sepsis diagnosis and three stages for COVID-19 mortality prediction. Then, as shown in Figure 5, we visualize the hidden layer of block 3 and show the statistics of the corresponding characteristics.

Sepsis has four disease stages. Each stage has different reference levels of biomarkers. In some cases, the closer to the onset time, the greater the difference in biomarker reference levels among different prognoses. For example, in stage one (the interval from early 48 h to early 40 h before the onset time), the Resp difference between sepsis and non-sepsis is 1, while in stage four (the interval from early 6 h to the onset time), the Resp difference is 7. In other cases, reference levels of biomarkers with different prognoses are different at all stages, such as creatinine. These two conditions may explain the two mechanisms of sepsis: (1) the acute sepsis onset is reflected in the changes of some specific vital signs, e.g., a drop in blood pressure, increased lactate, and tachycardia. (2) Patients who have some congenital characteristics are more likely to get sepsis, e.g., nephropathy with abnormal creatinine and hepatopathy with increased total bilirubin.

COVID-19 has three disease stages. Compared with sepsis, two classes can be distinguished more clearly in the representation space. This also explains the higher accuracy of continuous COVID-19 mortality prediction than that of continuous sepsis diagnosis. Besides, in the presentation space, the hidden features of the two classes in the later stage are further apart. This shows the difficulty in early classification: the conflict between earliness and accuracy.

The classification of eight diseases has four stages as shown in Figure S8. Since the task is not specific to a disease, we call task stages and important features instead of disease stages and biomarkers. The important features found in the four stages illustrate the necessity of continuous vital sign monitoring, blood routine examination during hospitalization, and detailed laboratory examination in later stages. Meanwhile, there is a big difference between the last three stages and the first stage. It implies that patients’ states have changed significantly since the second stage.

### Finding 4: RU enhances the model for atypical scenarios and sustainable use

The RU can avoid model overfitting and guarantee certain model generalization. As shown in Tables 1, S10, and S11, we divide datasets according to gender and age; for most baselines, the accuracy on the validation set is much lower than that on the training set, but the RU helps the DL model to maintain robustness.

**Table 1:** COVID-19 classification accuracy (AUC-ROC ↑) with non-uniform training sets and validation sets

	SR^a^^17^	ECEC^a^^18^	ORGFW^a^^19^	GEM^a^^20^	CLOPS^a^^23^	RU
Male	0.968 ± 0.014	0.969 ± 0.016	0.965 ± 0.004	0.978 ± 0.009	0.978 ± 0.014	0.971 ± 0.010
Female	0.935 ± 0.004	0.947 ± 0.015	0.938 ± 0.003	0.919 ± 0.008 ↓	0.921 ± 0.009^b^	0.947 ± 0.002^c^
Age ≤ 55	0.964 ± 0.012	0.965 ± 0.012	0.963 ± 0.007	0.971 ± 0.009	0.975 ± 0.010	0.972 ± 0.010
Age>55	0.906 ± 0.010^b^	0.908 ± 0.018^b^	0.917 ± 0.012	0.927 ± 0.010	0.907 ± 0.008^b^	0.941 ± 0.006^c^

Because indicators of blood sample and vital signs differ between males and females and change with age and the mortality rate of COVID-19 patients over 55 years old increases significantly,^35^ we train models by samples of 224 male patients/179 patients whose age is ≤55 and validate models by samples of 151 female patients/196 patients whose age is >55. In the Bonferroni-Dunn test, $k=6,n=2,m=5,q_{0.05}=2.676,N=n\times m=10,CD=2.16,\bar{r}=4.00>CD$.

a The RU is significantly better, specifically, than baselines with p = 0.0449, 0.0498, 0.0384, 0.0243, and 0.0204 (p < 0.05).

b The accuracy is greatly reduced over 5%.

c The smallest decline in accuracy.

Meanwhile, the RU can prevent the result difference caused by the different orders of training sets. The method we have introduced is to use time series of different stages to train the model, and the order is based on time. Another order is the data similarity^23^ as shown in Figure S6. For example, as many vital signs are periodic, the cycle of blood pressure is 1 day. Therefore, after using vital signs within 24 h, we will use the data within 25 h according to the time order but use the data within 48 h according to the similarity order. No matter what order is adopted, the RU has stable accuracy as shown in Tables 2, S8, and S9. It shows the potential of PM’s global optimization and the potential of the RU’s sustainable use.

**Table 2:** Classification accuracy (AUC-ROC ↑) of RU after learning training sets by different orders

		20%^a^	40%	60%	80%	100%
SEPSIS	time order	0.735 ± 0.003	0.826 ± 0.003	0.841 ± 0.003	0.860 ± 0.005	0.872 ± 0.001
	similarity order	0.734 ± 0.006	0.824 ± 0.004	0.843 ± 0.004	0.863 ± 0.007	0.870 ± 0.002
COVID-19	time order	0.789 ± 0.002	0.902 ± 0.002	0.926 ± 0.000	0.959 ± 0.001	0.968 ± 0.000
	similarity order	0.790 ± 0.001	0.910 ± 0.003	0.924 ± 0.001	0.958 ± 0.001	0.967 ± 0.000
MIMIC-III	time order	0.702 ± 0.006	0.756 ± 0.005	0.820 ± 0.007	0.852 ± 0.005	0.876 ± 0.004
	similarity order	0.704 ± 0.007	0.755 ± 0.004	0.818 ± 0.004	0.853 ± 0.004	0.876 ± 0.003

In the Bonferroni-Dunn test, $k=2,n=3,m=5,q_{0.05}=1.960,N=n\times m=15,CD=0.51,r\left(\text{time}\right)=r\left(\text{similarity}\right)=1.33<CD$. Thus, two training orders have similar effects.

a k% means the current classification time is k% of the total time of the full-length time series.

Furthermore, the RU is a data-agnostic, model-agnostic, and easy-to-use plug in. It can not only improve the accuracy of continuous classification of medical time series but also plays a role in other fields. For example, as shown in Table 3, the RU outperforms baselines on meteorological data for tasks of continuous earthquake early warning and rainfall prediction. Figures S2–S6 show the ability of the RU in more scenarios. The RU can also be used to train other DL models such as the convolutional neural network (CNN) and transformer.^36^ It is easy to use and does not need to change the network structure. As shown in Table 4, if we use the RU to train base models, the accuracy can be improved by more than 5%, and the RU is not limited by hyper-parameters. The hyper-parameters are ρ and λ. ρ determines the correlation between current and previous gradients in Equation 12. We find that PM performs well when ρ is the same as the learning rate $\rho^{m}=\eta^{m}=\frac{1}{{\left(t+1\right)}^{a}},a=1$. λ decides the constraint degree on parameter update in Equation 5. We can optimize it using the search method supplied by mature tools.

**Table 3:** Performance (AUC-ROC ↑, BWT ↑) for two meteorological datasets

		SR^a^	ECEC^a^	ORGFW^a^	GEM^a^	CLOPS^a^	RU
UCR-EQ	AUC-ROC	0.902 ± 0.002	0.909 ± 0.010	0.920 ± 0.001	0.921 ± 0.001	0.919 ± 0.004	0.931 ± 0.004^b^
	BWT	0.003	0.033	0.112	0.123	0.149	0.162^b^
USHCN	AUC-ROC	0.911 ± 0.012	0.902 ± 0.012	0.916 ± 0.004	0.920 ± 0.003	0.921 ± 0.005	0.930 ± 0.005^b^
	BWT	0.034	0.047	0.072	0.098	0.082	0.124^b^

a The UCR-EQ dataset^28^ has 471 earthquake records from the UCR time series classification archive. It is the univariate time series of seismic feature value. Natural disaster early warning, like earthquake warning, helps to reduce casualties and property losses.^37^ The USHCN dataset^27^ has the daily meteorological data of 48 states in the US from 1887 to 2014. It is the multivariate time series of five weather features. Rainfall warning is not only a demand of daily life but can also help prevent natural disasters.^38^ In the Bonferroni-Dunn test, $k=6,n=2,m=5,q_{0.05}=2.676,N=n\times m=10,CD=2.16,\bar{r}=4.00>CD$. RU is significantly better, specifically, better than baselines with p=0.0.0004,0.0241,0.0099,0.0137,and0.0329(p<0.05).

b The best performance.

**Table 4:** Performance (AUC-ROC ↑/BWT ↑) improvement of different neural networks after using RU

	SEPSIS	COVID-19	MIMIC-III	UCR-EQ	USHCN
LSTM^a^	0.837 ± 0.008/0.002	0.909 ± 0.003/0.047	0.786 ± 0.002/0.054	0.881 ± 0.004/0.032	0.891 ± 0.003/0.054
+RU	0.907 ± 0.008/0.065^b^	0.969 ± 0.003/0.115^b^	0.856 ± 0.002/0.102	**0**.931 ± 0.004/0.162^b^	0.930 ± 0.005/0.124
CNN^a^	0.848 ± 0.002/0.004	0.903 ± 0.002/0.037	0.784 ± 0.004/0.032	0.878 ± 0.005/0.030	0.881 ± 0.004/0.057
+RU	0.904 ± 0.003/0.067^b^	0.960 ± 0.006/0.095^b^	0.832 ± 0.002/0.099	0.929 ± 0.006/0.150^b^	0.922 ± 0.005/0.118
Transformer^a^	0.843 ± 0.011/0.005	0.906 ± 0.005/0.040	0.784 ± 0.006/0.059	0.889 ± 0.010/0.029	0.880 ± 0.015/0.059
+RU	0.903 ± 0.008/0.067^b^	0.960 ± 0.007/0.109^b^	0.852 ± 0.008/0.124^b^	0.920 ± 0.008/0.132	0.921 ± 0.008/0.120

a The accuracy is significantly improved after using the RU(p<0.05), specifically p=0.0040,0.0000,and0.0015.

b The accuracy is increased by more than 5%, and the BWT is increased by more than 5%.

## Discussion

### DL has the potential to explore disease mechanisms

The importance coefficient not only explained the working mechanism of the DL model but also dug out the disease biomarkers and stages.^39^ Different from the statistics and case analysis of the medical gold standard, these biomarkers are based on the judgment basis of the DL model. It can provide a new horizon for medical research. For example, based on the learning process of the DL model, for sepsis, a drop in blood pressure, increased lactate, and tachycardia are important in the later stage, while abnormal creatinine and total bilirubin are always important. It can be explained that sepsis is an acute disease and is related to some congenital diseases like nephropathy and hepatopathy. Such behavior is in exact accordance with the sepsis literature.^40^ For COVID-19, only lymph, LDH, and hs-CRP are most important throughout the stages. This shows that COVID-19 has a clear reference to measure the disease severity.^41^ Meanwhile, we match the disease stage with the change in model parameters during the learning process. In this way, the disease stage is no longer defined only by the biomarker level or patient subtyping but by the characteristic changes in the high-dimensional space created by DL.

### RU helps with disease staging

At present, except for cancers, it is difficult to define clear stages for most diseases. For sepsis, disease stratification is implemented by recommended clinical criteria (e.g., systemic inflammatory response syndrome [SIRS], sequential organ failure assessment [SOFA], quick SOFA [qSOFA], etc.), but they focus on severity and not the progression. We emphasize that disease staging is the disease change over time. The RU can achieve this according to the model change when learning the medical time series from different time stages. As shown in Figure 5A, the RU can identify stages directly according to the importance coefficient change of model parameters, instead of using unsupervised clustering methods. Without the RU, the clustering method has trouble finding the stages. The number of clusters with the best silhouette coefficient is 2, and the number of stages is 1. For COVID-19, most work categorizes it roughly into early stage and late stage.^42^ Some existing DL-based methods can perform disease staging by using representation learning. For example, our previous work^32^ clustered features in hidden layers of T-LSTM and got four COVID-19 stages. As shown in Figure 5B1, this clustering-based method can get a good silhouette coefficient but cannot guarantee the time constraint. When identifying these four stages, only about 40% of the samples will be divided into stages corresponding to chronological order. For example, a death sample (green dot) is initially judged to be stage two, then stage one, and finally stage four. But stages 1–4 are in time order. Using the RU, this inconsistency is largely alleviated: the percentage of samples with a time-increasing stage is raised. The death sample (green dot) is judged as stage one, then stage two, and finally stage three over time.

### Learning multidistributed data is the general trend

Currently, many sophisticated DL models have shown outstanding achievements in time series modeling in many fields. For offline learning, after the model has learned the dataset, the model is only sensitive to the learned distribution. For example, when the model has learned the full-length vital signs of sepsis, it usually classifies accurately at the onset time, but it is too late for critical illness. To gain treatment time, the model needs to learn early data. However, there are also problems in learning early data at only one stage. For example, a time series may have missed the learned stage at the beginning, the characteristics of early data are not obvious and require late data assistance, etc. Thus, it is necessary for the model to learn time series from different stages, i.e., multidistributed data.^43^ In this way, the model can realize CCTS.

### Reasonable training strategy is the icing on the cake

We help the DL model to learn multidistributed data from the perspective of model updating strategy. Our empirical study shows that a meaningful model training strategy plays a key role in improving the model performance and generalization power. Compared with the study of model structure design, the application of strategy-based design is more extensive. It pays more attention to the overall goal and has few requirements for specific data and used models, meaning it is data agnostic, model agnostic, and easy to use.

### Quantifying the updating process makes it possible to interpret the DL model

Interpretability remains one of the key issues to be solved to achieve the trust of clinicians and insert the DL algorithm into clinical workflow.^44^ DL models are often considered to be black box because they typically have high-dimensional non-linear operations, many model parameters, and complex model architectures, which makes them difficult for a human to understand. In this work, the RU implements CCTS by updating model parameters with constraints. The constraint is achieved by quantifying the importance of the parameters. Surprisingly, the importance of parameters can be used to explain the DL model. As shown in experimental results, it can identify both the input features and the structural parts that are important for classification. Figure S7 shows the important samples in continuous binary classification. In fact, measuring parameter changes is not only suitable for the dynamic process in CCTS but also provides the possibility to interpret the learning processes of DL models.

### Opportunities of CCTS

Currently, some subdisciplines (online learning, continual learning, anomaly detection) also study the mode of continuous classification, but their setting methods cannot satisfy the summarized requirements simultaneously (see related work in the supplemental information). CCTS is a new concept and a potential task that we propose when facing practical problems.

Meanwhile, we find that the neural network structure with a reasonable width is more conducive to continuous classification and continual learning because with the learning of the new data distribution, the change of important parameters on the scale of network width is more obvious and regular but that on the scale of depth is confused. Therefore, future work can study the impact of model structure on CCTS from the perspective of network depth and network width.

Besides, the imbalanced data are very common in many real-world datasets. For example, in electronic health records, the records of common diseases are much more than those of rare diseases. And for classification task, the lack of data in a minority class may lead to uneven accuracy. When encountering such data, although not involved in this study, we can change the cross-entropy loss L in Equation 5 to other specific losses such as weighted cross-entropy, mean false error loss, and focal loss.^45^

Further, we find that different learning orders have little effect on our method. This demonstrates the potential of our approach for offline continual learning: We use the existing data to train the model and put it into use. After a period of time, some new data may be generated. We can continue to train the current model with the new data instead of designing a new model. In addition, the method for CCTS can be context independent in the future. The model can perform not only different medical tasks but also tasks in other fields like meteorology.

## Experimental procedures

### Resource availability

#### Lead contact

Request for information and resources used in this article should be addressed to Prof. Shenda Hong (hongshenda@pku.edu.cn).

#### Materials availability

No new materials were generated by this study.

### Methods

#### CCTS

##### Definition 1 (CCTS)

A dataset $T={\left\{X^{n}\right\}}_{n=1}^{N}$ contains N time series. Each time series $X={\left\{x_{m}\right\}}_{m=1}^{M}$ has M observations with value $x_{m}$ at time $t_{m}$*.* At the final time $t_{M}$*,* X is labeled with a class C∈C. As time series vary among time, dataset $T^{*}={\left\{X_{1:m}^{n}\right\}}_{n=1}^{N}$, consisting of subsequence $X_{1:m}$ of each X, has a distribution $D_{m}$. Thus, $T_{m}^{*}={\left\{X_{1:m}^{n}\right\}}_{n,m=1}^{N,M}$ has a series distribution $D={\left\{D_{m}\right\}}_{m=1}^{M}$*.* CCTS learns every $D_{m}$ and introduces a task series $M={\left\{M^{m}\right\}}_{m=1}^{M}$ to minimize the additive risk $\sum_{m=1}^{M}E_{M^{m}}\left[L\left(f^{m}\left(D_{m};\theta \right),C\right)\right]$ with model f and parameter θ*.* $f^{m}$ is the model f after being trained for $M^{m}$, and its performance on all observed data cannot degrade: $\frac{1}{m}\sum_{i=1}^{m}L\left(f^{i},M^{i}\right)\leq \frac{1}{m-1}\sum_{i=1}^{m-1}L\left(f^{i},M^{i}\right)$*.*

Notations are summarized in Table S1. Without the loss of generality, we use the univariate time series to present the problem. Multivariate time series can be described by changing $x_{m}$ to $x_{m}^{d}$. d is the d-th dimension. The classification task uses cross-entropy loss:

(Equation 1) $$L\left(f^{m},M^{m}\right)=L\left(f^{m},\left\{T_{m}^{*},C\right\}\right)=-\frac{1}{N}\sum_{n=1}^{N}\sum_{c=1}^{\left|C\right|}C_{c}\log\;{\hat{C}}_{c}$$

#### T-LSTM

The real-world time series, especially vital signs, have long sequences and are irregularly sampled. The classical RNN only processes uniformly distributed longitudinal data by assuming that the sequences have an equal distribution of time differences. Thus, we implement T-LSTM.^32^

$$\begin{array}{l} C_{m-1}^{S}=\tanh\left(W_{d}C_{m-1}+b_{d}\right)\;\text{short}-\text{term}\;\text{memory}\\ {\hat{C}}_{m-1}^{S}=C_{m-1}^{S}\cdot g\left(\Delta^{m}\right)\;\text{discounted}\;\text{short}-\text{term}\;\text{memory}\\ C_{m-1}^{T}=C_{m-1}-C_{m-1}^{S}\;\text{long}-\text{term}\;\text{memory}\\ C_{m-1}^{*}=C_{m-1}^{T}-{\hat{C}}_{m-1}^{S}\;\text{adjusted}\;\text{previous}\;\text{memory}\\ f_{m}=\sigma \left(W_{f}x_{m}+U_{f}h_{m-1}+b_{f}\right)\;\text{forget}\;\text{gate}\\ i_{m}=\sigma \left(W_{i}x_{m}+U_{i}h_{m-1}+b_{i}\right)\;\text{input}\;\text{gate}\\ {\tilde{C}}_{m}=\tanh\left(W_{c}x_{m}+U_{c}h_{m-1}+b_{o}\right)\;\text{candidate}\;\text{memory}\\ C_{m}=f_{m}\cdot C_{m-1}^{*}+i_{m}\cdot {\tilde{C}}_{m}\;\text{current}\;\text{memory}\\ o_{m}=\sigma \left(W_{o}x_{m}+U_{o}h_{m-1}+b_{o}\right)\;\text{output}\;\text{gate}\\ h_{m}=o_{m}\cdot \tanh\left(C_{m}\right)\;\text{current}\;\text{hidden}\;\text{state} \end{array}$$

T-LSTM has some new designs. The $C_{m-1}^{S}$ component learns the short-term memory of a sequence by learnable network parameters. $C_{m-1}^{T}$ is the long-term memory calculated from the former memory cell $C_{m-1}$ by getting rid of $C_{m-1}^{S}$. $C_{m-1}^{S}$ is adjusted to the discounted short-term memory ${\hat{C}}_{m-1}^{S}$ by the elapsed time function $g\left(\Delta_{m}\right)$. The previous memory $C_{m-1}^{*}$ is changed to the complement subspace of $C_{m-1}^{T}$ combined with ${\hat{C}}_{m-1}^{S}$. We use a log calculation for the elapsed time function. $\Delta_{m}$ describes the time gap between two records at two adjacent time points $t_{m}$ and $t_{m-1}$.

(Equation 2) $$g\left(\Delta_{m}\right)=\frac{1}{\log\left(e+\Delta_{m}\right)},\Delta_{m}=t_{m}-t_{m-1}$$

#### RU

##### LM

When the model meets a distribution, it will change from $f^{m-1}$ to $f^{m}$. In order to let the model performance on all tasks not degrade, the loss L of the current $f^{m}$ on tasks ${\left\{M^{k}\right\}}_{k=1}^{m}$ should be not bigger than that of the previous $f^{m-1}$ on tasks ${\left\{M^{k}\right\}}_{k=1}^{m-1}$:

(Equation 3) $$\begin{array}{c} \min_{\theta^{m}}\;L\left(f^{m}\left(X_{1:m},\theta^{m}\right),C\right)\\ \text{subject}\;\text{to}\;\frac{1}{m}\sum_{k=1}^{m}L\left(f^{m},M^{k}\right)\leq \frac{1}{m-1}\sum_{k=1}^{m-1}L\left(f^{m-1},M^{k}\right) \end{array}$$

Based on the observation of many parameter configurations resulting in the same performance, we could add a regular term to the loss to restrict the updating of model parameters. Thus, in LM, we constrain important parameters to stay close to their old values but change $\theta_{\text{unimportant}}$ more. We give a new loss in Equation 4. A regularization term is added to the original loss L, where α is the importance coefficient of parameter θ. With the minimum O, $\theta^{m}$ will be changed less from $\theta^{m-1}$ with a large α. In Figure 2, $\theta_{\text{important}}$ is limited in a region.

(Equation 4) $$O\left(\theta^{m}\right)=L\left(f^{m}\left(\theta^{m}\right),M^{m}\right)+\lambda \sum_{i}\alpha_{i}{\left(\theta_{i}^{m}-\theta_{i}^{m-1}\right)}^{2}$$

The second derivative of probability can evaluate the importance coefficient $\alpha ={\left(\log\;p\;\left(D_{m}|0^{m}\right)\right)}^{\prime \prime }$. Elastic weight consolidation^21^ defines α from a probabilistic perspective $\log\;p\left(\theta |D\right)=\log\;p\left(D_{m}|\theta \right)+\log\;p\left(\theta |D_{m-1}\right)-\log\;p\left(D_{m}\right)$. Optimizing the parameters is tantamount to finding their most probable values under D. The posterior probability is indicated by Laplace approximation. We use the diagonal of a Fisher information matrix to represent the first-order derivatives $F=\frac{1}{N}\sum_{k=1}^{N}\nabla \log\;p\left(D_{k}|\theta \right)\nabla \log\;p{\left(D_{k}|\theta \right)}^{⊤}$, represent α by F, and re-arrange Equation 4 to

(Equation 5) $$\begin{array}{c} O\left(\theta^{m}\right)=L\left(f^{m}\left(\theta^{m}\right),M^{m}\right)+\lambda \sum_{i}F_{i}{\left(\theta_{i}^{m}-\theta_{i}^{m-1}\right)}^{2}\\ F_{i}=\frac{1}{m}\sum_{k=1}^{m}{\left(\frac{\partial \log\;p\left(D^{k}|\theta_{i}^{m}\right)}{\partial \theta_{i}^{m}}\right)}^{2} \end{array}$$

##### PM

When we focus on the final task, if the model meets a new distribution, the learned knowledge will be part of the final solution. We regard this as a continuous optimization problem and treat different data distributions equally. The new data helps the model learn the old data, which can reduce the unstable solution caused by the different learning orders. The continuous optimization problem is defined as regret minimization. For task M, the regret R is the difference between the total loss and that of the best parameter $\theta^{*}$ of the fixed decision in hindsight.

(Equation 6) $$R_{M}:=\sum_{m=1}^{M}(L(f^{m}(\theta^{m}),M)-L(f^{m}(\theta^{m*}),M))$$

For regret minimization, we design PM by projection-free mechanisms and stochastic recursive gradient. It focuses the quality of the final performance instead of iterates produced from the course of optimization. The main bottleneck is the computation of projections onto the underlying decision set $\prod_{K}\left(\theta \right)=\underset{\hat{\theta }\in K}{\mathrm{argmin}}\left\|\hat{\theta }-\theta \right\|$. The projection operation is defined as the closest point inside the convex set K of Euclidean space to a given point. The projection-free method can replace the projection with a linear optimization at each iteration. It alleviates the complexity but remains problems of training non-converging and instability.

Thus, we estimate a stochastic recursive estimator based on stochastic gradient technology.^19^ Assuming for task $M^{m}$, the model receives a new distribution $D_{m}$ and gets the loss L. We first give a random variable $\xi^{m}$ to satisfy $E_{\xi^{m}∼D_{m}}\left[\nabla L\left(\theta^{m},\xi^{m}\right)\right]=\nabla \sum_{m=1}^{M}L\left(f^{m}\left(X_{1:m},\theta^{m}\right),C\right)$. Then, the stochastic recursive estimator is

(Equation 7) $$d^{m}=\nabla L\left(\theta^{m},\xi^{m}\right)+\left(1-\rho^{m}\right)\left(d^{m-1}-\nabla L\left(\theta^{m-1},\xi^{m}\right)\right)$$

It finds a solution $v^{m}$ of the linear optimization problem

(Equation 8) $$v^{m}=\underset{v\in K}{\mathrm{argmin}}{\left\|d^{m},v^{m}\right\|}_{2}$$

to update $\theta^{m}$ in the direction of gradient $g^{m}$:

(Equation 9) $$g^{m}=v^{m-1}-\theta^{m-1},\theta^{m}=\theta^{m-1}+\eta^{m-1}\cdot g^{m}$$

Such a method randomly selects samples to guide the change of gradient and leads to faster converges.

##### Overall training process

When the new gradient $g_{m}$ and the old gradient $g_{k}$ are at an acute angle, the model performance dose not decrease and even improves^20^:

(Equation 10) $$\begin{array}{l} \left\langle g^{m},g^{k}\right\rangle =\left\langle \frac{\partial L\left(f^{m},M^{m}\right)}{\partial \theta^{m}},\frac{\partial L\left(f^{k},M^{k}\right)}{\partial \theta^{k}}\right\rangle \geq 0\\ k=1,\ldots ,m-1 \end{array}$$

The regularization projects $g_{m}$ to the closest gradient $g_{m}^{\prime }$ by satisfying all the constraint of acute angle:

(Equation 11) $$\begin{array}{c} \min\;{\left\|g^{m}-g^{m^{\prime }}\right\|}_{2}\\ \text{subject}\;\text{to}\;\left\langle g^{k},g^{m^{\prime }}\right\rangle \geq 0,k=1,\ldots ,m-1 \end{array}$$

In LM, F is positive semi-definite. This property not only guarantees that seeing each task as a factor of the posterior (LM) but also guarantees the acute angle change of a vector after the product (PM). Thus, the RU updates network parameters by using the regularized loss O in Equation 5, and we re-arrange Equations 7, 8, 9, 10, 11, and 12. $\eta^{m},\rho^{m}={\left(\frac{1}{m+1}\right)}^{a}$.

(Equation 12) $$d^{m}\leftarrow \nabla_{\theta^{m}}O^{m}+\left(1-\rho^{m}\right)\left(d^{m-1}-g^{m-1}\right)$$

Algorithm 1 gives the algorithm description of RU. In practice, we optimize hyper-parameters using the search method supplied by mature tools. Figure S5 shows that the method performs better when a=0.933λ+0.907.

Algorithm 1 **RU**

**Table:** 

Input:The DL model f (T-LSTM) and a defined task set M.
Output:The trained model f
1: Initialize a model $f^{0}$ with parameter $\theta^{0}$
2: Initialize a gradient memory G←{}
3: Initialize parameters $\eta^{m},\rho^{m}={\left(\frac{1}{m+1}\right)}^{a}$
4: for m=1 to |M| do
5: Extract current task $M^{m}\leftarrow M$
6: Get loss $O^{m}\leftarrow$ Equation 5 ⊳_Limitation Mechanism_
7: $g^{m-1}\leftarrow G$
8: $d^{m}\leftarrow$ Equation 12
9: $v^{m}\leftarrow$ Equation 8
10: $g^{m},\theta^{m}\leftarrow$ Equation 9 ⊳_Promotion Mechanism_
11: $G\leftarrow g^{m}$
12: Get model $f^{m}\leftarrow \theta^{m}$
13: end for
14: Output model $f\leftarrow f^{\left|M\right|}$

##### Regret and complexity

PM holds a nearly optimal regret bound $\tilde{O}\left(\sqrt{M}\right)$ with probability at least 1−δ for any δ∈(0,1), $R_{M}\leq \left(logM+1\right)\left(f\left(\theta^{1}\right)-f\left(\theta^{*}\right)\right)+\left(16LD^{2}+16\sigma +4B\right)\sqrt{2Mlog\frac{8M}{\delta }}+\frac{1}{2}LD^{2}{\left(logM+1\right)}^{2}$. D is diameter of convex set, and L is L-Lipschitz continuous. PM and LM achieve a O(1) per-round computational cost. If the complexity of training a base model to convergence is O and data length is M, the overall complexity will be MO (see supplemental mathematics).

#### Evaluation metrics

The classification accuracy is evaluated by assessing the AUC-ROC (the higher the better). The ROC is a curve of the true positive rate (TPR) and the false positive rate (FPR). TN, TP, FP, and FN represent true positive, true negative, false positive, and false negative, respectively.

(Equation 13) $$\text{TPR}=\frac{\text{TP}}{\text{TP}+\text{FN}},FPR=\frac{\text{FP}}{\text{TN}+\text{FP}}$$

The AUC confidence interval is equal to $AUC\pm se\cdot z_{\text{crit}}$. $z_{\text{crit}}$ is the two-tailed critical value of the standard normal distribution NORM.S.INV(1−α/2),α=0.05. se is Equation 14, where $s_{1}$ and $s_{2}$ are the sizes of the two samples in different labels and $q_{0}=AUC\left(1-AUC\right),q_{1}=AUC/\left(2-AUC\right)-AUC^{2},q_{2}=2AUC^{2}/\left(1+AUC\right)-AUC^{2}$. For example, in the SEPSIS dataset, s1=27,977 (non-sepsis) and s2=2,359 (sepsis), and in COVID-19 dataset, s1=201 (survival) and s2=174 (death).

(Equation 14) $$se=\sqrt{\frac{q_{0}+\left(n_{1}-1\right)q_{1}+\left(n_{2}-1\right)q_{2}}{n_{1}n_{2}}}$$

The statistical significance is evaluated by the Bonferroni-Dunn test, and k,n,m,andq are the number of methods, the number of datasets, the number of cross-validation folds, and the critical value, respectively. If the average rank of baselines $\bar{r}>CD$, the tested method is significantly better.

(Equation 15) $$\text{CD}=q\sqrt{\frac{k\left(k+1\right)}{6N}},N=n\times m$$

Learning stability is evaluated by the gradient fluctuation R (the lower the better). It quantifies the frequency of gradient direction changes during training.

(Equation 16) $$R=\frac{1}{n-1}\sqrt{\sum_{i=1}^{n}{\left(d_{i}-d_{i-1}\right)}^{2}},d=\left\{ \begin{array}{c} -1,\text{if}\;g<0\\ 1,\text{if}\;g>0 \end{array}\right.$$

The continuous classification performance is evaluated by the BWT and FWT (the higher the better). They are the influences that learning a new distribution have on old and future distributions. $R_{i,j}$ is the accuracy on distribution $D_{j}$ after completing task $D_{i}$. $\bar{b}$ is the accuracy with random initialization.

(Equation 17) $$\begin{array}{cc} \text{BWT} & =\frac{1}{\left|D\right|-1}\sum_{i=1}^{\left|D\right|-1}R_{\left|D\right|,i}-R_{i,i}\\ \text{FWT} & =\frac{1}{\left|D\right|-1}\sum_{i=2}^{\left|D\right|}R_{i-1,i}-{\bar{b}}_{i,i} \end{array}$$

## Data Availability

All datasets are publicly available. SEPSIS: https://doi.org/10.13026/v64v-d857,^24^ COVID-19: https://doi.org/10.1038/s42256-020-0180-7,^25^ MIMIC-III: https://doi.org/10.13026/C2XW26,^26^ USHCN: NCDC (DSI-3200, DSI-3206 and DSI-3210),^27^ UCR https://www.cs.ucr.edu/∼eamonn/time_series_data_2018/,^28^ and ACTIV: https://archive.ics.uci.edu/ml/datasets/Localization+Data+for+Person+Activity.^29^ All original code has been deposited at Zenodo under https://doi.org/10.5281/zenodo.7496021 (https://github.com/SCXsunchenxi/CCTS) and is publicly available as of the date of publication. Any additional information required to reanalyze the data reported in this paper is available from the lead contact upon request.

## Supplemental information

Document S1. Supplemental experimental procedures, Figures S1–S8, and Tables S1–S11

Document S2. Article plus supplemental information
