# Supplementary material for: Continuous diagnosis and prognosis by controlling the update process of deep neural networks
Source: Patterns (N Y). 2023 Feb 3;4(2):100687. doi: 10.1016/j.patter.2023.100687 (PMC9982300; doi:10.1016/j.patter.2023.100687)
Supplement: Document S1. Supplemental experimental procedures, Figures S1–S8, and Tables S1–S11 [file mmc1.pdf]

Patterns, Volume *4*

Supplemental information

Continuous diagnosis and prognosis by controlling the update process of deep neural networks

Chenxi Sun, Hongyan Li, Moxian Song, Derun Cai, Baofeng Zhang, and Shenda Hong

Supplemental Information

1. Supplemental Related Work and Concepts

Time series is one of the most common data forms, the popularity of time series classification has attracted increasing attention in many practical fields, such as healthcare and industry. In the real world, the class of a time series is usually labeled at the final time. For example, patients' outcomes will come at the end. Most deep learning (DL) models are good at single-shot classification, classifying data at a fixed time after learning time series within a fixed period. Because DL methods assume that the observed data is independent and identically distributed (i.i.d) and subsequences in the same period maintain one distribution.

However, in the real world, more and more time-sensitive applications need to classify time series continuously before the final labeled time. For example, in the intensive care unit (ICU), diagnosis and prognosis are needed at any time to provide more opportunities for doctors to rescue lives. Each hour of delay has been associated with roughly a 4-8% increase in sepsis mortality. But patient labels, e.g. mortality or morbidity, are only available at the onset time but unknown in the early stages. In response to the current demand, we propose a new concept – Continuous Classification of Time Series (CCTS), to classify time series at every time point before the labeled time. For example, using vital signs like blood pressure to diagnose patients continuously.

1.1. Single-shot Classification

Single-shot classification methods classify at a fixed time. A time series $X = \{x_m\}_{m=1}^{M}$ is labeled with classes C. Single-shot classification aims to classify X at a time $t_m, m \leq M$ with the minimum loss $\mathcal{L}(f(X_{1:m}), C)$.

The foundation is the Classification of Time Series (CTS), making classification based on the full-length data. But in time-sensitive applications, Early Classification of Time Series (ECTS), classifying at an early time, is more critical. For example, early diagnosis helps for sepsis outcomes. Nowadays, Recurrent Neural Networks (RNNs) and Convolutional Neural Networks (CNNs) have shown good performances for CTS and ECTS by modeling long-term dependencies, addressing data irregularities, learning frequency features, etc.

Definition 1 (Classification of Time Series, CTS). *A dataset $\mathcal{D} = \{(X^n, C^n)\}_{n=1}^{N}$ has N time series. Each time series X is labeled with a class C, CTS classifies time series using the full-length data by model $f : X \rightarrow C$*

Definition 2 (Early Classification of Time Series, ECTS). *A dataset of time series $\mathcal{D} = \{(X^n, C^n)\}_{n=1}^{N}$ has N samples. Each time series $X = \{x_m\}_{m=1}^{M}$ is labeled with a class C. ECTS classifies time series in an advanced time t_m by model $f : \{X_{1:m}\} \rightarrow C$, where $m < M$.*

As shown in Figure s1, the existing classification tasks of time series are the single-shot classification, where the classification is performed only once at the final or an early time. However, many real-world applications require continuous classification. For example, intensive care patients should be detected and diagnosed at all times to facilitate timely life-saving. The above methods only classify once and just lean a single data distribution. They have good performances on i.i.d data at a fixed time, like early 6 hours sepsis diagnosis, but fail for multi-distribution. In fact, continuous classification is composed of multiple single-shot classifications.

1.2. Continuous Classification

Without the loss of generality, we use the univariate time series to present the problem. Multivariate time series can be described by changing x_m to x_m^d. d is the d-th dimension. Note that single-shot classification optimizes the objective with a single loss $\mathcal{L}(f(x), c)$. RU should consider the multi-distribution and classify more times.

Definition 3 (Continuous Classification, CC). *A dataset of time series $\mathcal{D} = \{(X^n, C^n)\}_{n=1}^{N}$ has N samples. Each time series $X = \{X_m\}_{m=1}^{M}$ is labeled with a class C. CC classifies time series at every advanced time $t_m, m = 1, ..., M$ by model $f : \{X_{1:m}\}_{m=1}^{M} \rightarrow C$.*

We summarize the four requirements of continuous diagnosis and prognosis in Introduction section into three technical requirements of Continuous Classification of Time Series (CCTS).

Table s1
Notations and Description

Notation	Description
$\mathcal{T}$, $\mathcal{T}^*$	Time series dataset
$\mathcal{D}$	Distribution set
$\mathcal{M}$	Task series
$\mathcal{L}$	Classification loss
$\mathcal{O}$	Objective function
X, C	A time series sample and its class
x_m, t_m	The m-th observed value and time of X
f	Classification model
θ	Model parameters
g	Gradient
α	Importance coefficient

Requirement 1: the ability to model multi-distributed data. In the real-world dataset, the label of a time series is usually determined at the final time. The DL model trained by this dataset can only give the single-shot classification at the onset time after observing the full-length time series. For CCTS, the model need to learn time series from different advanced stages so that it can classify at every time: When the data changes, the model performance needs to maintain, and accuracy cannot reduce.

Requirement 2: potential for earlier classification with guaranteed accuracy. Early classification is necessary for many time-sensitive applications. For example, early detection is critical for sepsis, where each hour of delayed treatment has been associated with roughly an 4-8% increase in mortality[1]. But basic questions about the limits of early detection remain unanswered. If one wants to pursue higher accuracy, the model would tend to predict late as it wants to observe more data.

Requirement 3: offline learning and sustainable use. In many time-sensitive and privacy-related scenarios, we need to directly use the well-trained model instead of updating it while using it. Because if the model is updated in real time, it will lead to the unsupervised learning mode caused by the lack of timely access to labels and finally increase the risk of misjudgment[2]. Further, in subsequent applications, when a batch of new data is obtained, we hope to continue to use the current model instead of designing a new model. Because the data which has the old knowledge may still occur, while the new system cannot handle the old knowledge well.

Most methods use multi-model to learn multi-distribution, like SR[3] and ECEC[4]. They divide data by time stages and design different classifiers for different distributions. But the operation of data division and classifier selection will cause additional losses.

Currently, some sub-disciplines also study the mode of continuous learning or continuous classification. But their setting does not match our needs and their methods can't address our issues, as shown in Figure s2.

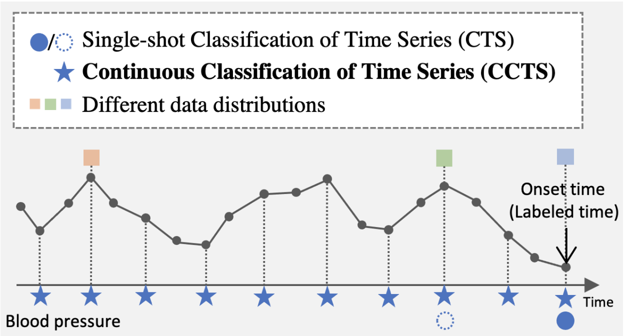

Figure s1: Continuous Classification of Time Series (CCTS)

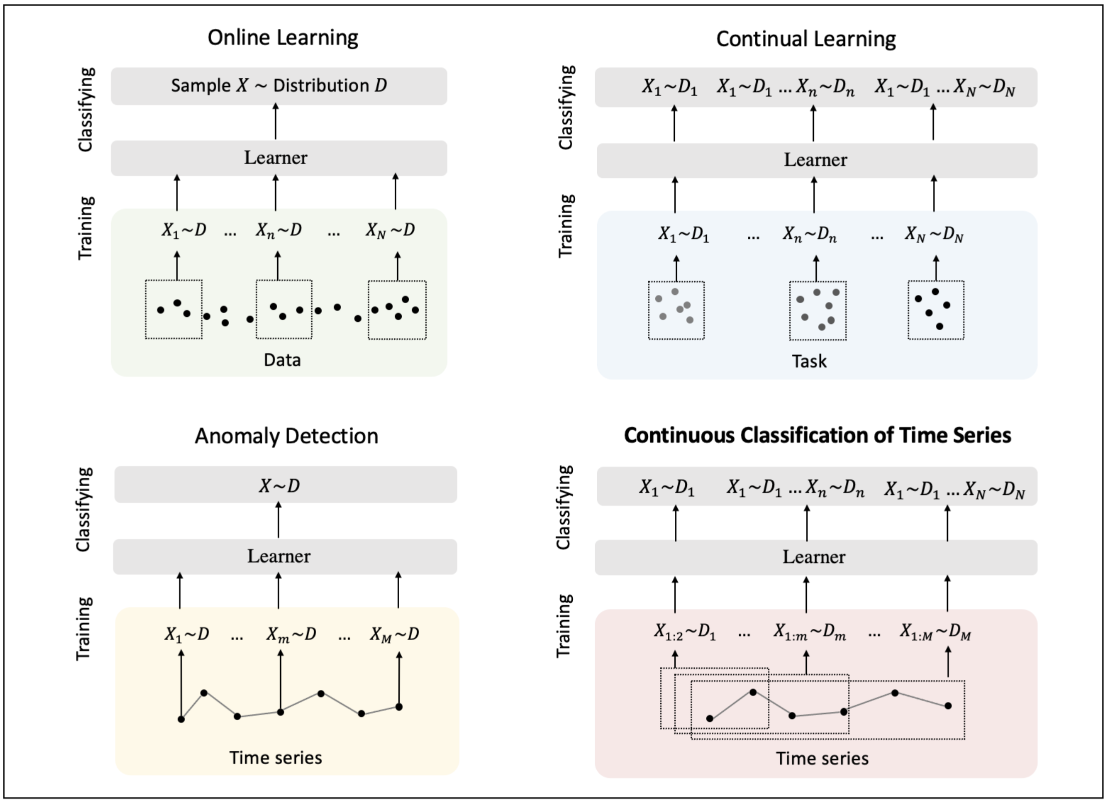

Figure s2: Differences and Similarities between CCTS and Other Concepts

Definition 4 (Online Learning, OL). *A OL issue has a sequence of dataset $\mathcal{T} = \{\mathcal{T}^n\}_{n=1}^N$ for one task $\mathcal{M}$. All datasets has the same distribution D. The goal is to find the optimal solution of $\mathcal{M}$ after N iterations by minimize the regret $\mathcal{R} := \sum_{n=1}^N (f^n(\mathcal{T}^n) - \min f^n(\mathcal{T}^n))$.*

Online Learning (OL) models the incoming data steam continuously to solve an overall optimization problem with the partially observed data. It focuses more on issues in data steam, rather than the dynamics of time series. Most importantly, it maintains only one data distribution, rather than learning multiple. Thus, OL cannot meet the Requirement 1 and Requirement 2.

Definition 5 (Continual Learning, CL). *A CL issue $\mathcal{M} = \{\mathcal{M}^m\}_{m=1}^M$ has a sequence of M tasks. Each task $\mathcal{M}^m$ has a dataset $\mathcal{T}^m = \{X^n, C^n\}_{n=1}^N$ with N samples and corresponding class labels. CL learns a new task at every moment. The goal is to control the statistical risk of all seen tasks $\sum_{m=1}^M \mathbb{E}_{(X,C)\in\mathcal{T}^m}[\mathcal{L}(f((X;\theta), C)]$ with loss $\mathcal{L}$, network function f and parameters θ.*

Continual Learning (CL) enables the model to learn new tasks over time without forgetting the old tasks. It learns a new task at every new moment and each new task corresponds to a new data distribution. Replay-based methods re-train the model by the old data to consolidate memory[5]; Regularization-based methods restrain parameter update of neural networks to limit forgetting[6]; Model-based methods change network structure or apply multiple models to response to different tasks[7]. But most of the above methods have the problems of storage limitation, distributions drifts and model overfitting. Most importantly, in CL, the definition of old and new tasks is clear and the division of distribution is fixed. But in CCTS, the distributions, that is, the tasks in CL, is not determined and need to be defined. While the dynamic time series has data correlation over time, which easily further causes the overfitting problem. Thus, CL cannot meet the partial Requirement 1 and the Requirement 2.

Definition 6 (Anomaly Detection, AD). *A sequence $X = \{X_n\}_{n=1}^{N}$ has N observations and maintains one data distribution D. AD task aims to find the abnormal observation X_a in X by evaluating whether X_a deviates from D.*

Anomaly Detection (AD) identifies data that does not conform to the expected pattern. It mainly maintains one data distribution and gives an alarm when an exception occurs. Thus, AD cannot meet Requirement 1 and partial Requirement 2.

Because the existing research can not meet the current demand, we propose a new concept CCTS.

2. Supplemental Mathematics

Assumption 1. *The compact convex set $C \subseteq \mathbb{R}^d$ has diameter D. $\forall \theta_1, \theta_2 \in C$,*

$$||\theta_1 - \theta_2|| \leq D \tag{1}$$

Assumption 2. *The stochastic gradient $\nabla F^m(\theta, \xi^m)$ is unbiased with $\mathbb{E}_{\xi^m}[\nabla F^m(\theta, \xi^m)] = \nabla f^m(\theta)$ and is L-Lipschitz continuous over the constraint set C with*

$$||\nabla F^m(\theta_1, \xi^m) = \nabla F^m(\theta_{2,\xi^m})|| \leq L||\theta_1 = \theta_2||, \forall \theta_1, \theta_2 \in C. \tag{2}$$

The above Assumption immediately implies that f^m is differentiable and has L-Lipschitz-continuous gradients.

In the stochastic online setting, we denote the expected loss function as the $\overline{f} = \mathbb{E}_{f^m \sim \mathcal{D}[f^m]}$. In order to obtain high probability results. We assume the following:

Assumption 3. *The distance between the stochastic gradient $\nabla F^m(\theta, \xi^m)$ and the exact gradient is bounded over the constraint set C, for any $theta \in C, t \in \{1, ..., T\}$, there exist $\sigma < \infty$ such that with probability 1,*

$$||\nabla F^m(\theta, \xi^m) - \nabla \overline{f}(\theta)||^2 \leq \sigma^2 \tag{3}$$

The difference of $f^m(\theta)$ and $\overline{f}^m(\theta)$ is bounded over the constraint set C. $\forall \theta \in C, t \in \{1, ..., T\}$, there exist $M^2 < \infty$ such that with probability 1,

$$|f(\theta) - \overline{f}(\theta)| \leq M^2 \tag{4}$$

We show that the norm of the gradient estimation error $\varepsilon^m := d^m - \nabla \overline{f}(\theta^m)$ converges to zero rapidly w.h.p.

First, we reformulate ε^m as the sum of a martingale difference sequence $\{\epsilon_{m,k}\}_{k=1}^{m}$ w.r.t. a filtration $\{\mathcal{F}^m\}_{k=0}^{m}$, i.e., $\varepsilon^m = \sum_{k=1}^{m} \epsilon_{m,k}$, where $\mathbb{E}[\epsilon_{m,k}|\mathcal{F}_{m-1}|] = 0$ and $\mathcal{F}_{m-1}$ is the θ-filed generate by $\{f_i, \xi_i\}_{i=1}^{k-1}$. By showing that $||\epsilon_{m,k}|| \leq c_{m,k}$ for some constant $c_{m,k}$, one can relate the Hoeffding-type concentration inequality. With carefully chosen $\{\rho^m\}_{m=1}^{M}$ and $\{\eta^m\}_{m=1}^{M}$, the quantity q^m can be shown to converge to 0 at a sublinear rate by induction. As a result, $||\varepsilon^m||$ converges to zero at a sublinear rate w.h.p. as stated in the following lemma. D is diameter of convex set, L is L-Lipschitz-continuous.

Lemma 1. *With $\rho^m = \eta^m = \frac{1}{(1+m)^a}$ for some $a \in (, 1]$, if Assumptions are satisfied for any $t \geq 1$ and $\delta_0 \in (0, 1)$ we have w.p.at least $1 - \delta_0$,*

$$||\varepsilon^m|| \leq 2(2LD) + \frac{3^a \sigma}{3^a - 1}(m+1)^{-\frac{a}{2}} \sqrt{2 \log(\frac{4}{\delta_0})}. \tag{5}$$

Lemma 1 shows that the gradient approximation error $||\varepsilon^m||$ converges to zero at a fast sublinear rate $\tilde{\mathcal{O}}(\frac{1}{m^{\frac{a}{2}}})$ w.h.p if $\rho^m = \eta^m = \frac{1}{(1+m)^a}$ for any $a \in (0, 1]$. This result is critical to the regret analysis of our methods.

Theorem 1. *With* $\rho^m = \eta^m = \frac{1}{1+m}$. *If* $\overline{f}$ *is convex and Assumption are satisfied, then w.p. at least* $1 - \delta$ *for any* $\delta \in (0, 1)$ *for any* $\delta \in (0, 1)$,

$$\begin{aligned}\mathcal{R}^m \leq & (logM + 1)(f(\theta^1) - f(\theta^*)) \\ & + (16LD^2 + 16\sigma + 4B)\sqrt{2Mlog\frac{8M}{\delta}} \\ & + \frac{1}{2}LD^2(logM + 1)^2\end{aligned} \tag{6}$$

3. Supplemental Experimental Procedures

3.1. Datasets

- SEPSIS dataset[8] has 30,336 records with 2,359 diagnosed sepsis. Early diagnose is critical to improve sepsis outcome[1]. In this dataset, the time series are the changes of 40 related patient features, the label at each time is sepsis or non-sepsis. Early diagnose can improve sepsis outcome.
- COVID-19 dataset[9] has 6,877 blood samples of 485 COVID-19 patients from Tongji Hospital, Wuhan, China. Mortality prediction helps for treatment and rational resource allocation[10]. In this dataset, the time series are the changes of blood samples, the label at each time is mortality or survival. Mortality prediction helps for personalized treatment and rational resource allocation
- MIMIC-III dataset[11] has 19,993 admission records of 7,537 patients. We focus on 8 diagnoses (ICD-9): Diabetes(249), Hypertension (401), Heart Failure (428), Pneumonia (480-486), Gastric Ulcer (531), Hepatopathy (571), Nephropathy (580-589), SIRS (995.9). The time series are vital signs, and labels at each time are some diagnoses.
- USHCN dataset[12] has the daily meteorological data of 48 states in U.S. from 1887 to 2014. We focus on 4 weather conditions in New York: sunny, overcast, rainfall, snowfall. The time series are records of 4 neighboring states, labels at each time are weather after a weak. Rainfall warning is not only the demand of daily life, but also can help prevent natural disasters.
- UCR time series classification archive[13] consists of 128 time series datasets. We have selected 15 datasets covering multiple data types (spectro, sensor, image, motion, simulated) and classification tasks (binary-, three-, four-, five-, six-classification).
- UCR-EQ dataset has 471 earthquake records from UCR time series database archive. It is the univariate time series of seismic feature value. Natural disaster early warning, like earthquake warning, helps to reduce casualties and property losses.
- ACTIV dataset[14] consists of 3D positions collected from five individuals performing various activities including walking, sitting, lying, standing, etc. Each sample is a multivariate time series from 4 sensors and we focus on 11 types of activities.

3.2. Baselines

- ECTS-based methods:
  - LSTM trains a model by learning time series at every time stage.
  - SR[3] gives the fusion result of multiple models trained by the full-length data
  - ECEC[4] has trains a set of classifiers by data in different time stages.
- CL-based methods:
  - EWC[15] is a regularization-based method, training a model to remember the old tasks by constraining important parameters to stay close to their old values.

  - GEM[6] is a regularization-based method, updating parameters by finding gradients which are at acute angles to old gradients
  - CLEAR[16] is a replay-based method, using the reservoir sampling to limit the number of stored samples to a fixed budget.
  - CLOPS[5] is a replay-based method, re-learning old tasks wshen forgetting appears.

- OL-based methods:
  - OSFW[17] uses stochastic gradient estimator
  - ORGFW[18] uses recursive gradient estimator.

References

1. Seymour, C.W., Gesten, F., Prescott, H.C. (2017). Time to treatment and mortality during mandated emergency care for sepsis. New England Journal of Medicine 376, 2235–2244. 10.1016/j.jemermed.2017.08.088.
2. Si, N., Zhang, F., Zhou, Z., Blanchet, J. (2020). Distributionally robust policy evaluation and learning in offline contextual bandits. In: Proceedings of the International Conference on Machine Learning (ICML), pp. 8884–8894.
3. Mori, U., Mendiburu, A., Dasgupta, S., Lozano, J.A. (2018). Early classification of time series by simultaneously optimizing the accuracy and earliness. IEEE Transactions on Neural Networks and Learning Systems 29, 4569–4578. 10.1109/TNNLS.2017.2764939.
4. Lv, J., Hu, X., Li, L., Li, P. (2019). An effective confidence-based early classification of time series. IEEE Access 7, 96113–96124. 10.1109/ACCESS.2019.2929644.
5. Kiyasseh, D., Zhu, T., Clifton, D. (2021). A clinical deep learning framework for continually learning from cardiac signals across diseases, time, modalities, and institutions. Nature Communications 12, 4221. 10.1038/s41467-021-24483-0.
6. Lopez-Paz, D., Ranzato, M. (2017). Gradient episodic memory for continual learning. In: Proceedings of the Advances in Neural Information Processing Systems (NeurIPS), pp. 6467–6476.
7. Mallya, A., Lazebnik, S. (June 2018). Packnet: Adding multiple tasks to a single network by iterative pruning. In: Proceedings of the IEEE Conference on Computer Vision and Pattern Recognition (CVPR), pp. 7765–7773. 10.1109/CVPR.2018.00810.
8. Reyna, M.A., Josef, C., Seyedi, S., Jeter, R. (2019). Early prediction of sepsis from clinical data: the physionet/computing in cardiology challenge 2019. In: Computing in Cardiology, pp. 1–4. 10.23919/CinC49843.2019.9005736.
9. Yan, L., Zhang, H.T., Goncalves, J., Xiao, Y., Wang, M., Guo, Y., Sun, C., Tang, X., Jing, L., Zhang, M., et al. (2020). An interpretable mortality prediction model for covid-19 patients. Nature Machine Intelligence 2, 283–288. 10.1038/s42256-020-0180-7.
10. Sun, C., Hong, S., Song, M., Li, H., Wang, Z. (2020). Predicting covid-19 disease progression and patient outcomes based on temporal deep learning. BMC Medical Informatics and Decision Making 21, 45. 10.1186/s12911-020-01359-9.
11. Johnson, A.E., Pollard, T.J., Shen, L., Li-wei, H.L., Feng, M., Ghassemi, M. (2016). Mimic-iii, a freely accessible critical care database. Scientific Data 3, 160035. 10.1038/sdata.2016.35.
12. Menne, M., Williams Jr, C., Vose, R.. Long-term daily and monthly climate records from stations across the contiguous united states (us historical climatology network)(ndp-019). Tech. Rep., Environmental System Science Data Infrastructure for a Virtual Ecosystem (2016). 10.3334/CDIAC/CLI.NDP019.
13. Dau, H.A., Bagnall, A., Kamgar, K., Yeh, C.C.M., Zhu, Y., Gharghabi, S., Ratanamahatana, C.A., Keogh, E. (2019). The ucr time series archive. IEEE/CAA Journal of Automatica Sinica 6, 1293–1305. 10.1109/JAS.2019.1911747.
14. Rubanova, Y., Chen, T.Q., Duvenaud, D. (2019). Latent ordinary differential equations for irregularly-sampled time series. In: Proceedings of the Advances in Neural Information Processing Systems (NeurIPS), pp. 5321–5331.
15. Kirkpatrick, J., Pascanu, R., Rabinowitz, N.C., Veness, J., Desjardins, G., Rusu, A.A. (2017). Overcoming catastrophic forgetting in neural networks. Proceedings of the National Academy of Sciences 114, 3521–3526. 10.1073/pnas.1611835114.
16. Rolnick, D., Ahuja, A., Schwarz, J., Lillicrap, T.P., Wayne, G. (2019). Experience replay for continual learning. In: Proceedings of the Advances in Neural Information Processing Systems (NeurIPS), pp. 348–358.
17. Chen, L., Harshaw, C., Hassani, H., Karbasi, A. (2018). Projection-free online optimization with stochastic gradient: From convexity to submodularity. In: Proceedings of International Conference on Machine Learning (ICML), pp. 814–823.
18. Xie, J., Shen, Z., Zhang, C. (2020). Efficient projection-free online methods with stochastic recursive gradient. In: Proceedings of the AAAI Conference on Artificial Intelligence (AAAI), pp. 6446–6453. 10.1609/aaai.v34i04.6116.

Table s2
Classification Accuracy (AUC-ROC↑) at the First 5 Time Steps
*k% means the current classification time is k% of the total time of the full-length time series; Bold font indicates the highest accuracy.
Our method is significantly better than baselines by using Bonferroni-Dunn test. RU can classify more accurately at every time. The average accuracy is about 2% higher, especially in the early time, being 5% higher for 10%-length data. Take sepsis diagnosis as an example, compared with the best baseline, our method improves the accuracy by 1.4% on average, 2.2% in the early 50% time stage when the key features are unobvious. Each hour of delayed treatment increases sepsis mortality by 4-8%. With the same accuracy, we can predict 0.972 h in advance.

Dataset	Method	10%	20%	30%	40%	50%
SEPSIS	LSTM	0.576±0.063	0.629±0.035	0.735±0.064	0.736±0.064	0.745±0.056
	SR	0.626±0.035	0.659±0.015	0.768±0.013	0.791±0.026	0.803±0.018
	ECEC	0.623±0.024	0.669±0.019	0.761±0.016	0.793±0.016	0.811±0.015
	EWC	0.671±0.027	0.733±0.023	0.799±0.015	0.827±0.036	0.832±0.028
	GEM	0.670±0.026	0.730±0.024	0.802±0.018	0.826±0.033	0.834±0.026
	CLEAR	0.680±0.028	0.732±0.024	0.801±0.015	0.825±0.035	0.833±0.025
	CLOPS	0.684±0.025	0.733±0.025	0.802±0.017	0.824±0.036	0.830±0.023
	RU	**0.690±0.032**	**0.734±0.038**	**0.812±0.022**	**0.828±0.036**	**0.835±0.024**
COVID-19	LSTM	0.605±0.044	0.701±0.033	0.793±0.022	0.833±0.015	0.844±0.013
	SR	0.636±0.014	0.730±0.024	0.810±0.013	0.867±0.016	0.901±0.013
	ECEC	0.639±0.013	0.732±0.028	0.829±0.013	0.870±0.016	0.901±0.026
	EWC	0.703±0.022	0.769±0.015	0.870±0.014	0.888±0.028	0.915±0.017
	GEM	0.699±0.025	0.779±0.017	0.871±0.015	0.885±0.022	0.914±0.019
	CLEAR	0.710±0.013	0.785±0.019	0.870±0.016	0.879±0.016	0.916±0.024
	CLOPS	0.709±0.017	0.775±0.013	0.869±0.012	0.900±0.017	0.918±0.026
	RU	**0.712±0.021**	**0.790±0.023**	**0.872±0.013**	**0.901±0.022**	**0.919±0.016**
MIMIC-III	LSTM	0.600±0.042	0.651±0.030	0.683±0.024	0.701±0.019	0.764±0.013
	SR	0.656±0.012	0.682±0.019	0.720±0.013	0.750±0.012	0.791±0.012
	ECEC	0.650±0.011	0.679±0.021	0.715±0.014	0.748±0.013	0.783±0.011
	EWC	0.652±0.009	0.674±0.018	0.716±0.012	0.747±0.014	0.785±0.012
	GEM	0.653±0.012	0.675±0.014	0.714±0.012	0.745±0.012	0.786±0.010
	CLEAR	0.658±0.011	0.682±0.009	0.722±0.009	0.751±0.013	0.793±0.010
	CLOPS	0.659±0.010	0.681±0.010	0.724±0.010	0.753±0.012	0.794±0.008
	RU	**0.662±0.012**	**0.689±0.013**	**0.759±0.011**	**0.758±0.012**	**0.801±0.010**
UCR-EQ	LSTM	0.695±0.044	0.711±0.038	0.803±0.024	0.843±0.019	0.854±0.017
	SR	0.700±0.015	0.736±0.014	0.830±0.016	0.863±0.015	0.871±0.024
	ECEC	0.703±0.013	0.738±0.018	0.828±0.017	0.865±0.014	0.873±0.026
	EWC	0.724±0.015	0.768±0.018	0.848±0.014	0.874±0.016	0.883±0.025
	GEM	0.723±0.014	0.767±0.017	0.850±0.015	0.876±0.016	0.890±0.024
	CLEAR	0.729±0.015	0.770±0.015	0.852±0.019	0.880±0.013	0.899±0.026
	CLOPS	0.728±0.016	0.773±0.016	0.855±0.015	0.878±0.016	0.896±0.028
	RU	**0.730±0.022**	**0.774±0.023**	**0.856±0.015**	**0.882±0.022**	**0.900±.0017**
USHCN	LSTM	0.682±0.014	0.700±0.028	0.721±0.013	0.745±0.028	0.784±0.023
	SR	0.702±0.014	0.730±0.022	0.745±0.016	0.761±0.023	0.809±0.024
	ECEC	0.707±0.017	0.736±0.024	0.748±0.015	0.760±0.025	0.806±0.025
	EWC	0.727±0.018	0.736±0.025	0.768±0.017	0.798±0.024	0.805±0.022
	GEM	0.720±0.019	0.728±0.026	0.772±0.015	0.781±0.023	0.801±0.026
	CLEAR	0.728±0.016	0.738±0.025	0.773±0.018	0.784±0.024	0.802±0.027
	CLOPS	0.728±0.012	0.740±0.024	0.769±0.019	0.781±0.025	0.800±0.024
	RU	**0.730±0.018**	**0.742±0.017**	**0.775±0.016**	**0.791±0.021**	**0.810±.0133**
ACTIV	LSTM	0.701±0.022	0.720±0.019	0.743±0.020	0.751±0.021	0.766±0.017
	SR	0.718±0.020	0.723±0.014	0.739±0.016	0.753±0.015	0.768±0.016
	ECEC	0.720±0.010	0.722±0.012	0.742±0.012	0.752±0.010	0.769±0.010
	EWC	0.721±0.012	0.726±0.013	0.744±0.012	0.754±0.009	0.770±0.008
	GEM	0.721±0.011	0.727±0.010	0.745±0.015	0.757±0.007	0.771±0.006
	CLEAR	0.726±0.012	0.730±0.013	0.744±0.014	0.758±0.011	0.775±0.011
	CLOPS	0.724±0.012	0.728±0.007	0.747±0.014	0.760±0.013	0.776±0.010
	RU	**0.729±0.010**	**0.732±0.013**	**0.752±0.014**	**0.763±0.011**	**0.780±0.010**

Table s3
Classification Accuracy (AUC-ROC↑) at the Last 5 Time Steps
*k% means the current classification time is k% of the total time of the full-length time series; Bold font indicates the highest accuracy.
Our method is significantly better than baselines by using Bonferroni-Dunn test. RU can classify more accurately at every time. The average accuracy is about 2% higher, especially in the early time, being 5% higher for 10%-length data. Take sepsis diagnosis as an example, compared with the best baseline, our method improves the accuracy by 1.4% on average, 2.2% in the early 50% time stage when the key features are unobvious. Each hour of delayed treatment increases sepsis mortality by 4-8%. With the same accuracy, we can predict 0.972 h in advance.

Dataset	Method	60%	70%	80%	90%	100%
SEPSIS	LSTM	0.748±0.043	0.773±0.032	0.795±0.027	0.813±0.025	0.827±0.039
	SR	0.827±0.037	0.835±0.013	0.845±0.014	0.859±0.022	0.866±0.023
	ECEC	0.815±0.014	0.827±0.016	0.849±0.016	0.859±0.017	0.863±0.014
	EWC	0.838±0.024	0.842±0.030	0.848±0.017	0.850±0.014	0.854±0.016
	GEM	0.836±0.028	0.841±0.034	0.849±0.014	0.851±0.016	0.853±0.012
	CLEAR	0.839±0.028	0.842±0.031	0.847±0.010	0.850±0.019	0.848±0.016
	CLOPS	0.838±0.026	0.842±0.030	0.850±0.017	0.853±0.010	0.857±0.018
	RU	**0.842±0.034**	**0.852±0.023**	**0.857±0.012**	**0.866±0.014**	**0.872±0.012**
COVID-19	LSTM	0.888±0.013	0.918±0.033	0.925±0.014	0.939±0.005	0.944±0.015
	SR	0.900±0.018	0.935±0.010	0.946±0.006	0.952±0.017	0.962±0.005
	ECEC	0.904±0.014	0.937±0.008	0.948±0.015	0.952±0.008	0.963±0.017
	EWC	0.923±0.014	0.935±0.007	0.940±0.013	0.950±0.013	0.954±0.008
	GEM	0.924±0.018	0.936±0.009	0.939±0.010	0.949±0.017	0.953±0.005
	CLEAR	0.926±0.014	0.933±0.011	0.941±0.007	0.948±0.009	0.952±0.008
	CLOPS	0.925±0.015	0.935±0.013	0.940±0.007	0.947±0.006	0.954±0.006
	RU	**0.927±0.006**	**0.955±0.008**	**0.960±0.011**	**0.963±0.009**	**0.967±0.008**
MIMIC-III	LSTM	0.780±0.021	0.791±0.024	0.825±0.020	0.820±0.020	0.849±0.021
	SR	0.792±0.017	0.795±0.016	0.805±0.017	0.821±0.015	0.856±0.013
	ECEC	0.792±0.013	0.791±0.012	0.810±0.012	0.820±0.013	0.861±0.012
	EWC	0.793±0.013	0.795±0.013	0.812±0.010	0.822±0.012	0.860±0.013
	GEM	0.791±0.013	0.794±0.013	0.813±0.012	0.820±0.013	0.859±0.012
	CLEAR	0.797±0.012	0.801±0.012	0.814±0.011	0.823±0.011	0.861±0.013
	CLOPS	0.799±0.010	0.803±0.011	0.813±0.012	0.821±0.009	0.860±0.010
	RU	**0.809±0.010**	**0.806±0.012**	**0.820±0.007**	**0.824±0.010**	**0.863±0.009**
UCR-EQ	LSTM	0.874±0.012	0.913±0.034	0.909±0.014	0.919±0.008	0.924±0.012
	SR	0.888±0.017	0.924±0.010	0.928±0.105	0.936±0.103	0.941±0.104
	ECEC	0.890±0.015	0.923±0.013	0.929±0.107	0.936±0.006	0.940±0.009
	EWC	0.895±0.014	0.910±0.017	0.923±0.102	0.930±0.005	0.933±0.003
	GEM	0.900±0.015	0.920±0.015	0.929±0.008	0.935±0.003	0.934±0.004
	CLEAR	0.904±0.012	0.918±0.019	0.923±0.004	0.928±0.007	0.932±0.005
	CLOPS	0.902±0.015	0.915±0.010	0.917±0.006	0.921±0.009	0.925±0.005
	RU	**0.906±0.005**	**0.928±0.007**	**0.933±0.010**	**0.940±0.005**	**0.946±0.003**
USHCN	LSTM	0.820±0.015	0.837±0.024	0.852±0.014	0.869±0.025	0.891±0.002
	SR	0.836±0.016	0.886±0.023	0.902±0.013	0.921±0.026	0.933±0.009
	ECEC	0.837±0.016	0.887±0.027	0.906±0.017	0.920±0.028	0.931±0.009
	EWC	0.834±0.016	0.867±0.026	0.896±0.017	0.906±0.020	0.926±0.007
	GEM	0.838±0.013	0.868±0.029	0.899±0.010	0.910±0.021	0.928±0.005
	CLEAR	0.837±0.010	0.867±0.023	0.879±0.012	0.899±0.027	0.921±0.004
	CLOPS	0.835±0.016	0.861±0.024	0.877±0.011	0.895±0.016	0.919±0.013
	RU	**0.841±0.012**	**0.898±0.022**	**0.910±0.015**	**0.928±0.013**	**0.939±0.013**
ACTIV	LSTM	0.790±0.021	0.822±0.021	0.835±0.022	0.866±0.020	0.879±0.020
	SR	0.821±0.011	0.834±0.013	0.846±0.009	0.870±0.012	0.877±0.012
	ECEC	0.822±0.010	0.830±0.010	0.842±0.010	0.872±0.011	**0.886±0.009**
	EWC	0.821±0.011	0.832±0.014	0.844±0.011	0.874±0.010	0.877±0.011
	GEM	**0.825±0.007**	0.831±0.010	0.842±0.012	0.873±0.010	0.878±0.008
	CLEAR	0.820±0.010	0.829±0.006	0.843±0.008	0.870±0.009	**0.886±0.006**
	CLOPS	**0.825±0.009**	0.833±0.006	0.844±0.009	**0.875±0.006**	0.885±0.006
	RU	**0.825±0.010**	**0.834±0.009**	**0.846±0.007**	0.874±0.008	0.885±0.010

Table s4
Average Classification Accuracy↑ (Rank↓) of Methods on UCR Time Series Classification Archive

	Class	LSTM	SR	ECEC	EWC	GRM	CLEAR	CLOPS	RU
Coffee	2	0.82(8)	0.83(6)	0.83(6)	0.85(4)	0.85(4)	0.89(2)	0.88(3)	0.91(1)
Gun Point	2	0.88(8)	0.89(7)	0.91(5)	0.90(6)	0.93(1)	0.93(1)	0.93(1)	0.93(1)
MoteStrain	2	0.79(8)	0.82(6)	0.81(7)	0.83(1)	0.83(1)	0.82(4)	0.82(3)	0.82(3)
SonyAIBOrobotsurface1	2	0.80(8)	0.82(4)	0.82(4)	0.82(4)	0.82(4)	0.85(1)	0.84(3)	0.85(1)
SonyAIBOrobotsurface2	2	0.78(6)	0.78(6)	0.78(6)	0.81(1)	0.79(5)	0.80(2)	0.80(2)	0.80(2)
Wafer	2	0.94(3)	0.94(3)	0.93(8)	0.95(1)	0.95(1)	0.94(3)	0.94(3)	0.94(3)
Lightning2	2	0.68(8)	0.69(7)	0.71(5)	0.70(6)	0.73(1)	0.73(1)	0.72(3)	0.72(3)
Yoga	2	0.78(6)	0.78(6)	0.78(6)	0.80(1)	0.79(3)	0.79(3)	0.79(3)	0.80(1)
CBF	3	0.73(8)	0.74(7)	0.75(3)	0.77(1)	0.76(2)	0.75(3)	0.74(6)	0.75(3)
ChlorineConcentration	3	0.70(8)	0.71(6)	0.72(4)	0.72(4)	0.71(6)	0.73(1)	0.73(1)	0.73(1)
FaceFour	4	0.75(2)	0.75(2)	0.75(2)	0.74(7)	0.74(7)	0.76(1)	0.75(2)	0.75(2)
Oliveoil	4	0.90(6)	0.89(8)	0.90(6)	0.91(5)	0.92(3)	0.92(3)	0.93(1)	0.93(1)
Beef	5	0.69(4)	0.69(4)	0.68(8)	0.69(4)	0.70(2)	0.70(2)	0.71(1)	0.69(4)
Symbols	6	0.70(6)	0.69(8)	0.70(6)	0.71(1)	0.71(1)	0.71(1)	0.71(1)	0.71(1)
SyntheticControl	6	0.84(2)	0.84(2)	0.84(2)	0.84(2)	0.83(8)	0.84(2)	0.84(2)	0.84(1)
Average Rank		6.1	5.5	5.2	3.2	3.3	2.0	2.3	**1.9**

Table s5
Continual Learning Performance (BWT↑) of Methods
[1]LSTM, SR and ECEC are not listed as they have no CL strategy. It's pointless to use BWT and FBT to evaluate them.
Our strategy can alleviate the catastrophic forgetting and promote the overall performance by sub-distribution. RU has the best performance on the early time series, showing the ability of LM to alleviate catastrophic forgetting. RU has the highest BWT, meaning it has the lowest negative influence that learning the new tasks has on the old tasks. RU has the highest FWT, meaning it has the highest positive influence that learning the former data distributions has on the task, especially for Sepsis and COVID-19 datasets.

Method[1] / Dataset	OSFW	ORGFW	GEM	CLOPS	RU
SEPSIS	−0.070	−0.066	+0.017	+0.006	**+0.032**
COVID-19	−0.026	−0.015	+0.012	+0.004	**+0.021**
MIMIC-III	−0.153	−0.161	+0.104	+0.043	**+0.125**
UCR-EQ	+0.109	+0.112	+0.123	+0.149	**+0.162**
USHCN	+0.065	+0.072	+0.098	+0.082	**+0.124**
ACTIV	−0.128	−0.107	+0.048	+0.039	**+0.075**
UCR	+0.072	+0.86	+0.101	+0.109	**+0.114**

Table s6
Continual Learning Performance (FWT↑) of Baselines
[1]LSTM, SR and ECEC are not listed as they have no CL strategy. It's pointless to use BWT and FBT to evaluate them.

Method / Dataset	OSFW	ORGFW	GEM	CLOPS	RU
SEPSIS	+0.323	+0.309	+0.265	+0.237	**+0.415**
COVID-19	+0.469	+0.478	+0.421	+0.289	**+0.498**
MIMIC-III	+0.197	+0.217	+0.287	+0.246	**+0.364**
UCR-EQ	+0.312	+0.363	+0.343	+0.384	**+0.399**
USHCN	+0.300	+0.316	+0.322	+0.301	**+0.348**
ACTIV	+0.134	+0.167	+0.184	+0.192	**+0.201**
UCR	+0.210	+0.226	+0.240	+0.253	**+0.259**

Table s7
AUC-ROC↑, BWT↑, FWT↑ and Gradient Fluctuation R↓ of Ablation of RU
Both LM and PM strategies contribute to model performance. If we remove two strategies respectively, the model accuracy will decline, the relation between tasks will become worse, the model instability will increase.

Dataset	Method[1]	Time 4	Time 6	Time 8	Time 10	BWT	FWT	R
SEPSIS	w/o PM	0.750±.03	0.796±.02	0.812±.02	0.830±.02	-0.102	+0.165	0.401
	w/o LM	0.743±.03	0.790±.02	0.801±.01	0.825±.02	-0.111	+0.160	0.400
	RU	**0.828±.01**	**0.842±.01**	**0.857±.01**	**0.872±.01**	**+0.032**	**+0.415**	**0.247**
COVID-19	w/o PM	0.879±.02	0.924±.02	0.931±.01	0.948±.01	-0.058	+0.195	0.328
	w/o LM	0.870±.01	0.914±.01	0.925±.00	0.935±.01	-0.088	+0.190	0.306
	RU	**0.901±.00**	**0.927±.01**	**0.960±.00**	**0.967±.00**	**+0.021**	**+0.498**	**0.248**
MIMIC-III	w/o PM	0.746±.01	0.760±.01	0.805±.01	0.828±.01	+0.053	+0.272	0.344
	w/o LM	0.755±.01	0.770±.01	0.812±.01	0.829±.01	+0.103	+0.312	0.338
	RU	**0.758±.01**	**0.806±.00**	**0.820±.01**	**0.863±.00**	**+0.125**	**+0.364**	**0.333**
UCR-EQ	w/o PM	0.776±.01	0.812±.02	0.838±.00	0.885±.01	+0.053	+0.246	0.277
	w/o LM	0.775±.01	0.810±.02	0.840±.00	0.886±.01	+0.080	+0.338	0.249
	RU	**0.882±.01**	**0.906±.00**	**0.933±.01**	**0.946±.00**	**+0.162**	**+0.399**	**0.246**
USHCN	w/o PM	0.706±.01	0.808±.02	0.849±.00	0.905±.01	+0.053	+0.289	0.209
	w/o LM	0.771±.01	0.812±.02	0.850±.00	0.910±.01	+0.080	+0.294	0.212
	RU	**0.791±.01**	**0.841±.00**	**0.910±.01**	**0.939±.00**	**+0.124**	**+0.348**	**0.257**
ACTIV	w/o PM	0.738±.01	0.808±.02	0.810±.01	0.827±.01	+0.033	+0.120	0.350
	w/o LM	0.723±.02	0.812±.01	0.826±.01	0.875±.01	+0.032	+0.123	0.347
	RU	**0.763±.01**	**0.825±.01**	**0.846±.01**	**0.885±.01**	**+0.075**	**+0.261**	**0.323**
UCR	w/o PM	0.752	0.786	0.799	0.845	+0.104	+0.231	0.212
	w/o LM	0.753	0.793	0.810	0.844	+0.103	+0.220	0.204
	RU	**0.790**	**0.815**	**0.824**	**0.859**	**+0.114**	**+0.259**	**0.153**

Table s8
Performance (AUC-ROC↑, BWT↑, FWT↑) of RU under Different Class Number and Training Orders of MIMIC-III Dataset
Class number and training order will influence the result: Fewer classes lead to better performance of RU; A sound training order can improve the model performance. If we increase the diagnosis number in MIMIC-III, the accuracy will decrease. Besides, No matter what order is adopted, RU has stable accuracy. It shows the possibility of global optimization potential of PM in RU. Based on the Gaussian distribution $\mathcal{N}(\mu, \sigma^2)$ of each class and the similarity of class i and class j $S(i,j) = 1 - \sqrt{1 - \sqrt{\frac{2\sigma_i\sigma_j}{\sigma i^2 + \sigma j^2}} e^{-\frac{1}{4}\frac{(\mu_i - \mu_j)^2}{\sigma i^2 + \sigma j^2}}}$, we can obtain a new class order in Figure s6.

	**2 Classes**	4 Classes	6 Classes	8 Classes	Random	ICD-9	Similarity
AUC-ROC	**0.859±.01**	0.831±.01	0.816±.01	0.797±.01	0.832±.01	0.830±.01	**0.845±.01**
BWT	**+0.153**	+0.142	+0.139	+0.135	+0.125	+0.122	**+0.133**
FWT	**+0.398**	+0.384	+0.379	+0.365	+0.364	+0.358	**+0.367**

Table s9
Performance (AUC-ROC↑, BWT↑, FWT↑) of RU under Different Training Order in Different Time Stages of MIMIC-III Dataset

Order	30%	60%	90%	BWT	FWT
Random	0.757±.01	0.788±.01	0.832±.01	+0.125	+0.364
ICD-9	0.759±.01	0.783±.01	0.830±.01	+0.122	+0.358
Similarity	**0.762±.01**	**0.796±.00**	**0.845±.01**	**+0.133**	**+0.367**

Table s10
COVID-19 Classification Accuracy with Non-uniform Training Sets and Validation Sets
↓ means the accuracy is greatly reduced. Bold font indicates the smallest decline in accuracy.

Subset	LSTM	SR	ECEC	EWC
Male	0.955±0.013	0.968±0.014	0.969±0.016	0.965±0.012
Female	0.924±0.013	0.945±0.004	0.947±0.015	0.939±0.018
Age 55-	0.954±0.013	0.965±0.014	0.967±0.015	0.967±0.013
Age 55+	0.923±0.014	0.941±0.007	0.943±0.018	0.931±0.008↓
Test	0.950±0.011	0.964±0.013	0.968±0.015	0.966±0.012
Valid.	0.944±0.014	0.962±0.006	0.963±0.014	0.954±0.003

Subset	GEM	CLEAR	CLOPS	RU
Male	0.965±0.004	0.978±0.009	0.978±0.014	0.971±0.010
Female	0.938±0.003	0.919±0.008↓	0.921±0.009↓	**0.947±0.002**
Age 55-	0.964±0.009	0.977±0.008	0.979±0.012	0.972±0.010
Age 55+	0.923±0.040↓	0.902±0.006↓	0.914±0.007↓	**0.945±0.006**
Test	0.962±0.006	0.979±0.009	0.978±0.010	0.970±0.007
Valid.	0.953±0.005	0.952±0.009↓	0.954±0.004↓	**0.967±0.006**

Table s11
Classification Accuracy (AUC-ROC↑) of RU under Training Sets with Different Data Size
k% means the volume of sub dataset is k% of the corresponding original dataset; Bold font indicates the highest accuracy; *means that the accuracy of RU is higher 2% than this method.

Dataset	Method	20%	40%	60%	80%	100%
SEPSIS	LSTM	0.658*	0.669*	0.691*	0.733*	0.747
	SR	0.682	0.700	0.725*	0.759*	0.768
	ECEC	0.679*	0.702	0.719*	0.755*	0.770
	EWC	0.685	0.708	0.729*	0.768*	0.772*
	GEM	0.693	0.704	0.740*	0.771*	0.781*
	CLEAR	0.687	0.705	0.741	0.776	0.789
	CLOPS	0.698	0.710	0.745	0.779	0.783*
	RU	**0.701**	**0.712**	**0.760**	**0.794**	**0.803**
COVID-19	LSTM	0.713*	0.730*	0.765*	0.819*	0.834*
	SR	0.751*	0.767*	0.806	0.822*	0.842*
	ECEC	0.755*	0.770*	0.796*	0.829*	0.856*
	EWC	0.763	0.785	0.794*	0.835*	0.849*
	GEM	0.769	0.772*	0.793*	0.849	0.856*
	CLEAR	0.776	0.791	0.810	0.856	0.866*
	CLOPS	0.775	0.789	0.809	0.848	0.874
	RU	**0.781**	**0.800**	**0.821**	**0.863**	**0.888**
UCR-EQ	LSTM	0.724*	0.765*	0.804*	0.809*	0.813*
	SR	0.758*	0.784*	0.828*	0.813*	0.831*
	ECEC	0.790	0.770*	0.815*	0.827*	0.838*
	EWC	0.785	0.791*	0.833*	0.855*	0.862*
	GEM	0.780	0.775*	0.840*	0.857*	0.863*
	CLEAR	0.784	0.808	0.859	0.864*	0.870*
	CLOPS	0.792	0.809	0.864	0.871	0.875*
	RU	**0.797**	**0.817**	**0.872**	**0.886**	**0.896**
USHCN	LSTM	0.701*	0.730*	0.732*	0.760*	0.763*
	SR	0.731*	0.769*	0.782*	0.801*	0.805*
	ECEC	0.747*	0.774	0.800*	0.807*	0.816*
	EWC	0.739*	0.768*	0.810	0.817	0.826*
	GEM	0.737*	0.772	0.809	0.811*	0.818*
	CLEAR	0.757	0.780	0.812	0.819	0.823*
	CLOPS	0.775	0.785	0.817	0.825	0.839
	RU	**0.776**	**0.790**	**0.821**	**0.835**	**0.843**
ACTIV	LSTM	0.732*	0.780	0.809	0.814	0.825
	SR	0.743*	0.783	0.814	0.815	0.823
	ECEC	0.745*	0.786	0.817	0.818	0.827
	EWC	0.742*	0.785	0.816	0.819	0.828
	GEM	0.748*	0.782	0.813	0.817	0.829
	CLEAR	0.752	0.787	0.816	0.823	0.832
	CLOPS	0.757	0.789	0.819	0.820	0.830
	RU	**0.770**	**0.795**	**0.820**	**0.830**	**0.834**
UCR	LSTM	0.663*	0.714*	0.744*	0.796*	0.813
	SR	0.672*	0.717*	0.754*	0.806	0.820
	ECEC	0.670*	0.721	0.755*	0.810	0.819
	EWC	0.676*	0.722	0.763*	0.814	0.821
	GEM	0.675*	0.721	0.765*	0.812	0.820
	CLEAR	0.681*	0.721*	0.770	0.813	0.822
	CLOPS	0.687	0.725	0.771	0.815	0.820
	RU	**0.705**	**0.741**	**0.788**	**0.819**	**0.824**

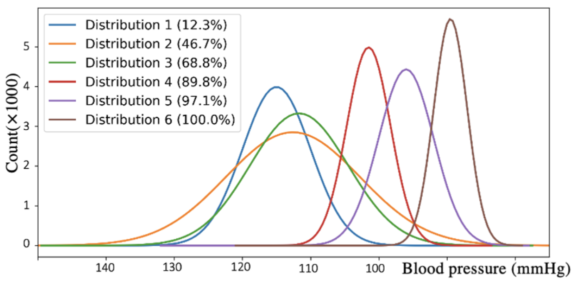

(a) Multi-distribution in SEPSIS Dataset

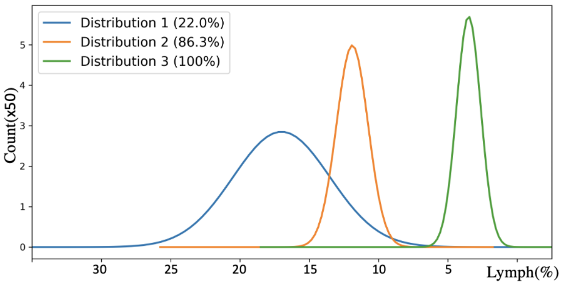

(b) Multi-distribution in COVID-19 Dataset

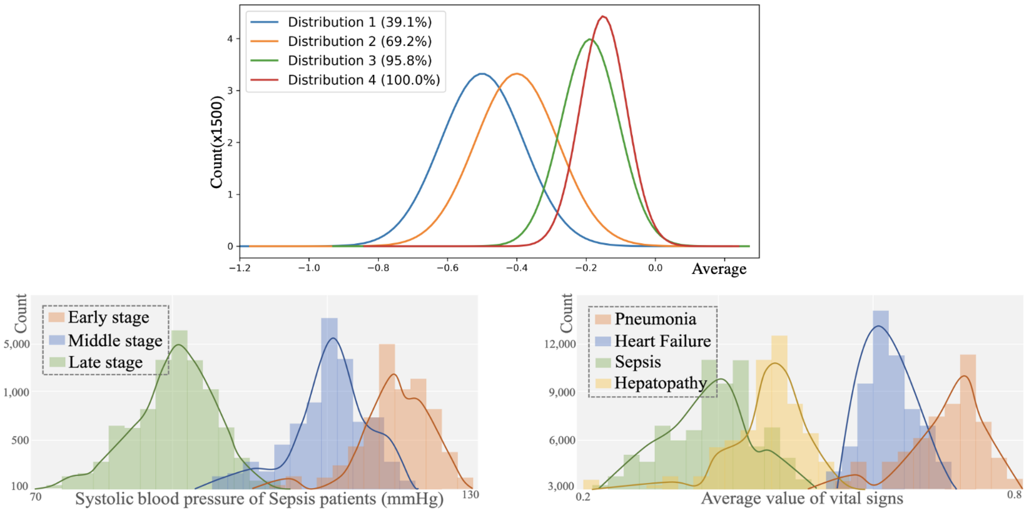

(c) Multi-distribution in MIMIC-III Dataset

Figure s3: Multi-distribution in Medical Datasets
Before discussing the method performance, we show the basic scenario of CCTS– multiple distributions. The data in different time stages have distinct statistical characteristics and finally form multi-distribution. The fundamental goal of the following experiment is to model them.
Sub-figure (a) shows six distributions in SEPSIS Dataset, each of them belongs to a time stage. Corresponding to the four stages in Figure 3, distribution 1, 2, 3 belong to stage 1, distribution 4 belongs to stage 2, distribution 5 belongs to stage 3, and distribution 6 belongs to stage 4.

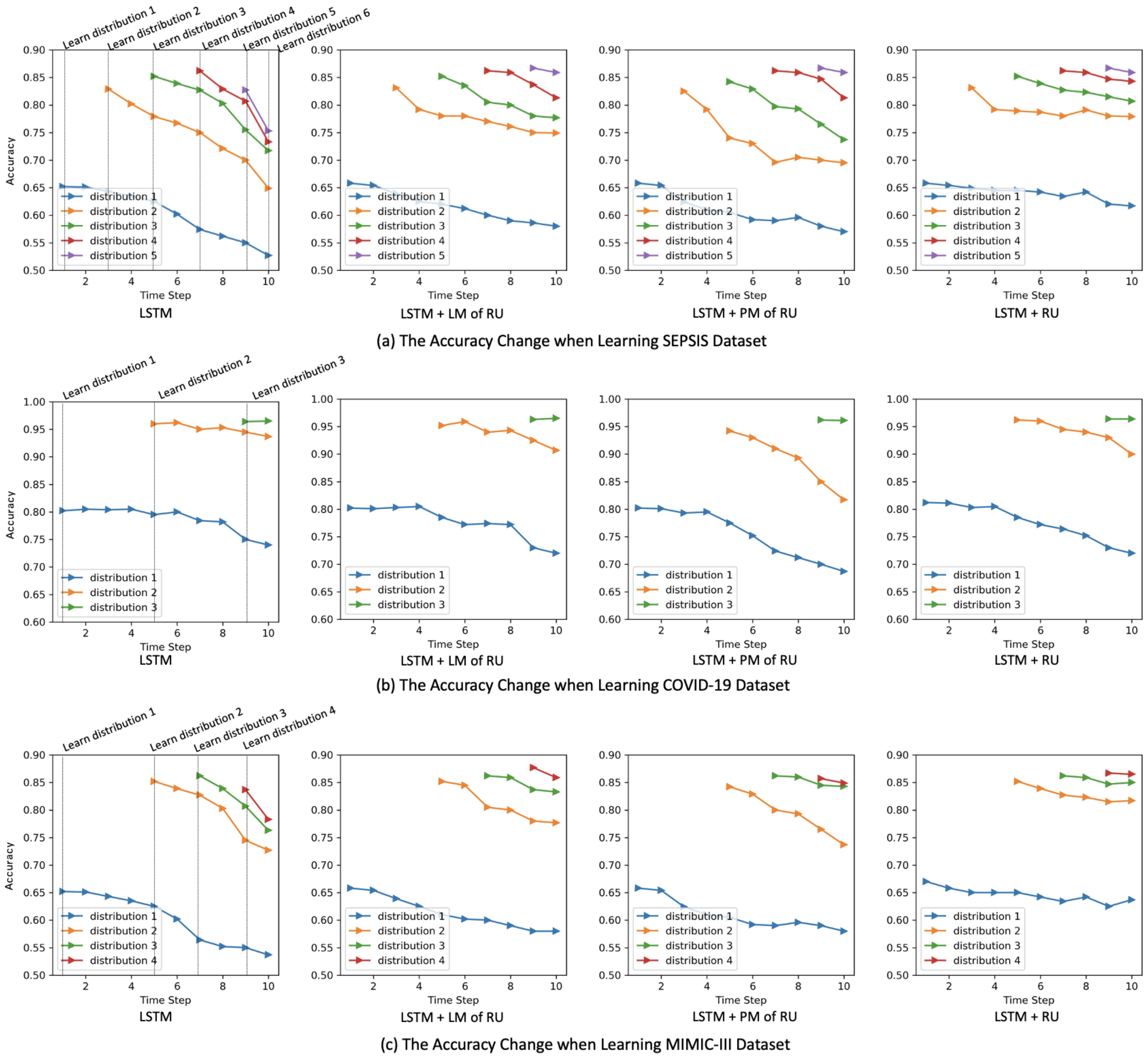

(a) The Accuracy Change when Learning SEPSIS Dataset

(b) The Accuracy Change when Learning COVID-19 Dataset

(c) The Accuracy Change when Learning MIMIC-III Dataset

Figure s4: The Accuracy Change in Continuous Classification
(1) When the model learns a new distribution, its performance on old distributions becomes worse (catastrophic forgetting); (2) After using the Limitation Mechanism (LM) of our method (`RU`), when learning a new distribution, the performance of the model on old distributions will not decline much; (3) After using the Promotion Mechanism (PM) of our method (`RU`), in some cases, e.g., at time step 8, learning the new distribution will help the model perform on the old distribution; (4) After using our method (`RU`, PM+LM), the accuracy of the model can be basically maintained in continuous learning

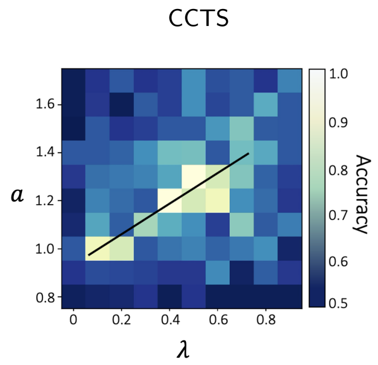

Figure s5: Parameter Test
Parameter λ decides the constraint degree on parameter update in Equation 5 of LM. In practice, we optimize it using the search method supplied by mature tools; Parameter a decides ρ in PM by $\rho_m = \eta_m = \frac{1}{(m+1)^a}$. ρ determines the correlation between current and previous gradients in Equation 12. We can optimize it using the search method supplied by mature tools.

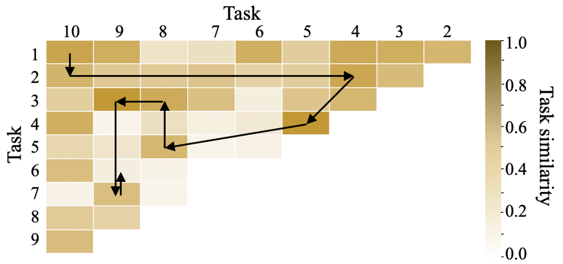

Figure s6: Task Similarity
In MIMIC-III dataset, the diagnoses with ICD-9 order are 1:HIV, 2:Brain Cancer, 3:Diabetes, 4:Hypertension, 5:Heart Failure, 6:Pneumonia, 7:Gastric Ulcer, 8:Hepatopathy, 9:Nephropathy, 10:SIRS. As we focus on eight diseases (3-10). The new similarity order is 10, 2, 4, 5, 8, 9, 7, 6.

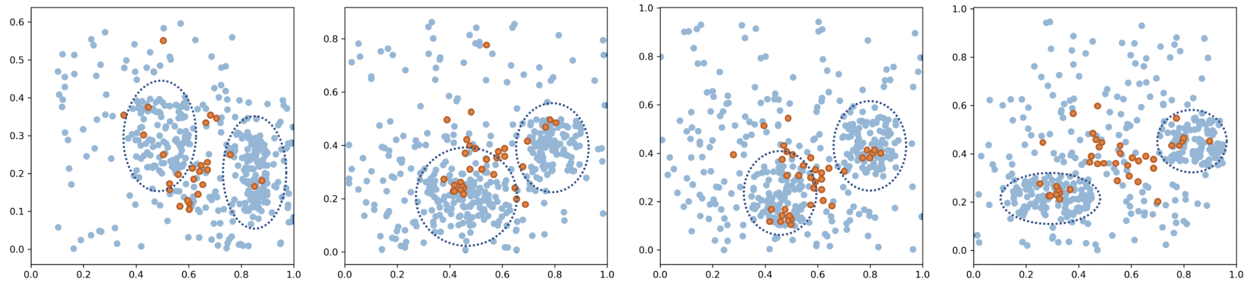

Figure s7: The Important Samples in Four SEPSIS Distribution Buffers when Using the Replay-based Method [5]
The four sub-figures are the sample representations of distribution 2,3,4,5 in Figure s3(a). Important samples should be in the circle, but the important samples found by this method are not completely correct.

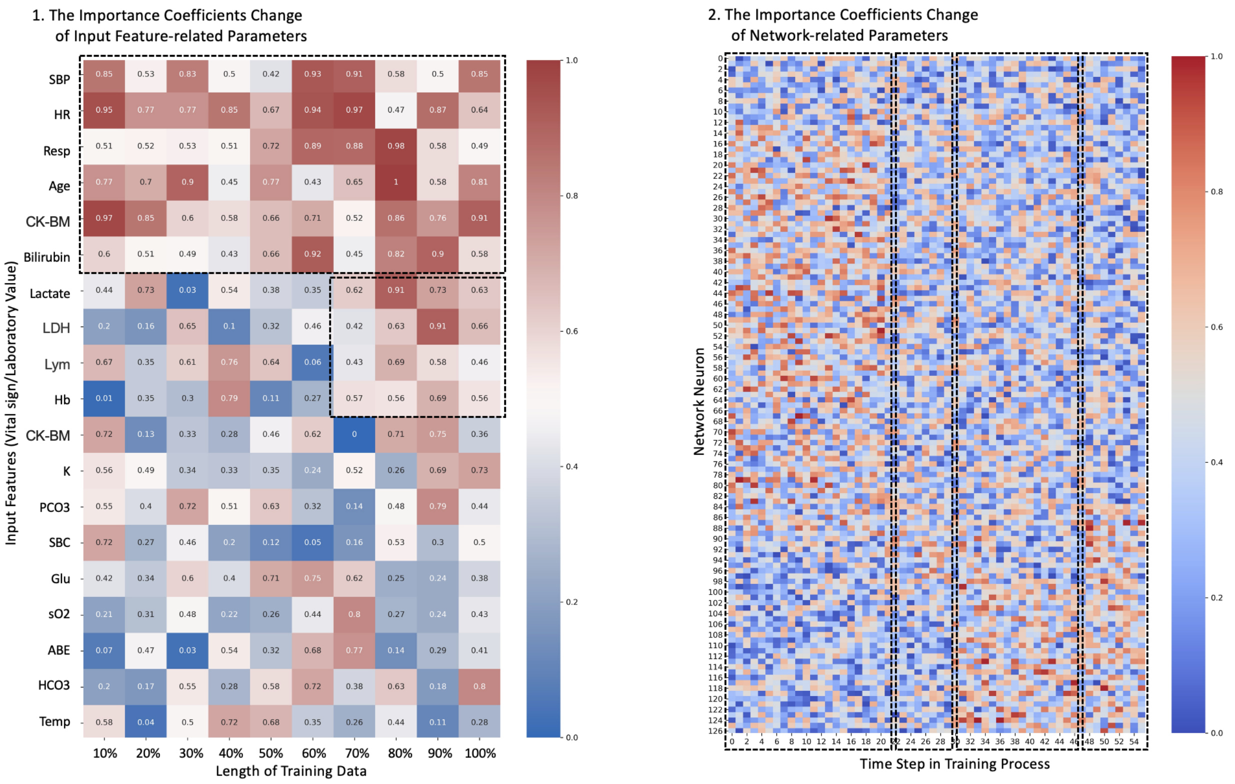

Figure s8: The Importance Change of Input Features and Network Parameters for MIMIC-III Eight Diseases Classification
Subfigure 1 shows the important input features for eight diseases classification: SBP, HR, Resp, Age, CK-BM, Bilirubin, Lactate, LDH, Lym, Hb, etc. Among them, SBP, HR, Resp, Age, CK-BM, and Bilirubin are important in the whole process; Lactate, LDH, Lym, and Hb are gradually important in the middle and late stages. This shows the necessity of continuous vital sign monitoring and blood routine examination during hospitalization, as well as the importance of detailed laboratory examination in later stages. Meanwhile, these important features are more related to circulatory system diseases, kidney disease, and liver disease, which also shows that the extracted MIMIC-III dataset has more records about these diseases. For example, 9,012 of the 19,993 records are about circulatory system disease/heart disease.
Subfigure 2 shows the change of importance coefficients of network-related parameters. It forms four stages in eight diseases classification. Under the constraint of `RU`, there is a big difference between the last three stages and the first stage. It implies that the patient's state has changed significantly since the second stage.
Since this task is not specific to a single disease, we can only call them task stages and important features, not disease stages and biomarkers.
