# Supplementary material for: Continuous diagnosis and prognosis by controlling the update process of deep neural networks
Source: Patterns (N Y). 2023 Feb 3;4(2):100687. doi: 10.1016/j.patter.2023.100687 (PMC9982300; doi:10.1016/j.patter.2023.100687)
Supplement: Document S2. Article plus supplemental information [file mmc2.pdf]

Article
Patterns

Continuous diagnosis and prognosis by controlling the update process of deep neural networks

Graphical abstract

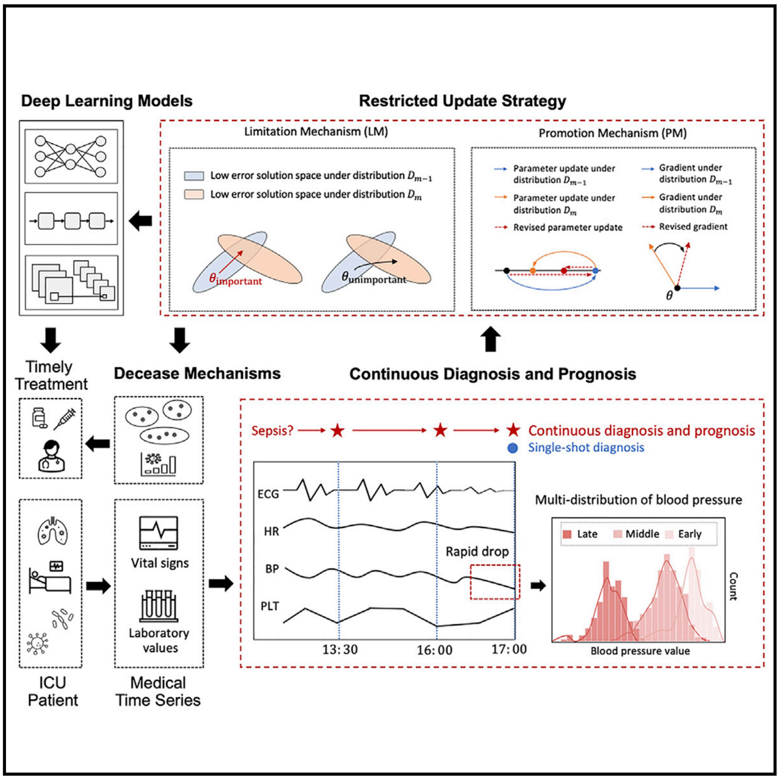

Highlights

- Restricted update strategy for training deep-learning models is presented
- Method can be used for continuous classification of time series
- Method is effective in continuous diagnosis and prognosis in the medical field
- Method can find disease stages and biomarkers

Authors

Chenxi Sun, Hongyan Li, Moxian Song, Derun Cai, Baofeng Zhang, Shenda Hong

Correspondence

hongshenda@pku.edu.cn (S.H.), leehy@pku.edu.cn (H.L.)

In brief

Continuous diagnosis and prognosis are crucial for patients in intensive care. They can provide more opportunities for timely treatment and rational resource allocation. Based on the task of continuous classification of time series, a restricted update strategy for deep neural networks is proposed. It can not only achieve continuous diagnosis and prognosis but can also detect stages and biomarkers of the disease, making the deep-learning model interpretable.

Sun et al., 2023, Patterns *4*, 100687
February 10, 2023 © 2023 The Author(s).
https://doi.org/10.1016/j.patter.2023.100687
CellPress

Patterns

CellPress
OPEN ACCESS

Article

Continuous diagnosis and prognosis by controlling the update process of deep neural networks

Chenxi Sun,[1,2] Hongyan Li,[1,2,*] Moxian Song,[1,2] Derun Cai,[1,2] Baofeng Zhang,[1,2] and Shenda Hong[3,4,5,*]
[1]Key Laboratory of Machine Perception (Ministry of Education), Peking University, Beijing 100871, China
[2]School of Intelligence Science and Technology, Peking University, Beijing 100871, China
[3]National Institute of Health Data Science, Peking University, Beijing 100191, China
[4]Institute of Medical Technology, Health Science Center of Peking University, Beijing 100191, China
[5]Lead contact
*Correspondence: hongshenda@pku.edu.cn (S.H.), leehy@pku.edu.cn (H.L.)
https://doi.org/10.1016/j.patter.2023.100687

THE BIGGER PICTURE Patients in intensive care require rapid and accurate diagnoses at multiple time points. The quality and speed of these diagnoses can have a major impact on the success of treatment regimes for life-threatening conditions like sepsis or severe COVID-19. There is hope that deep-learning techniques could be used to create computer-based systems that would provide continuous real-time diagnoses, but these techniques face special challenges when dealing with time series data. Here, we develop and describe a method for training deep neural networks for these kinds of challenging applications and show that they can increase accuracy and provide better interpretability to the outputs, making them easier to apply in a clinical setting. Methods such as this one may ultimately help improve automated diagnosis systems in crucial clinical settings and could have applications in other time-sensitive settings that require continuous classification.

1 2 **3** 4 5 **Development/Pre-production:** Data science output has been rolled out/validated across multiple domains/problems

SUMMARY

Continuous diagnosis and prognosis are essential for critical patients. They can provide more opportunities for timely treatment and rational allocation. Although deep-learning techniques have demonstrated superiority in many medical tasks, they frequently forget, overfit, and produce results too late when performing continuous diagnosis and prognosis. In this work, we summarize the four requirements; propose a concept, continuous classification of time series (CCTS); and design a training method for deep learning, restricted update strategy (RU). The RU outperforms all baselines and achieves average accuracies of 90%, 97%, and 85% on continuous sepsis prognosis, COVID-19 mortality prediction, and eight disease classifications, respectively. The RU can also endow deep learning with interpretability, exploring disease mechanisms through staging and biomarker discovery. We find four sepsis stages, three COVID-19 stages, and their respective biomarkers. Further, our approach is data and model agnostic. It can be applied to other diseases and even in other fields.

INTRODUCTION

Continuous diagnosis and prognosis are of great significance for timely, personalized treatment and rational allocation of medical resources. Especially in the intensive care unit (ICU), status perception and disease diagnosis are needed at any time as real-time diagnosis provides more opportunities for doctors to rescue lives. For example, sepsis is a life-threatening condition, causing more than half of ICU deaths.[1] Early detection and antibiotic treatment are critical for improving sepsis outcomes;[2,3] COVID-19 outbreaks have caused health concerns worldwide. In the case of a sudden outbreak of a new epidemic, continuous

This is an open access article under the CC BY license (http://creativecommons.org/licenses/by/4.0/).

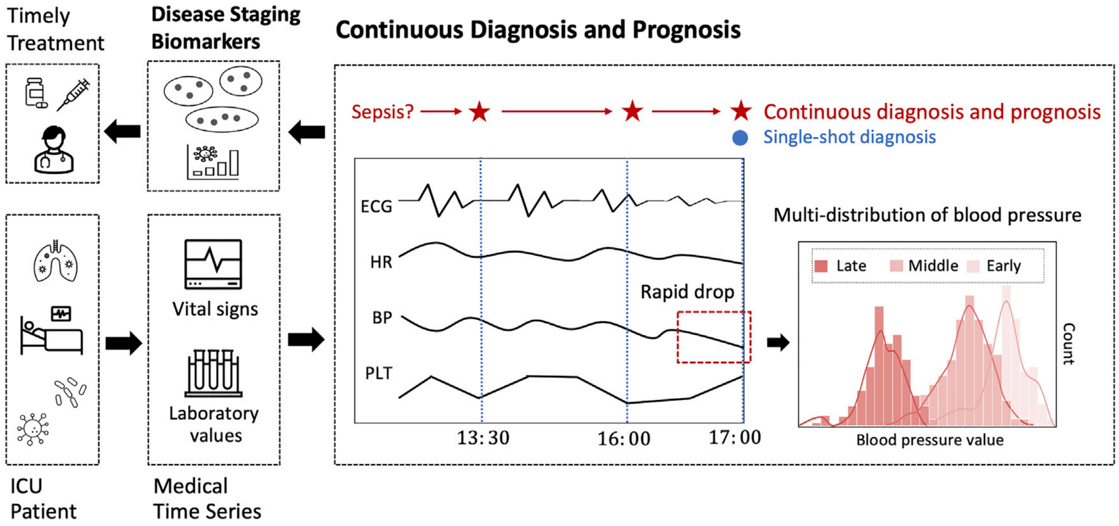

Figure 1. Continuous diagnosis and prognosis with disease staging

prognosis can help with personalized treatment and rational allocation of scarce resources.[4,5]

Different from the single-shot diagnosis, which is often made for the outpatient, the task of continuous diagnosis and prognosis emphasizes the multiple early diagnoses or prognoses for the inpatient at different stages over time. For example, in Figure 1, a patient in the ICU is monitored for vital signs in real time. Assuming that he will be in sepsis shock at 17:00, the common diagnostic system will give a warning when he is suffering or about to suffer from sepsis at about 17:00 (the single-shot diagnosis, blue dot). This is likely to miss the emergency treatment time for the acute disease, where each hour of delay has been associated with roughly a 4%–8% increase in sepsis mortality.[2] Thus, we require a continuous prognosis mode for sepsis (red stars), where we can predict the patient outcome 4 h early, 1 h early, etc., at 13:00, 16:00, etc. To meet this practical need, we summarize four requirements for the task of continuous diagnosis and prognosis.

Requirement 1: The ability to identify symptoms in different time stages before the disease onset

Single-shot diagnosis only needs learning the clinical manifestation, which is easy under the guidance of the gold standard. But continuous prognosis needs to learn the underlying symptoms of the disease in its early stages, which are usually not obvious in the clinic and cannot be judged by clinicians, and the symptoms are not only from a certain stage but from multiple stages before the onset, leading to diversity and hybridity.

Requirement 2: Potential for earlier diagnosis with guaranteed accuracy

Earlier diagnosis is necessary for many severe illnesses. Each hour of delayed treatment could cause a 4%–8% increase in sepsis mortality,[2] but basic questions about the limits of early detection remain unanswered. If one wants to pursue higher diagnostic accuracy, it will tend to predict late for clearer features. For example, a rapid drop in blood pressure (a major symptom of sepsis shock; the red dashed box in Figure 1) always occurs just before the shock.[1] But we expect the continuous mode to diagnose earlier and more accurately than the single-shot mode.

Requirement 3: Merits of explainability and disease staging

The 22nd article of the European Union's General Data Protection Regulation stipulates that a subject of algorithmic decisions has a right to meaningful explanation regarding said decisions.[6] As clinicians always justify a result using medical-domain knowledge familiar to them, the explainable methods will be more popular in practice.[7]

Meanwhile, continuous prognosis is accompanied by disease progression. Disease staging is important to understand disease mechanisms and implement targeted treatment. A clinically useful staging system stratifies patients by their baseline risk of an adverse outcome and their potential to respond to therapy. The best developed and most explicit approach has evolved in oncology,[8] but it is not clear for critical illnesses. Recently, the stratification for sepsis (sepsis, severe sepsis, and septic shock) has been questioned in the latest sepsis definition,[1] and there are no criteria for temporal septic stages.

Requirement 4: Function of offline and sustainable use

In many scenarios, especially in the ICU, we need to directly use the mature system without constant adjustment. A well-informed system can reduce the risk of misjudgment.[9] Further, in subsequent applications, when obtaining a batch of new data, such as new patients and new clinical observations, we hope to continue to use the current system instead of designing a new one because the data that have the old knowledge may still occur, while the new system cannot handle the old knowledge well.[10]

Nowadays, many studies have shown that deep learning (DL) methods are superior to medical gold standards and experienced doctors in some medical tasks such as outcome prediction and disease diagnosis.[11,12] Surprisingly, in these studies, sequential medical records, such as vital signs, multiple blood

samples, and serial medical imaging, provided more possibilities for DL models to implement diagnosis and prognosis. We uniformly name such sequential medical records as medical time series data. However, most DL-based models often give the single-shot diagnosis after learning the full-length medical time series but cannot prognose continuously. Although some subdisciplines study the mode of continuous learning, they cannot satisfy the above requirements at the same time (see supplemental related work and concepts).

The labels (mortality, morbidity, etc.) of real-world medical time series are usually determined at the final time. If the model simply learns the full-length time series, it can only give the single-shot result at the onset time. For continuous diagnosis and prognosis, the model needs to learn time series from different advanced stages: when the data change, the model performance needs to maintain. But most medical time series have evolved distribution. In Figure 1, the blood pressure varies among early, middle, and late stages, bringing a triple distribution. DL models lack the ability to learn all distributions simultaneously due to the premise of independent and identical distribution. As shown in Figure S4, learning the new knowledge may lead to forgetting old ones, and learning one distribution frequently may fall into local solutions with overfitting.

Meanwhile, interpretability is an elusive concept, and the artificial intelligence (AI) field holds no consensus regarding its definition.[13] Although some studies have proposed methods to explain the DL black-box model,[14] they are not fully interpretable, mostly explaining static models and depending on the actual scenario and task.[15] Thus, interpretation problems have not yet been fully overcome when using DL for medical applications.[16] When developing the method, we need to consider the possibility of it being explained and match it to the dynamic process of continuous diagnosis and prognosis.

To this end, we establish a training method for DL models, the restricted update strategy (RU) of neural network parameters. The `RU` can satisfy the above requirements: for requirement 1, it has the limitation mechanism (LM) to avoid catastrophic forgetting and overfitting; for requirement 2, it has the promotion mechanism (PM) to consolidate the knowledge of early distribution; for requirement 3, we define the importance coefficient of parameters to reveal the model development and achieve disease staging with typical biomarkers; and for requirement 4, we train the model by real-world datasets with separate training and test sets and test continual use. Experimental results show that the `RU` is more accurate than all baselines, achieving accuracies of 90%, 97%, and 85% on sepsis prognosis, COVID-19 mortality prediction, and eight disease diagnoses, respectively.

The major advantages of our study are 4-fold: (1) for continuous diagnosis and prognosis of time-sensitive illness, we design an `RU` for the DL model, which outperforms baselines. (2) The `RU` has the ability to interpret the update of the DL model and the change of medical time series through input indicators and parameter visualization. These side effects make our method attractive in medical applications where model interpretation and marker discovery are required. (3) We extend our method to connect the distribution change of vital signs with the parameter change of the DL model. We find typical disease biomarkers and stages of sepsis and COVID-19. (4) The `RU` is a data-agnostic, model-agnostic, and easy-to-use plug in. It can be used to train various types of DL models. The continuous prediction mode is needed in most time-sensitive applications, not just in medical tasks. As shown in Figure S2, we define this task as a distinctive concept—continuous classification of time series (`CCTS`), which is different from existing concepts and tasks as shown in Figure S3.

RESULTS

We test the `RU` and eight baselines on six datasets, using 5-fold cross-validation, expressed as the mean and SD (mean ± SD). The classification accuracy is evaluated by the area under the curve of the receiver operating characteristic (AUC-ROC; the higher the better) and the AUC confidence interval. The continuous classification performance is evaluated by backward transfer (BTW) and forward transfer (FWT; the higher the better). The statistical significance is evaluated by the Bonferroni-Dunn test. The learning stability is evaluated by the gradient fluctuation R (the lower the better). Eight baselines are long short-term memory (LSTM), stopping rule (SR),[17] effective confidence-based early classification (ECEC),[18] online stochastic recursive gradient-based Frank-Wolfe (ORGFW),[19] gradient episodic memory (GEM),[20] elastic weight consolidation (EWC),[21] continual learning with experience and replay (CLEAR),[22] and continual learning of physiological signals (CLOPS).[23] Three medical datasets are SEPSIS,[24] COVID-19,[25] and MIMIC-III.[26] Three additional datasets are United States Historical Climatology Network (USHCN),[27] University of California Riverside time series classification archive (UCR),[28] and human ACTIVity dataset (ACTIV)[29] (more experimental details are in the supplemental experimental procedures).

An RU to train DL models for continuous diagnosis and prognosis

For continuous medical diagnosis and prognosis, we focus on continuous sepsis prognosis, continuous COVID-19 mortality prediction, and continuous eight disease classification based on medical time series, including vital signs from various monitors, and continuous blood sample records during hospitalization. All used data are available: the SEPSIS dataset[24] has 30,336 ICU patient records with 2,359 diagnosed with sepsis from three separate hospital systems; the COVID-19 dataset[25] has 6,877 blood sample records of 485 COVID-19 patients from Tongji Hospital, Wuhan, China; and the MIMIC-III dataset[26] has 19,993 admission records from 7,537 patients, and we focus on 8 diseases.

A time series dataset $\mathcal{T} = \{X^n\}_{n=1}^N$ has N samples. Each sample $X = \{x_m\}_{m=1}^M$ has M observations with value x_m and time t_m. Multivariate time series can be described by $X = \{x_m^d\}_{d,m=1}^{D,M}$. d is the d-th dimension. DL models have achieved great success in modeling medical time series data,[30] especially recurrent neural networks (RNNs). However, the real-world time series is usually long and irregularly sampled. For example, critically ill patients are often hospitalized for several months; thus, records often have hundreds of observations. And due to the change in the patient's health status, the relevant measurement requirements are also changing, which may be several hours or days apart.[31] Thus, in order to model the long-term dependency and eliminate the impact of uneven time intervals, we implement time-aware

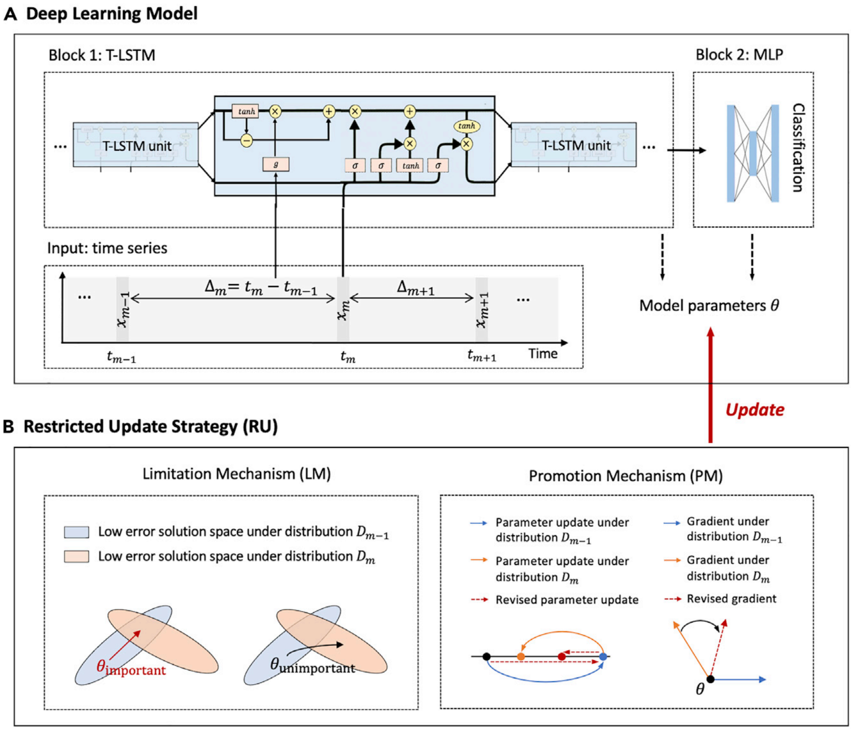

Figure 2. Restricted update strategy (RU) of neural networks for continuous classification of time series (CCTS)

LSTM (T-LSTM),[32] a variant of the RNN. As shown in Figure 2A, our DL architecture has two blocks: block 1 uses T-LSTM to model the input data and represent their hidden features with the consideration of time decay $\Delta_m = t_m - t_{m-1}$, and block 2 uses multilayer perceptron (MLP) to map features to the class.

After training T-LSTM by the full-length time series dataset, it can achieve average accuracies of 92%, 97%, and 88% on single-shot diagnosis for sepsis, COVID-19, and eight diseases, respectively. However, when applying it to continuous diagnosis and prognosis, the accuracy drops by more than 15%. Thus, we use the dataset consisting of subsequences of each sample in the original dataset $\mathcal{T}$ to train the model. The new dataset is $\mathcal{T}^* = \{X^n_{1:m}\}^{N,M}_{n,m=1}$. As the time series changes dynamically, datasets $\mathcal{T}^*_{m-1}, \mathcal{T}^*_m$ in time t_{m-1}, t_m may form different data distributions $\mathcal{D}_{m-1}, \mathcal{D}_m$. To learn such multidistribution, we propose an RU to train the DL model. As shown in Figure 2B, the RU has two mechanisms: LM and PM. The RU has the most stable gradient in the training process. As shown in Figures 3M–3O, it has the smallest R in any epoch, showing its restriction ability in the error back propagation of the DL model.

LM helps the DL model to learn multidistributed data, alleviating problems of catastrophic forgetting and overfitting. Due to the observation of many parameter configurations resulting in the same performance,[33] we could add a regular term to the loss to restrict model parameters. To this end, when learning a new distribution, LM constrains important parameters $\theta_{\text{important}}$ for the old distribution to stay close to their old values but changes unimportant parameters ($\theta_{\text{unimportant}}$) more. As shown in Figure 2B, when learning distribution $\mathcal{D}_m$, $\theta_{\text{important}}$ is limited to the low error space of distribution $\mathcal{D}_{m-1}$, while $\theta_{\text{unimportant}}$ can be updated to other spaces. In this way, different parameters can be arranged for different distributions. The importance of parameter θ is measured by the importance coefficients $\alpha(\theta)$. LM is implemented by using a loss in Equation 5. Its key feature is the use of the diagonal of the Fisher information matrix F to represent the importance coefficient α, allowing for the quantification of parameter importance.

PM helps the DL model to classify time series earlier in time-sensitive applications. It regards the process of a DL model learning early distributions and new distributions as the same continuous optimization problem with regret minimization. PM is projection free and estimates a stochastic recursive estimator to alleviate the complexity and training instability. As shown in Figure 2B, when learning distribution $\mathcal{D}_m$, PM changes the current gradient from an obtuse angle to an acute angle with the gradient on previous distribution $\mathcal{D}_{m-1}$ because when the new gradient and the old gradient are at an acute angle, the model performance on the old distribution will improve, or at least not decrease.[20] Most importantly, the promotion of learning old distributions has the potential for early classification. PM is implemented by a recursive estimator in Equation 12. It can fill the gap between the optimal regret bound and the low per-round computational cost, holding a nearly optimal regret bound $\tilde{O}(\sqrt{M})$, where M is the number of distributions.

Equations 5 and 12 serve as the main conduits for implementing the RU. It is clear that they have no bearing on the model's structure or tasks. Thus, the RU is a model-agnostic, task-agnostic, and easy-to-use plug in.

Finding 1: The continuous mode has more potential in medical diagnosis and prognosis than the single-shot mode

For the CCTS task, as shown in Figures 3G–3L, our method, RU, can classify more accurately at every time. It is significantly better than all 8 baselines in the Bonferroni-Dunn test ($\bar{r} = 3.5 > CD = 2.724$). The average accuracy is about 2% higher, especially in the early time, being 5% higher for 10%-length data. CCTS is important for time-sensitive applications, especially for acute and critical illnesses. Take sepsis diagnosis as an example: compared with the best baseline, the RU improves the accuracy by 1.4% on average and 2.2% in the early 50% time stage where the key features are unobvious. Each hour of delayed treatment increases mortality by 4%–8%.[2] With the same accuracy, we can predict 0.972 h in advance.

The RU can alleviate catastrophic forgetting and overfitting when classifying time series continuously. Figure S3 shows that all three medical datasets have multiple distributions in the

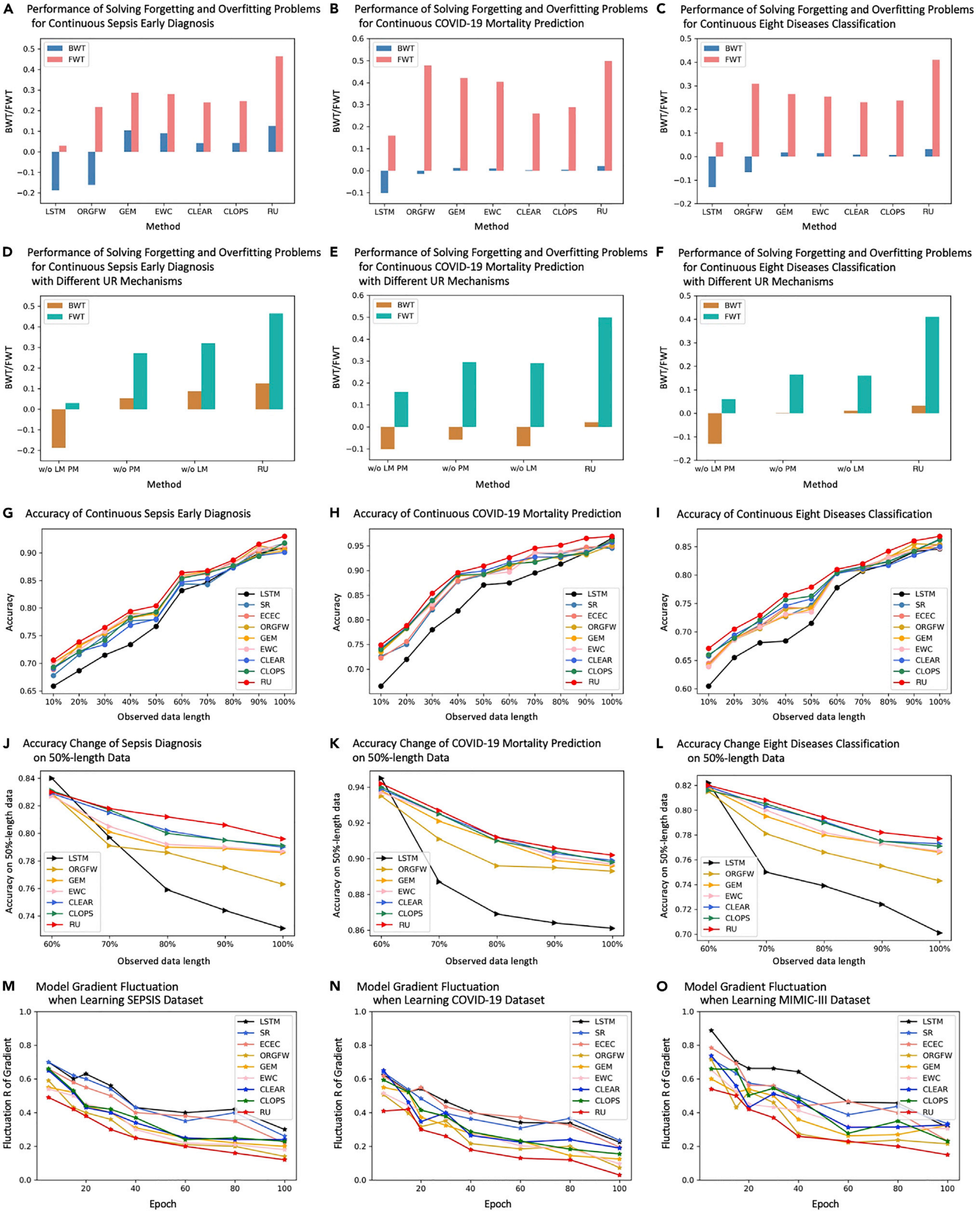

(legend on next page)

CCTS task. When learning multidistribution, as shown in Figures 3A–3C, the RU has the highest BWT and FWT, meaning it has the lowest negative influence that learning the new distribution has on old distributions and has the highest positive influence that learning the former data has on the overall task. Meanwhile, both LM and PM of the RU can contribute to the model performance. In Figures 3D–3F and S4 and Table S7, if we remove two mechanisms, respectively, the model performance will decline.

Finding 2: The change of importance coefficients interprets the learning process of the DL model

When a DL model learns time series in different stages, its parameters are updated constantly. After using the RU, if the model encounters a new data distribution, the importance coefficient is likely to change significantly. Thus, we can explain the learning process of the DL model from the perspective of the change of importance coefficient.

We divide the DL model into three blocks as shown in Figure 4. Block 1 is the input block. We focus on the parameter update process related to input features. For an input feature x^d (d-th dimension sequence of input multivariate time series), we use the overall importance coefficient of its related parameters to measure its importance: $\alpha^*(x^d) = \sum_n \alpha(\theta_{x^d, l_{1,n}})$, where $\theta_{x^d, l_{1,n}}$ is the weight between input feature x^d and the n-th neuron in layer l_1. Block 2 is the T-LSTM block. We focus on the parameter update process related to different gates. For a gate G_i, we use the overall importance coefficient of its parameters to measure its importance: $\alpha^*(G_i) = \sum_n \alpha(\theta_n)$. Block 3 is the output block. We focus on the parameter update process related to network neurons. For the j-th neuron in layer l_i, we use the overall importance coefficient of its output weights to measure its importance: $\alpha^*(l_{i,j}) = \sum_n \alpha(\theta_{l_{i,j}, l_{i+1,n}})$. The test on three blocks can enhance the interpretability based on the input data and network structures.

When the model learns time series with different lengths (in different time stages), its perceptual sensitivity to input features is different. As shown in Figures 4A1 and 4B1, for sepsis diagnosis, the blood pressure's α^* increases, which means that the model's perception of blood pressure improved in the later stage. For COVID-19 mortality prediction, lymphocytes' α^* is always high, which means that the model pays attention to this feature at all stages. Thus, α^* can be used to evaluate biomarkers.

We regard the importance coefficient of model parameters in the continuous learning process as a sequence and use the Bayesian online changepoint detection (BOCD) model[34] to find change points in this sequence. N change points divide the sequence into n+1 stages. As shown in Figures 4A2 and 4B2, in the output block, the training process of the model can be roughly divided into four stages for sepsis diagnosis and three stages for COVID-19 mortality prediction. In each stage, the important parameters are different. In the T-LSTM block, this change is obvious for the output gate but not obvious for the input and output gates. These observations reveal the intrinsic mechanism of model learning under the RU: for different stages of time series (different distributions), the DL model activates different neurons to perceive data. This also shows the potential of wide neural networks for CCTS. Networks with more neurons in one layer are more likely to learn multidistributed data.

Finding 3: Continuous prognosis reveals the disease biomarkers and stages

Semantically, the important feature is the input that has a great impact on the classification results. To quantify them, we define that the important feature is the input with a large overall importance coefficient α^*. Thus, we can find biomarkers of specific diseases: as shown in Figure 4A1 and 4B1, for sepsis, the biomarkers are heart rate (HR), respiration (Resp), mean arterial pressure (MAP), PaCO2, platelets count, total bilirubin, and creatinine. For COVID-19, the biomarkers are lymphocytes (lymph), lactic dehydrogenase (LDH), high-sensitivity C-reactive protein (hs-CRP), indirect bilirubin, creatinine, etc.

The response change of the model when learning disease records continuously can reflect the disease development. After finding change points by BOCD, the training process of the model can be divided into four stages for sepsis diagnosis and three stages for COVID-19 mortality prediction. Then, as shown in Figure 5, we visualize the hidden layer of block 3 and show the statistics of the corresponding characteristics.

Sepsis has four disease stages. Each stage has different reference levels of biomarkers. In some cases, the closer to the onset time, the greater the difference in biomarker reference levels among different prognoses. For example, in stage one (the interval from early 48 h to early 40 h before the onset time), the Resp difference between sepsis and non-sepsis is 1, while in stage four (the interval from early 6 h to the onset time), the Resp difference is 7. In other cases, reference levels of biomarkers with different prognoses are different at all stages, such as creatinine. These two conditions may explain the two mechanisms of sepsis: (1) the acute sepsis onset is reflected in the changes of some specific vital signs, e.g., a drop in blood pressure, increased lactate, and tachycardia. (2) Patients who have some congenital characteristics are more likely to get sepsis, e.g., nephropathy with abnormal creatinine and hepatopathy with increased total bilirubin.

COVID-19 has three disease stages. Compared with sepsis, two classes can be distinguished more clearly in the representation space. This also explains the higher accuracy of continuous COVID-19 mortality prediction than that of continuous sepsis diagnosis. Besides, in the presentation space, the hidden features of the two classes in the later stage are further apart. This shows the difficulty in early classification: the conflict between earliness and accuracy.

Figure 3. Method performances on continuous diagnosis and prognosis

The continuous classification performance is evaluated by backward transfer (BWT ↑) and forward transfer (FWT ↑). The classification accuracy is evaluated by the area under the curve of the receiver operating characteristic (AUC-ROC ↑). The 95% average AUC-ROC confidence intervals of the RU on SEPSIS and COVID-19 are (0.8624,0.8776) and (0.9477,0.9723). The RU is significantly better ($p < 0.05$) than LSTM ($p = 0.0116$); SR[17] ($p = 0.0293$); ECEC[18] ($p = 0.0321$); and ORGFW[19] ($p = 0.0201$) and is better than GEM[20] ($p = 0.0656$); EWC[21] ($p = 0.0676$); CLEAR[22] ($p = 0.0703$); and CLOPS[23] ($p = 0.0527$). And in the Bonferroni-Dunn test, $k = 9, n = 3, m = 5, q_{0.05} = 2.724, N = 15, \bar{r} = 3.53 > CD = 2.72$. Thus, the accuracy is significantly improved by the RU.

CellPress
OPEN ACCESS

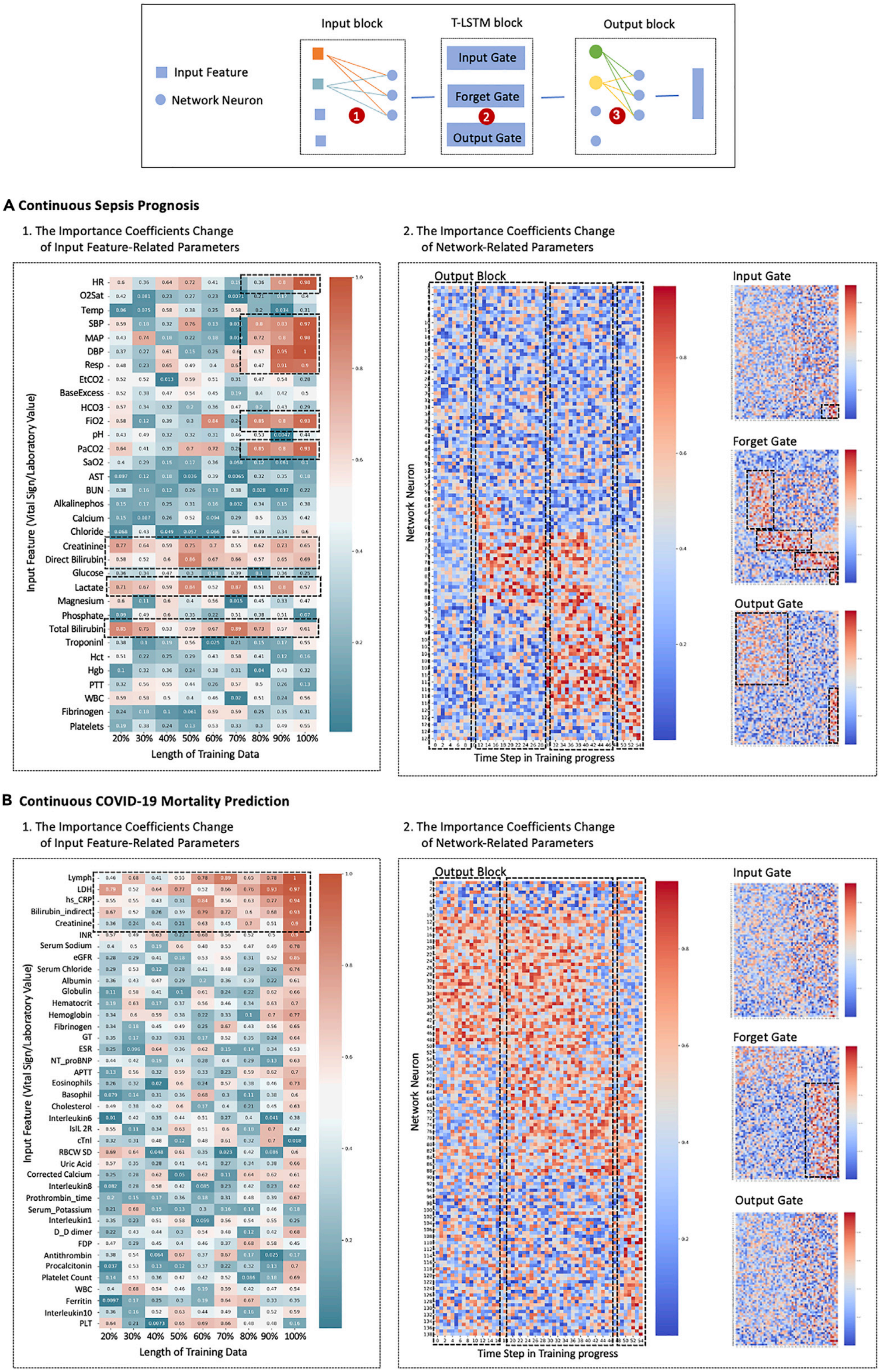

Figure 4. The importance of input features and network parameters

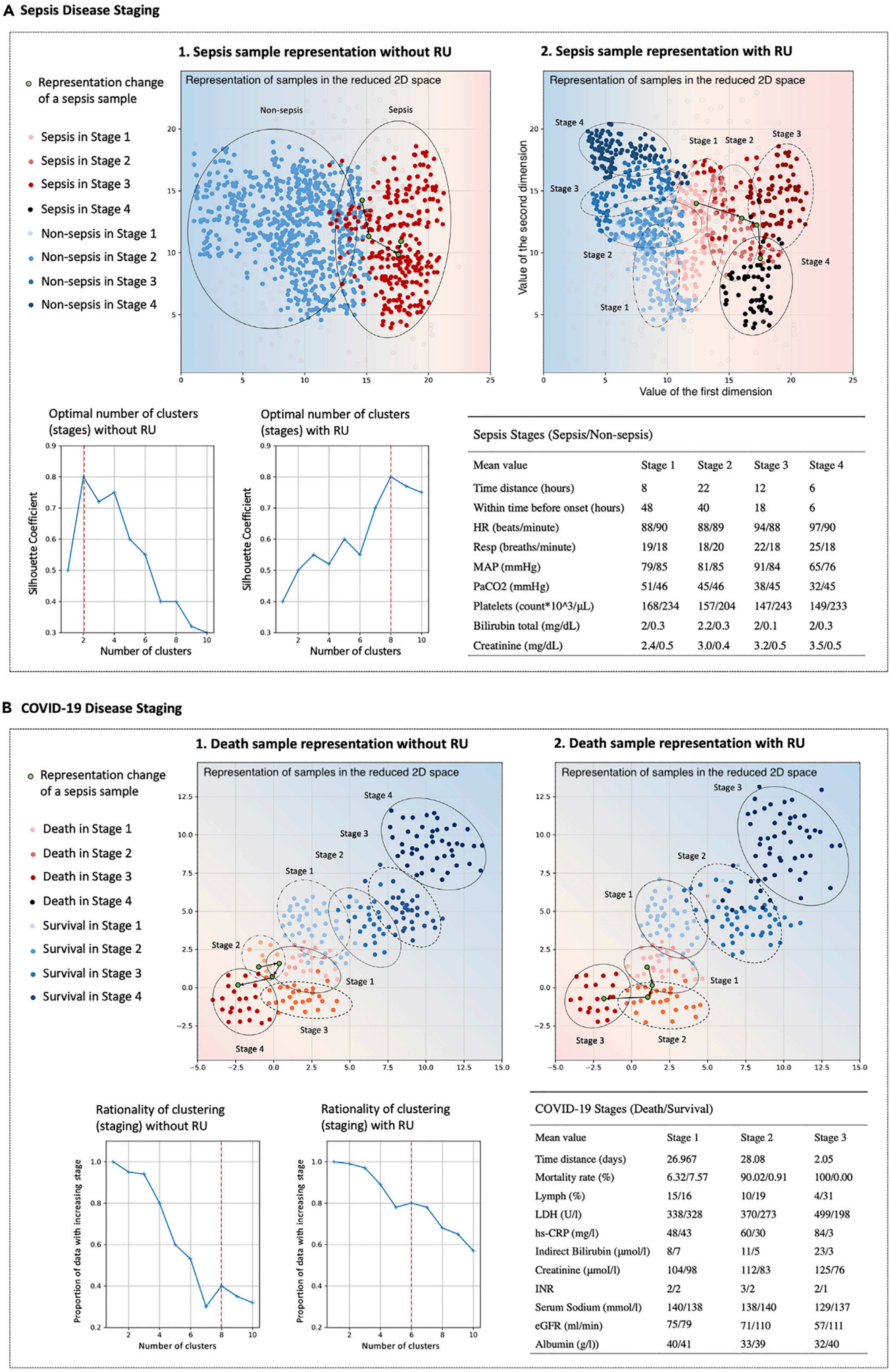

Sepsis Stages (Sepsis/Non-sepsis)

Mean value	Stage 1	Stage 2	Stage 3	Stage 4
Time distance (hours)	8	22	12	6
Within time before onset (hours)	48	40	18	6
HR (beats/minute)	88/90	88/89	94/88	97/90
Resp (breaths/minute)	19/18	18/20	22/18	25/18
MAP (mmHg)	79/85	81/85	91/84	65/76
PaCO2 (mmHg)	51/46	45/46	38/45	32/45
Platelets (count*10^3/μL)	168/234	157/204	147/243	149/233
Bilirubin total (mg/dL)	2/0.3	2.2/0.3	2/0.1	2/0.3
Creatinine (mg/dL)	2.4/0.5	3.0/0.4	3.2/0.5	3.5/0.5

COVID-19 Stages (Death/Survival)

Mean value	Stage 1	Stage 2	Stage 3
Time distance (days)	26.967	28.08	2.05
Mortality rate (%)	6.32/7.57	90.02/0.91	100/0.00
Lymph (%)	15/16	10/19	4/31
LDH (U/l)	338/328	370/273	499/198
hs-CRP (mg/l)	48/43	60/30	84/3
Indirect Bilirubin (μmol/l)	8/7	11/5	23/3
Creatinine (μmol/l)	104/98	112/83	125/76
INR	2/2	3/2	2/1
Serum Sodium (mmol/l)	140/138	138/140	129/137
eGFR (ml/min)	75/79	71/110	57/111
Albumin (g/l))	40/41	33/39	32/40

Figure 5. Disease stages and biomarkers of sepsis and COVID-19

Table 1. COVID-19 classification accuracy (AUC-ROC ↑) with non-uniform training sets and validation sets

	SR[a] [17]	ECEC[a] [18]	ORGFW[a] [19]	GEM[a] [20]	CLOPS[a] [23]	RU
Male	0.968 ± 0.014	0.969 ± 0.016	0.965 ± 0.004	0.978 ± 0.009	0.978 ± 0.014	0.971 ± 0.010
Female	0.935 ± 0.004	0.947 ± 0.015	0.938 ± 0.003	0.919 ± 0.008 ↓	0.921 ± 0.009[b]	0.947 ± 0.002[c]
Age ≤ 55	0.964 ± 0.012	0.965 ± 0.012	0.963 ± 0.007	0.971 ± 0.009	0.975 ± 0.010	0.972 ± 0.010
Age>55	0.906 ± 0.010[b]	0.908 ± 0.018[b]	0.917 ± 0.012	0.927 ± 0.010	0.907 ± 0.008[b]	0.941 ± 0.006[c]

Because indicators of blood sample and vital signs differ between males and females and change with age and the mortality rate of COVID-19 patients over 55 years old increases significantly,[35] we train models by samples of 224 male patients/179 patients whose age is ≤55 and validate models by samples of 151 female patients/196 patients whose age is >55. In the Bonferroni-Dunn test, $k = 6, n = 2, m = 5, q_{0.05} = 2.676, N = n \times m = 10, CD = 2.16, \bar{r} = 4.00 > CD$.

[a]The RU is significantly better, specifically, than baselines with p = 0.0449, 0.0498, 0.0384, 0.0243, and 0.0204 ($p < 0.05$).

[b]The accuracy is greatly reduced over 5%.

[c]The smallest decline in accuracy.

The classification of eight diseases has four stages as shown in Figure S8. Since the task is not specific to a disease, we call task stages and important features instead of disease stages and biomarkers. The important features found in the four stages illustrate the necessity of continuous vital sign monitoring, blood routine examination during hospitalization, and detailed laboratory examination in later stages. Meanwhile, there is a big difference between the last three stages and the first stage. It implies that patients' states have changed significantly since the second stage.

Finding 4: RU enhances the model for atypical scenarios and sustainable use

The RU can avoid model overfitting and guarantee certain model generalization. As shown in Tables 1, S10, and S11, we divide datasets according to gender and age; for most baselines, the accuracy on the validation set is much lower than that on the training set, but the RU helps the DL model to maintain robustness.

Meanwhile, the RU can prevent the result difference caused by the different orders of training sets. The method we have introduced is to use time series of different stages to train the model, and the order is based on time. Another order is the data similarity[23] as shown in Figure S6. For example, as many vital signs are periodic, the cycle of blood pressure is 1 day. Therefore, after using vital signs within 24 h, we will use the data within 25 h according to the time order but use the data within 48 h according to the similarity order. No matter what order is adopted, the RU has stable accuracy as shown in Tables 2, S8, and S9. It shows the potential of PM's global optimization and the potential of the RU's sustainable use.

Furthermore, the RU is a data-agnostic, model-agnostic, and easy-to-use plug in. It can not only improve the accuracy of continuous classification of medical time series but also plays a role in other fields. For example, as shown in Table 3, the RU outperforms baselines on meteorological data for tasks of continuous earthquake early warning and rainfall prediction. Figures S2–S6 show the ability of the RU in more scenarios. The RU can also be used to train other DL models such as the convolutional neural network (CNN) and transformer.[36] It is easy to use and does not need to change the network structure. As shown in Table 4, if we use the RU to train base models, the accuracy can be improved by more than 5%, and the RU is not limited by hyper-parameters. The hyper-parameters are ρ and λ. ρ determines the correlation between current and previous gradients in Equation 12. We find that PM performs well when ρ is the same as the learning rate $\rho^m = \eta^m = \frac{1}{(t+1)^a}, a = 1$. λ decides the constraint degree on parameter update in Equation 5. We can optimize it using the search method supplied by mature tools.

DISCUSSION

DL has the potential to explore disease mechanisms

The importance coefficient not only explained the working mechanism of the DL model but also dug out the disease biomarkers and stages.[39] Different from the statistics and case analysis of the medical gold standard, these biomarkers are based on the judgment basis of the DL model. It can provide a new horizon for medical research. For example, based on the learning process of the DL model, for sepsis, a drop in

Table 2. Classification accuracy (AUC-ROC ↑) of RU after learning training sets by different orders

		20%[a]	40%	60%	80%	100%
SEPSIS	time order	0.735 ± 0.003	0.826 ± 0.003	0.841 ± 0.003	0.860 ± 0.005	0.872 ± 0.001
	similarity order	0.734 ± 0.006	0.824 ± 0.004	0.843 ± 0.004	0.863 ± 0.007	0.870 ± 0.002
COVID-19	time order	0.789 ± 0.002	0.902 ± 0.002	0.926 ± 0.000	0.959 ± 0.001	0.968 ± 0.000
	similarity order	0.790 ± 0.001	0.910 ± 0.003	0.924 ± 0.001	0.958 ± 0.001	0.967 ± 0.000
MIMIC-III	time order	0.702 ± 0.006	0.756 ± 0.005	0.820 ± 0.007	0.852 ± 0.005	0.876 ± 0.004
	similarity order	0.704 ± 0.007	0.755 ± 0.004	0.818 ± 0.004	0.853 ± 0.004	0.876 ± 0.003

In the Bonferroni-Dunn test, $k = 2, n = 3, m = 5, q_{0.05} = 1.960, N = n \times m = 15, CD = 0.51, r(\text{time}) = r(\text{similarity}) = 1.33 < CD$. Thus, two training orders have similar effects.

[a]k% means the current classification time is k% of the total time of the full-length time series.

Table 3. Performance (AUC-ROC ↑, BWT ↑) for two meteorological datasets

		SR[a]	ECEC[a]	ORGFW[a]	GEM[a]	CLOPS[a]	RU
UCR-EQ	AUC-ROC	0.902 ± 0.002	0.909 ± 0.010	0.920 ± 0.001	0.921 ± 0.001	0.919 ± 0.004	0.931 ± 0.004[b]
	BWT	0.003	0.033	0.112	0.123	0.149	0.162[b]
USHCN	AUC-ROC	0.911 ± 0.012	0.902 ± 0.012	0.916 ± 0.004	0.920 ± 0.003	0.921 ± 0.005	0.930 ± 0.005[b]
	BWT	0.034	0.047	0.072	0.098	0.082	0.124[b]

[a]The UCR-EQ dataset[28] has 471 earthquake records from the UCR time series classification archive. It is the univariate time series of seismic feature value. Natural disaster early warning, like earthquake warning, helps to reduce casualties and property losses.[37] The USHCN dataset[27] has the daily meteorological data of 48 states in the US from 1887 to 2014. It is the multivariate time series of five weather features. Rainfall warning is not only a demand of daily life but can also help prevent natural disasters.[38] In the Bonferroni-Dunn test, $k = 6, n = 2, m = 5, q_{0.05} = 2.676, N = n \times m = 10, CD = 2.16, \bar{r} = 4.00 > CD$. RU is significantly better, specifically, better than baselines with p = 0.0.0004, 0.0241, 0.0099, 0.0137, and 0.0329 ($p < 0.05$).
[b]The best performance.

blood pressure, increased lactate, and tachycardia are important in the later stage, while abnormal creatinine and total bilirubin are always important. It can be explained that sepsis is an acute disease and is related to some congenital diseases like nephropathy and hepatopathy. Such behavior is in exact accordance with the sepsis literature.[40] For COVID-19, only lymph, LDH, and hs-CRP are most important throughout the stages. This shows that COVID-19 has a clear reference to measure the disease severity.[41] Meanwhile, we match the disease stage with the change in model parameters during the learning process. In this way, the disease stage is no longer defined only by the biomarker level or patient subtyping but by the characteristic changes in the high-dimensional space created by DL.

RU helps with disease staging

At present, except for cancers, it is difficult to define clear stages for most diseases. For sepsis, disease stratification is implemented by recommended clinical criteria (e.g., systemic inflammatory response syndrome [SIRS], sequential organ failure assessment [SOFA], quick SOFA [qSOFA], etc.), but they focus on severity and not the progression. We emphasize that disease staging is the disease change over time. The RU can achieve this according to the model change when learning the medical time series from different time stages. As shown in Figure 5A, the RU can identify stages directly according to the importance coefficient change of model parameters, instead of using unsupervised clustering methods. Without the RU, the clustering method has trouble finding the stages. The number of clusters with the best silhouette coefficient is 2, and the number of stages is 1. For COVID-19, most work categorizes it roughly into early stage and late stage.[42] Some existing DL-based methods can perform disease staging by using representation learning. For example, our previous work[32] clustered features in hidden layers of T-LSTM and got four COVID-19 stages. As shown in Figure 5B1, this clustering-based method can get a good silhouette coefficient but cannot guarantee the time constraint. When identifying these four stages, only about 40% of the samples will be divided into stages corresponding to chronological order. For example, a death sample (green dot) is initially judged to be stage two, then stage one, and finally stage four. But stages 1–4 are in time order. Using the RU, this inconsistency is largely alleviated: the percentage of samples with a time-increasing stage is raised. The death sample (green dot) is judged as stage one, then stage two, and finally stage three over time.

Learning multidistributed data is the general trend

Currently, many sophisticated DL models have shown outstanding achievements in time series modeling in many fields. For offline learning, after the model has learned the dataset, the model is only sensitive to the learned distribution. For example, when the model has learned the full-length vital signs of sepsis, it usually classifies accurately at the onset time, but it is too late for critical illness. To gain treatment time, the model needs to learn early data. However, there are also problems in learning early data at only one stage. For example, a time series may have missed the learned stage at the beginning, the characteristics of early data are not obvious and require late data assistance, etc. Thus, it is necessary for the model to learn time series from different

Table 4. Performance (AUC-ROC ↑/BWT ↑) improvement of different neural networks after using RU

	SEPSIS	COVID-19	MIMIC-III	UCR-EQ	USHCN
LSTM[a]	0.837 ± 0.008/0.002	0.909 ± 0.003/0.047	0.786 ± 0.002/0.054	0.881 ± 0.004/0.032	0.891 ± 0.003/0.054
+RU	0.907 ± 0.008/0.065[b]	0.969 ± 0.003/0.115[b]	0.856 ± 0.002/0.102	0.931 ± 0.004/0.162[b]	0.930 ± 0.005/0.124
CNN[a]	0.848 ± 0.002/0.004	0.903 ± 0.002/0.037	0.784 ± 0.004/0.032	0.878 ± 0.005/0.030	0.881 ± 0.004/0.057
+RU	0.904 ± 0.003/0.067[b]	0.960 ± 0.006/0.095[b]	0.832 ± 0.002/0.099	0.929 ± 0.006/0.150[b]	0.922 ± 0.005/0.118
Transformer[a]	0.843 ± 0.011/0.005	0.906 ± 0.005/0.040	0.784 ± 0.006/0.059	0.889 ± 0.010/0.029	0.880 ± 0.015/0.059
+RU	0.903 ± 0.008/0.067[b]	0.960 ± 0.007/0.109[b]	0.852 ± 0.008/0.124[b]	0.920 ± 0.008/0.132	0.921 ± 0.008/0.120

[a]The accuracy is significantly improved after using the RU ($p < 0.05$), specifically p = 0.0040, 0.0000, and 0.0015.
[b]The accuracy is increased by more than 5%, and the BWT is increased by more than 5%.

CellPress
OPEN ACCESS

stages, i.e., multidistributed data.[43] In this way, the model can realize CCTS.

Reasonable training strategy is the icing on the cake

We help the DL model to learn multidistributed data from the perspective of model updating strategy. Our empirical study shows that a meaningful model training strategy plays a key role in improving the model performance and generalization power. Compared with the study of model structure design, the application of strategy-based design is more extensive. It pays more attention to the overall goal and has few requirements for specific data and used models, meaning it is data agnostic, model agnostic, and easy to use.

Quantifying the updating process makes it possible to interpret the DL model

Interpretability remains one of the key issues to be solved to achieve the trust of clinicians and insert the DL algorithm into clinical workflow.[44] DL models are often considered to be black box because they typically have high-dimensional non-linear operations, many model parameters, and complex model architectures, which makes them difficult for a human to understand. In this work, the RU implements CCTS by updating model parameters with constraints. The constraint is achieved by quantifying the importance of the parameters. Surprisingly, the importance of parameters can be used to explain the DL model. As shown in experimental results, it can identify both the input features and the structural parts that are important for classification. Figure S7 shows the important samples in continuous binary classification. In fact, measuring parameter changes is not only suitable for the dynamic process in CCTS but also provides the possibility to interpret the learning processes of DL models.

Opportunities of CCTS

Currently, some subdisciplines (online learning, continual learning, anomaly detection) also study the mode of continuous classification, but their setting methods cannot satisfy the summarized requirements simultaneously (see related work in the supplemental information). CCTS is a new concept and a potential task that we propose when facing practical problems.

Meanwhile, we find that the neural network structure with a reasonable width is more conducive to continuous classification and continual learning because with the learning of the new data distribution, the change of important parameters on the scale of network width is more obvious and regular but that on the scale of depth is confused. Therefore, future work can study the impact of model structure on CCTS from the perspective of network depth and network width.

Besides, the imbalanced data are very common in many real-world datasets. For example, in electronic health records, the records of common diseases are much more than those of rare diseases. And for classification task, the lack of data in a minority class may lead to uneven accuracy. When encountering such data, although not involved in this study, we can change the cross-entropy loss $\mathcal{L}$ in Equation 5 to other specific losses such as weighted cross-entropy, mean false error loss, and focal loss.[45]

Further, we find that different learning orders have little effect on our method. This demonstrates the potential of our approach for offline continual learning: We use the existing data to train the model and put it into use. After a period of time, some new data may be generated. We can continue to train the current model with the new data instead of designing a new model. In addition, the method for CCTS can be context independent in the future. The model can perform not only different medical tasks but also tasks in other fields like meteorology.

EXPERIMENTAL PROCEDURES

Resource availability

Lead contact

Request for information and resources used in this article should be addressed to Prof. Shenda Hong (hongshenda@pku.edu.cn).

Materials availability

No new materials were generated by this study.

Data and code availability

All datasets are publicly available. SEPSIS: https://doi.org/10.13026/v64v-d857,[24] COVID-19: https://doi.org/10.1038/s42256-020-0180-7,[25] MIMIC-III: https://doi.org/10.13026/C2XW26,[26] USHCN: NCDC (DSI-3200, DSI-3206 and DSI-3210),[27] UCR https://www.cs.ucr.edu/~eamonn/time_series_data_2018/,[28] and ACTIV: https://archive.ics.uci.edu/ml/datasets/Localization+Data+for+Person+Activity.[29] All original code has been deposited at Zenodo under https://doi.org/10.5281/zenodo.7496021 (https://github.com/SCXsunchenxi/CCTS) and is publicly available as of the date of publication. Any additional information required to reanalyze the data reported in this paper is available from the lead contact upon request.

Methods

CCTS

Definition 1 (CCTS). A dataset $\mathcal{T} = \{X^n\}_{n=1}^N$ contains N time series. Each time series $X = \{x_m\}_{m=1}^M$ has M observations with value x_m at time t_m. At the final time t_M, X is labeled with a class $C \in \mathcal{C}$. As time series vary among time, dataset $\mathcal{T}^* = \{X_{1:m}^n\}_{n=1}^N$, consisting of subsequence $X_{1:m}$ of each X, has a distribution $\mathcal{D}_m$. Thus, $\mathcal{T}_m^* = \{X_{1:m}^n\}_{n,m=1}^{N,M}$ has a series distribution $\mathcal{D} = \{\mathcal{D}_m\}_{m=1}^M$. CCTS learns every $\mathcal{D}_m$ and introduces a task series $\mathcal{M} = \{\mathcal{M}^m\}_{m=1}^M$ to minimize the additive risk $\sum_{m=1}^M \mathbb{E}_{\mathcal{M}^m}[\mathcal{L}(f^m(\mathcal{D}_m;\theta), C)]$ with model f and parameter θ. f^m is the model f after being trained for $\mathcal{M}^m$, and its performance on all observed data cannot degrade: $\frac{1}{m}\sum_{i=1}^m \mathcal{L}(f^i, \mathcal{M}^i) \leq \frac{1}{m-1}\sum_{i=1}^{m-1} \mathcal{L}(f^i, \mathcal{M}^i)$.

Notations are summarized in Table S1. Without the loss of generality, we use the univariate time series to present the problem. Multivariate time series can be described by changing x_m to x_m^d. d is the d-th dimension. The classification task uses cross-entropy loss:

$$\mathcal{L}(f^m, \mathcal{M}^m) = \mathcal{L}(f^m, \{\mathcal{T}_m^*, \mathcal{C}\}) = -\frac{1}{N}\sum_{n=1}^{N}\sum_{c=1}^{|\mathcal{C}|} C_c \log \widehat{C}_c \qquad \text{(Equation 1)}$$

T-LSTM

The real-world time series, especially vital signs, have long sequences and are irregularly sampled. The classical RNN only processes uniformly distributed longitudinal data by assuming that the sequences have an equal distribution of time differences. Thus, we implement T-LSTM.[32]

$$\begin{aligned}
C_{m-1}^S &= \tanh(W_d C_{m-1} + b_d) \quad \text{short} - \text{term memory} \\
\widehat{C}_{m-1}^S &= C_{m-1}^S \cdot g(\Delta^m) \quad \text{discounted short} - \text{term memory} \\
C_{m-1}^T &= C_{m-1} - C_{m-1}^S \quad \text{long} - \text{term memory} \\
C_{m-1}^* &= C_{m-1}^T - \widehat{C}_{m-1}^S \quad \text{adjusted previous memory} \\
f_m &= \sigma(W_f x_m + U_f h_{m-1} + b_f) \quad \text{forget gate} \\
i_m &= \sigma(W_i x_m + U_i h_{m-1} + b_i) \quad \text{input gate} \\
\tilde{C}_m &= \tanh(W_c x_m + U_c h_{m-1} + b_o) \quad \text{candidate memory} \\
C_m &= f_m \cdot C_{m-1}^* + i_m \cdot \tilde{C}_m \quad \text{current memory} \\
o_m &= \sigma(W_o x_m + U_o h_{m-1} + b_o) \quad \text{output gate} \\
h_m &= o_m \cdot \tanh(C_m) \quad \text{current hidden state}
\end{aligned}$$

Algorithm 1. RU

Input:The DL model f (T-LSTM) and a defined task set $\mathcal{M}$.
Output:The trained model f
Initialize a model f^0 with parameter θ^0
Initialize a gradient memory $\mathcal{G} \leftarrow \{\}$
Initialize parameters $\eta^m, \rho^m = \left(\frac{1}{m+1}\right)^a$
for $m = 1$ to $|\mathcal{M}|$ do
  Extract current task $\mathcal{M}^m \leftarrow \mathcal{M}$
  Get loss $\mathcal{O}^m \leftarrow$ Equation 5 ▷ Limitation Mechanism
  $g^{m-1} \leftarrow \mathcal{G}$
  $d^m \leftarrow$ Equation 12
  $v^m \leftarrow$ Equation 8
  $g^m, \theta^m \leftarrow$ Equation 9 ▷ Promotion Mechanism
  $\mathcal{G} \leftarrow g^m$
  Get model $f^m \leftarrow \theta^m$
end for
Output model $f \leftarrow f^{|\mathcal{M}|}$

T-LSTM has some new designs. The C^S_{m-1} component learns the short-term memory of a sequence by learnable network parameters. C^T_{m-1} is the long-term memory calculated from the former memory cell C_{m-1} by getting rid of C^S_{m-1}. C^S_{m-1} is adjusted to the discounted short-term memory $\widehat{C}^S_{m-1}$ by the elapsed time function $g(\Delta_m)$. The previous memory C^*_{m-1} is changed to the complement subspace of C^T_{m-1} combined with $\widehat{C}^S_{m-1}$. We use a log calculation for the elapsed time function. Δ_m describes the time gap between two records at two adjacent time points t_m and t_{m-1}.

$$g(\Delta_m) = \frac{1}{\log(e + \Delta_m)}, \Delta_m = t_m - t_{m-1} \quad \text{(Equation 2)}$$

RU

LM. When the model meets a distribution, it will change from f^{m-1} to f^m. In order to let the model performance on all tasks not degrade, the loss $\mathcal{L}$ of the current f^m on tasks $\{\mathcal{M}^k\}^m_{k=1}$ should be not bigger than that of the previous f^{m-1} on tasks $\{\mathcal{M}^k\}^{m-1}_{k=1}$:

$$\min_{\theta^m} \mathcal{L}(f^m(X_{1:m}, \theta^m), C)$$
$$\text{subject to } \frac{1}{m}\sum_{k=1}^{m}\mathcal{L}(f^m, \mathcal{M}^k) \le \frac{1}{m-1}\sum_{k=1}^{m-1}\mathcal{L}(f^{m-1}, \mathcal{M}^k) \quad \text{(Equation 3)}$$

Based on the observation of many parameter configurations resulting in the same performance, we could add a regular term to the loss to restrict the updating of model parameters. Thus, in LM, we constrain important parameters to stay close to their old values but change $\theta_{\text{unimportant}}$ more. We give a new loss in Equation 4. A regularization term is added to the original loss $\mathcal{L}$, where α is the importance coefficient of parameter θ. With the minimum $\mathcal{O}$, θ^m will be changed less from θ^{m-1} with a large α. In Figure 2, $\theta_{\text{important}}$ is limited in a region.

$$\mathcal{O}(\theta^m) = \mathcal{L}(f^m(\theta^m), \mathcal{M}^m) + \lambda\sum_i \alpha_i\left(\theta^m_i - \theta^{m-1}_i\right)^2 \quad \text{(Equation 4)}$$

The second derivative of probability can evaluate the importance coefficient $\alpha = (\log p\,(D_m|0^m))''$. Elastic weight consolidation[21] defines α from a probabilistic perspective $\log p(\theta|\mathcal{D}) = \log p(D_m|\theta) + \log p(\theta|D_{m-1}) - \log p(D_m)$. Optimizing the parameters is tantamount to finding their most probable values under $\mathcal{D}$. The posterior probability is indicated by Laplace approximation. We use the diagonal of a Fisher information matrix to represent the first-order derivatives $F = \frac{1}{N}\sum_{k=1}^{N}\nabla\log p(D_k|\theta)\nabla\log p(D_k|\theta)^{\top}$, represent α by F, and rearrange Equation 4 to

$$\mathcal{O}(\theta^m) = \mathcal{L}(f^m(\theta^m), \mathcal{M}^m) + \lambda\sum_i \mathrm{F}_i\left(\theta^m_i - \theta^{m-1}_i\right)^2$$
$$\mathrm{F}_i = \frac{1}{m}\sum_{k=1}^{m}\left(\frac{\partial\log p(D^k|\theta^m_i)}{\partial\theta^m_i}\right)^2 \quad \text{(Equation 5)}$$

PM. When we focus on the final task, if the model meets a new distribution, the learned knowledge will be part of the final solution. We regard this as a continuous optimization problem and treat different data distributions equally. The new data helps the model learn the old data, which can reduce the unstable solution caused by the different learning orders. The continuous optimization problem is defined as regret minimization. For task $\mathcal{M}$, the regret $\mathcal{R}$ is the difference between the total loss and that of the best parameter θ^* of the fixed decision in hindsight.

$$\mathcal{R}_M := \sum_{m=1}^{M}\left(\mathcal{L}(f^m(\theta^m), \mathcal{M}) - \mathcal{L}(f^m(\theta^{m*}), \mathcal{M})\right) \quad \text{(Equation 6)}$$

For regret minimization, we design PM by projection-free mechanisms and stochastic recursive gradient. It focuses the quality of the final performance instead of iterates produced from the course of optimization. The main bottleneck is the computation of projections onto the underlying decision set $\prod_{\mathcal{K}}(\theta) = \operatorname{argmin}_{\widehat{\theta}\in\mathcal{K}}\|\widehat{\theta} - \theta\|$. The projection operation is defined as the closest point inside the convex set $\mathcal{K}$ of Euclidean space to a given point. The projection-free method can replace the projection with a linear optimization at each iteration. It alleviates the complexity but remains problems of training non-converging and instability.

Thus, we estimate a stochastic recursive estimator based on stochastic gradient technology.[19] Assuming for task $\mathcal{M}^m$, the model receives a new distribution $\mathcal{D}_m$ and gets the loss $\mathcal{L}$. We first give a random variable ξ^m to satisfy $\mathbb{E}_{\xi^m\sim D_m}[\nabla L(\theta^m, \xi^m)] = \nabla\sum_{m=1}^{M}\mathcal{L}(f^m(X_{1:m}, \theta^m), C)$. Then, the stochastic recursive estimator is

$$d^m = \nabla\mathcal{L}(\theta^m, \xi^m) + (1 - \rho^m)\left(d^{m-1} - \nabla\mathcal{L}\left(\theta^{m-1}, \xi^m\right)\right) \quad \text{(Equation 7)}$$

It finds a solution v^m of the linear optimization problem

$$v^m = \operatorname{argmin}_{v\in\mathcal{K}}\|d^m, v^m\|_2 \quad \text{(Equation 8)}$$

to update θ^m in the direction of gradient g^m:

$$g^m = v^{m-1} - \theta^{m-1}, \theta^m = \theta^{m-1} + \eta^{m-1} \cdot g^m \quad \text{(Equation 9)}$$

Such a method randomly selects samples to guide the change of gradient and leads to faster converges.

Overall training process. When the new gradient g_m and the old gradient g_k are at an acute angle, the model performance dose not decrease and even improves[20]:

$$\langle g^m, g^k \rangle = \langle \frac{\partial \mathcal{L}(f^m, \mathcal{M}^m)}{\partial \theta^m}, \frac{\partial \mathcal{L}(f^k, \mathcal{M}^k)}{\partial \theta^k} \rangle \geq 0 \quad k = 1, \ldots, m-1 \quad \text{(Equation 10)}$$

The regularization projects g_m to the closest gradient g'_m by satisfying all the constraint of acute angle:

$$\min \| g^m - g^{m'} \|_2 \quad \text{subject to } \langle g^k, g^{m'} \rangle \geq 0, k = 1, \ldots, m-1 \quad \text{(Equation 11)}$$

In LM, F is positive semi-definite. This property not only guarantees that seeing each task as a factor of the posterior (LM) but also guarantees the acute angle change of a vector after the product (PM). Thus, the RU updates network parameters by using the regularized loss $\mathcal{O}$ in Equation 5, and we re-arrange Equations 7, 8, 9, 10, 11, and 12. $\eta^m, \rho^m = \left(\frac{1}{m+1}\right)^a$.

$$d^m \leftarrow \nabla_{\theta^m} \mathcal{O}^m + (1 - \rho^m)(d^{m-1} - g^{m-1}) \quad \text{(Equation 12)}$$

Algorithm 1 gives the algorithm description of RU. In practice, we optimize hyper-parameters using the search method supplied by mature tools. Figure S5 shows that the method performs better when $a = 0.933\lambda + 0.907$.

Regret and complexity. PM holds a nearly optimal regret bound $\tilde{O}(\sqrt{M})$ with probability at least $1 - \delta$ for any $\delta \in (0,1)$, $\mathcal{R}_M \leq (logM + 1)(f(\theta^1) - f(\theta^*)) + (16LD^2 + 16\sigma + 4B)\sqrt{2Mlog\frac{8M}{\delta} + \frac{1}{2}LD^2(logM+1)^2}$. D is diameter of convex set, and L is L-Lipschitz continuous. PM and LM achieve a $O(1)$ per-round computational cost. If the complexity of training a base model to convergence is O and data length is M, the overall complexity will be MO (see supplemental mathematics).

Evaluation metrics

The classification accuracy is evaluated by assessing the AUC-ROC (the higher the better). The ROC is a curve of the true positive rate (TPR) and the false positive rate (FPR). TN, TP, FP, and FN represent true positive, true negative, false positive, and false negative, respectively.

$$TPR = \frac{TP}{TP + FN}, FPR = \frac{FP}{TN + FP} \quad \text{(Equation 13)}$$

The AUC confidence interval is equal to $AUC \pm se \cdot z_{crit}$. z_{crit} is the two-tailed critical value of the standard normal distribution NORM.S.INV$(1 - \alpha/2)$, $\alpha = 0.05$. se is Equation 14, where s_1 and s_2 are the sizes of the two samples in different labels and $q_0 = AUC(1 - AUC)$, $q_1 = AUC/(2 - AUC) - AUC^2$, $q_2 = 2AUC^2/(1 + AUC) - AUC^2$. For example, in the SEPSIS dataset, $s1 = 27,977$ (non-sepsis) and $s2 = 2,359$ (sepsis), and in COVID-19 dataset, $s1 = 201$ (survival) and $s2 = 174$ (death).

$$se = \sqrt{\frac{q_0 + (n_1 - 1)q_1 + (n_2 - 1)q_2}{n_1 n_2}} \quad \text{(Equation 14)}$$

The statistical significance is evaluated by the Bonferroni-Dunn test, and k, n, m, and q are the number of methods, the number of datasets, the number of cross-validation folds, and the critical value, respectively. If the average rank of baselines $\bar{r} > CD$, the tested method is significantly better.

$$CD = q\sqrt{\frac{k(k+1)}{6N}}, N = n \times m \quad \text{(Equation 15)}$$

Learning stability is evaluated by the gradient fluctuation R (the lower the better). It quantifies the frequency of gradient direction changes during training.

$$R = \frac{1}{n-1}\sqrt{\sum_{i=1}^{n}(d_i - d_{i-1})^2}, d = \begin{cases} -1, \text{if } g < 0 \\ 1, \text{if } g > 0 \end{cases} \quad \text{(Equation 16)}$$

The continuous classification performance is evaluated by the BWT and FWT (the higher the better). They are the influences that learning a new distribution have on old and future distributions. $R_{i,j}$ is the accuracy on distribution D_j after completing task D_i. $\bar{b}$ is the accuracy with random initialization.

$$\begin{aligned} BWT &= \frac{1}{|\mathcal{D}| - 1}\sum_{i=1}^{|\mathcal{D}|-1} R_{|\mathcal{D}|,i} - R_{i,i} \\ FWT &= \frac{1}{|\mathcal{D}| - 1}\sum_{i=2}^{|\mathcal{D}|} R_{i-1,i} - \bar{b}_{i,i} \end{aligned} \quad \text{(Equation 17)}$$

SUPPLEMENTAL INFORMATION

Supplemental information can be found online at https://doi.org/10.1016/j.patter.2023.100687.

ACKNOWLEDGMENTS

This work was supported by the National Natural Science Foundation of China (nos. 62102008 and 62172018) and the National Key Research and Development Program of China under grant 2021YFE0205300.

AUTHOR CONTRIBUTIONS

Conceptualization, C.S., S.H., and H.L.; methodology, C.S.; validation, C.S., M.S., D.C., and B.Z.; investigation, C.S.; writing – original draft, C.S.; writing – review & editing, C.S., H.L., M.S., D.C., B.Z., and S.H.; funding acquisition, H.L. and S.H.; supervision, H.L. and S.H.

DECLARATION OF INTERESTS

The authors declare no competing interests.

INCLUSION AND DIVERSITY

We support inclusive, diverse, and equitable conduct of research.

Received: September 26, 2022
Revised: November 7, 2022
Accepted: January 11, 2023
Published: February 3, 2023

REFERENCES

1. Singer, M., Deutschman, C.S., Seymour, C.W., Shankar-Hari, M., Annane, D., Bauer, M., Bellomo, R., Bernard, G.R., Chiche, J.D., Coopersmith, C.M., et al. (2016). The third international consensus definitions for sepsis and septic shock (sepsis-3). JAMA, J. Am. Med. Assoc. *315*, 801–810. https://doi.org/10.1001/jama.2016.0287.

2. Seymour, C.W., Gesten, F., Prescott, H.C., Friedrich, M.E., Iwashyna, T.J., Phillips, G.S., Lemeshow, S., Osborn, T., Terry, K.M., and Levy, M.M. (2017). Time to treatment and mortality during mandated emergency care for sepsis. N. Engl. J. Med. *376*, 2235–2244. https://doi.org/10.1016/j.jemermed.2017.08.088.

3. Zong, N., Li, N., Wen, A., Ngo, V., Yu, Y., Huang, M., Chowdhury, S., Jiang, C., Fu, S., Weinshilboum, R., et al. (2022). BETA: a comprehensive benchmark for computational drug-target prediction. Brief. Bioinform. *23*, bbac199. https://doi.org/10.1093/bib/bbac199.

4. Wanyan, T., Honarvar, H., Jaladanki, S.K., Zang, C., Naik, N., Somani, S., De Freitas, J.K., Paranjpe, I., Vaid, A., Zhang, J., et al. (2021). Contrastive learning improves critical event prediction in COVID-19 patients. Patterns *2*, 100389. https://doi.org/10.1016/j.patter.2021.100389.

5. Rambhatla, S., Zeighami, S., Shahabi, K., Shahabi, C., and Liu, Y. (2022). Toward accurate spatiotemporal COVID-19 risk scores using high-resolution

real-world mobility data. ACM Trans. Spat. Algorithms Syst. *8*, 1–30. https://doi.org/10.1145/3481044.

6. Goodman, B., and Flaxman, S. (2017). European Union regulations on algorithmic decision-making and a "right to explanation". AI Mag. *38*, 50–57. https://doi.org/10.1609/aimag.v38i3.2741.
7. Tsang, M., Rambhatla, S., and Liu, Y. (2020). How does this interaction affect me? interpretable attribution for feature interactions. In Proceedings of the Advances in Neural Information Processing Systems (NeurIPS), pp. 6147–6159.
8. Cao, S., Wang, J.R., Ji, S., Yang, P., Dai, Y., Guo, S., Montierth, M.D., Shen, J.P., Zhao, X., Chen, J., et al. (2022). Estimation of tumor cell total mrna expression in 15 cancer types predicts disease progression. Nat. Biotechnol. *40*, 1624–1633. https://doi.org/10.1038/s41587-022-01342-x.
9. Si, N., Zhang, F., Zhou, Z., and Blanchet, J. (2020). Distributionally robust policy evaluation and learning in offline contextual bandits. In Proceedings of the International Conference on Machine Learning (ICML), pp. 8884–8894.
10. Chen, L., Harshaw, C., Hassani, H., and Karbasi, A. (2018). Projection-free online optimization with stochastic gradient: from convexity to submodularity. In Proceedings of International Conference on Machine Learning (ICML), pp. 814–823.
11. Fu, T., Huang, K., Xiao, C., Glass, L.M., and Sun, J. (2022). HINT: hierarchical interaction network for clinical-trial-outcome predictions. Patterns *3*, 100445. https://doi.org/10.1016/j.patter.2022.100445.
12. Hannun, A.Y., Rajpurkar, P., Haghpanahi, M., Tison, G.H., Bourn, C., Turakhia, M.P., and Ng, A.Y. (2019). Cardiologist-level arrhythmia detection and classification in ambulatory electrocardiograms using a deep neural network. Nat. Med. *25*, 65–69. https://doi.org/10.1038/s41591-018-0268-3.
13. Castelvecchi, D. (2016). Can we open the black box of ai? Nature *538*, 20–23. https://doi.org/10.1038/538020a.
14. Du, M., Liu, N., and Hu, X. (2019). Techniques for interpretable machine learning. Commun. ACM *63*, 68–77. https://doi.org/10.1145/3359786.
15. Rudin, C. (2019). Stop explaining black box machine learning models for high stakes decisions and use interpretable models instead. Nat. Mach. Intell. *1*, 206–215. https://doi.org/10.1038/s42256-019-0048-x.
16. Teng, Q., Liu, Z., Song, Y., Han, K., and Lu, Y. (2022). A survey on the interpretability of deep learning in medical diagnosis. Multimed. Syst. *28*, 2335–2355. https://doi.org/10.1007/s00530-022-00960-4.
17. Mori, U., Mendiburu, A., Dasgupta, S., and Lozano, J.A. (2018). Early classification of time series by simultaneously optimizing the accuracy and earliness. IEEE Trans. Neural Netw. Learn. Syst. *29*, 4569–4578. https://doi.org/10.1109/TNNLS.2017.2764939.
18. Lv, J., Hu, X., Li, L., and Li, P. (2019). An effective confidence-based early classification of time series. IEEE Access *7*, 96113–96124. https://doi.org/10.1109/ACCESS.2019.2929644.
19. Xie, J., Shen, Z., and Zhang, C. (2020). Efficient projection-free online methods with stochastic recursive gradient. In Proceedings of the AAAI Conference on Artificial Intelligence (AAAI), pp. 6446–6453. https://doi.org/10.1609/aaai.v34i04.6116.
20. Lopez-Paz, D., and Ranzato, M. (2017). Gradient episodic memory for continual learning. In Proceedings of the Advances in Neural Information Processing Systems (NeurIPS), pp. 6467–6476.
21. Kirkpatrick, J., Pascanu, R., Rabinowitz, N., Veness, J., Desjardins, G., Rusu, A.A., Milan, K., Quan, J., Ramalho, T., Grabska-Barwinska, A., et al. (2017). Overcoming catastrophic forgetting in neural networks. Proc. Natl. Acad. Sci. USA *114*, 3521–3526. https://doi.org/10.1073/pnas.1611835114.
22. Rolnick, D., Ahuja, A., Schwarz, J., Lillicrap, T.P., and Wayne, G. (2019). Experience replay for continual learning. In Proceedings of the Advances in Neural Information Processing Systems (NeurIPS), pp. 348–358.
23. Kiyasseh, D., Zhu, T., and Clifton, D. (2021). A clinical deep learning framework for continually learning from cardiac signals across diseases, time, modalities, and institutions. Nat. Commun. *12*, 4221. https://doi.org/10.1038/s41467-021-24483-0.
24. Reyna, M.A., Josef, C., Seyedi, S., and Jeter, R. (2019). Early prediction of sepsis from clinical data: the physionet/computing in cardiology challenge 2019. In Computing in Cardiology, pp. 1–4. https://doi.org/10.23919/CinC49843.2019.9005736.
25. Yan, L., Zhang, H.T., Goncalves, J., Xiao, Y., Wang, M., Guo, Y., Sun, C., Tang, X., Jing, L., Zhang, M., et al. (2020). An interpretable mortality prediction model for covid-19 patients. Nat. Mach. Intell. *2*, 283–288. https://doi.org/10.1038/s42256-020-0180-7.
26. Johnson, A.E.W., Pollard, T.J., Shen, L., Lehman, L.W.H., Feng, M., Ghassemi, M., Moody, B., Szolovits, P., Celi, L.A., and Mark, R.G. (2016). Mimic-iii, a freely accessible critical care database. Sci. Data *3*, 160035. https://doi.org/10.1038/sdata.2016.35.
27. Menne, M., Williams, C., Jr., and Vose, R. (2016). Long-term daily and monthly climate records from stations across the contiguous United States (us historical climatology network)(ndp-019). In Tech. Rep., Environmental System Science Data Infrastructure for a Virtual Ecosystem. https://doi.org/10.3334/CDIAC/CLI.NDP019.
28. Dau, H.A., Bagnall, A., Kamgar, K., Yeh, C.C.M., Zhu, Y., Gharghabi, S., Ratanamahatana, C.A., and Keogh, E. (2019). The ucr time series archive. IEEE/CAA J. Autom. Sinica *6*, 1293–1305. https://doi.org/10.1109/JAS.2019.1911747.
29. Rubanova, Y., Chen, T.Q., and Duvenaud, D. (2019). Latent ordinary differential equations for irregularly-sampled time series. In Proceedings of the Advances in Neural Information Processing Systems (NeurIPS), pp. 5321–5331.
30. Hong, S., Zhou, Y., Shang, J., Xiao, C., and Sun, J. (2020). Opportunities and challenges of deep learning methods for electrocardiogram data: a systematic review. Comput. Biol. Med. *122*, 103801. https://doi.org/10.1016/j.compbiomed.2020.103801.
31. Sun, C., Hong, S., Song, M., and Li, H. (2021). Te-esn: time encoding echo state network for prediction based on irregularly sampled time series data. In Proceedings o the International Joint Conference on Artificial Intelligence (IJCAI), pp. 3010–3016. https://doi.org/10.24963/ijcai.2021/414.
32. Sun, C., Hong, S., Song, M., Li, H., and Wang, Z. (2021). Predicting covid-19 disease progression and patient outcomes based on temporal deep learning. BMC Med. Inform. Decis. Mak. *21*, 45. https://doi.org/10.1186/s12911-020-01359-9.
33. Parisi, G.I., Kemker, R., Part, J.L., Kanan, C., and Wermter, S. (2019). Continual lifelong learning with neural networks: a review. Neural Netw. *113*, 54–71. https://doi.org/10.1016/j.neunet.2019.01.012.
34. Gee, A.H., Chang, J., Ghosh, J., and Paydarfar, D. (2018). Bayesian online changepoint detection of physiological transitions. In International Conference of the IEEE Engineering in Medicine and Biology Society (EMBC), pp. 45–48. https://doi.org/10.1109/EMBC.2018.8512204.
35. Gallo Marin, B., Aghagoli, G., Lavine, K., Yang, L., Siff, E.J., Chiang, S.S., Salazar-Mather, T.P., Dumenco, L., Savaria, M.C., Aung, S.N., et al. (2021). Predictors of covid-19 severity: a literature review. Rev. Med. Virol. *31*, 1–10. https://doi.org/10.1002/rmv.2146.
36. Choi, E., Xu, Z., Li, Y., Dusenberry, M., Flores, G., Xue, E., and Dai, A.M. (2020). Learning the graphical structure of electronic health records with graph convolutional transformer. In Proceedings of the AAAI Conference on Artificial Intelligence (AAAI), pp. 606–613. https://doi.org/10.1609/aaai.v34i01.5400.
37. Ammon, C.J., Velasco, A.A., Lay, T., and Wallace, T.C. (2021). Chapter 8 - earthquake prediction, forecasting, & early warning. In Foundations of Modern Global Seismology, second edition (Academic Press), pp. 223–248. https://doi.org/10.1016/B978-0-12-815679-7.00015-X.
38. Lee, W.Y., Park, S.K., and Sung, H.H. (2021). The optimal rainfall thresholds and probabilistic rainfall conditions for a landslide early warning system for chuncheon, Republic of Korea. Landslides *18*, 1721–1739. https://doi.org/10.1007/s10346-020-01603-3.

CellPress
OPEN ACCESS

39. Wang, Y., Wang, L., Rastegar-Mojarad, M., Moon, S., Shen, F., Afzal, N., Liu, S., Zeng, Y., Mehrabi, S., Sohn, S., and Liu, H. (2018). Clinical information extraction applications: a literature review. J. Biomed. Inform. *77*, 34–49. https://doi.org/10.1016/j.jbi.2017.11.011.

40. Yu, G., Cheng, K., Liu, Q., Wu, W., Hong, H., and Lin, X. (2022). Clinical outcomes of severe sepsis and septic shock patients with left ventricular dysfunction undergoing continuous renal replacement therapy. Sci. Rep. *12*, 9360. https://doi.org/10.1038/s41598-022-13243-9.

41. Maslove, D.M., Tang, B., Shankar-Hari, M., Lawler, P.R., Angus, D.C., Baillie, J.K., Baron, R.M., Bauer, M., Buchman, T.G., Calfee, C.S., et al. (2022). Redefining critical illness. Nat. Med. *28*, 1141–1148. https://doi.org/10.1038/s41591-022-01843-x.

42. Danlos, F.X., Grajeda-Iglesias, C., Durand, S., Sauvat, A., Roumier, M., Cantin, D., Colomba, E., Rohmer, J., Pommeret, F., Baciarello, G., et al. (2021). Metabolomic analyses of covid-19 patients unravel stage-dependent and prognostic biomarkers. Cell Death Dis. *12*, 258. https://doi.org/10.1038/s41419-021-03540-y.

43. Mallya, A., and Lazebnik, S. (2018). Packnet: adding multiple tasks to a single network by iterative pruning. In Proceedings of the IEEE Conference on Computer Vision and Pattern Recognition (CVPR), pp. 7765–7773. https://doi.org/10.1109/CVPR.2018.00810.

44. Bai, B., Liang, J., Zhang, G., Li, H., Bai, K., and Wang, F. (2021). Why attentions may not be interpretable? In ACM SIGKDD Conference on Knowledge Discovery and Data Mining (SIGKDD), pp. 25–34. https://doi.org/10.1145/3447548.3467307.

45. Johnson, J.M., and Khoshgoftaar, T.M. (2019). Survey on deep learning with class imbalance. J. Big Data *6*, 27. https://doi.org/10.1186/s40537-019-0192-5.

Patterns, Volume *4*

Supplemental information

Continuous diagnosis and prognosis by controlling the update process of deep neural networks

Chenxi Sun, Hongyan Li, Moxian Song, Derun Cai, Baofeng Zhang, and Shenda Hong

Supplemental Information

1. Supplemental Related Work and Concepts

Time series is one of the most common data forms, the popularity of time series classification has attracted increasing attention in many practical fields, such as healthcare and industry. In the real world, the class of a time series is usually labeled at the final time. For example, patients' outcomes will come at the end. Most deep learning (DL) models are good at single-shot classification, classifying data at a fixed time after learning time series within a fixed period. Because DL methods assume that the observed data is independent and identically distributed (i.i.d) and subsequences in the same period maintain one distribution.

However, in the real world, more and more time-sensitive applications need to classify time series continuously before the final labeled time. For example, in the intensive care unit (ICU), diagnosis and prognosis are needed at any time to provide more opportunities for doctors to rescue lives. Each hour of delay has been associated with roughly a 4-8% increase in sepsis mortality. But patient labels, e.g. mortality or morbidity, are only available at the onset time but unknown in the early stages. In response to the current demand, we propose a new concept – Continuous Classification of Time Series (CCTS), to classify time series at every time point before the labeled time. For example, using vital signs like blood pressure to diagnose patients continuously.

1.1. Single-shot Classification

Single-shot classification methods classify at a fixed time. A time series $X = \{x_m\}_{m=1}^{M}$ is labeled with classes C. Single-shot classification aims to classify X at a time $t_m, m \leq M$ with the minimum loss $\mathcal{L}(f(X_{1:m}), C)$.

The foundation is the Classification of Time Series (CTS), making classification based on the full-length data. But in time-sensitive applications, Early Classification of Time Series (ECTS), classifying at an early time, is more critical. For example, early diagnosis helps for sepsis outcomes. Nowadays, Recurrent Neural Networks (RNNs) and Convolutional Neural Networks (CNNs) have shown good performances for CTS and ECTS by modeling long-term dependencies, addressing data irregularities, learning frequency features, etc.

Definition 1 (Classification of Time Series, CTS). *A dataset $\mathcal{D} = \{(X^n, C^n)\}_{n=1}^{N}$ has N time series. Each time series X is labeled with a class C, CTS classifies time series using the full-length data by model $f : X \rightarrow C$*

Definition 2 (Early Classification of Time Series, ECTS). *A dataset of time series $\mathcal{D} = \{(X^n, C^n)\}_{n=1}^{N}$ has N samples. Each time series $X = \{x_m\}_{m=1}^{M}$ is labeled with a class C. ECTS classifies time series in an advanced time t_m by model $f : \{X_{1:m}\} \rightarrow C$, where $m < M$.*

As shown in Figure s1, the existing classification tasks of time series are the single-shot classification, where the classification is performed only once at the final or an early time. However, many real-world applications require continuous classification. For example, intensive care patients should be detected and diagnosed at all times to facilitate timely life-saving. The above methods only classify once and just lean a single data distribution. They have good performances on i.i.d data at a fixed time, like early 6 hours sepsis diagnosis, but fail for multi-distribution. In fact, continuous classification is composed of multiple single-shot classifications.

1.2. Continuous Classification

Without the loss of generality, we use the univariate time series to present the problem. Multivariate time series can be described by changing x_m to x_m^d. d is the d-th dimension. Note that single-shot classification optimizes the objective with a single loss $\mathcal{L}(f(x), c)$. RU should consider the multi-distribution and classify more times.

Definition 3 (Continuous Classification, CC). *A dataset of time series $\mathcal{D} = \{(X^n, C^n)\}_{n=1}^{N}$ has N samples. Each time series $X = \{X_m\}_{m=1}^{M}$ is labeled with a class C. CC classifies time series at every advanced time $t_m, m = 1, ..., M$ by model $f : \{X_{1:m}\}_{m=1}^{M} \rightarrow C$.*

We summarize the four requirements of continuous diagnosis and prognosis in Introduction section into three technical requirements of Continuous Classification of Time Series (CCTS).

Table s1
Notations and Description

Notation	Description
$\mathcal{T}$, $\mathcal{T}^*$	Time series dataset
$\mathcal{D}$	Distribution set
$\mathcal{M}$	Task series
$\mathcal{L}$	Classification loss
$\mathcal{O}$	Objective function
X, C	A time series sample and its class
x_m, t_m	The m-th observed value and time of X
f	Classification model
θ	Model parameters
g	Gradient
α	Importance coefficient

Requirement 1: the ability to model multi-distributed data. In the real-world dataset, the label of a time series is usually determined at the final time. The DL model trained by this dataset can only give the single-shot classification at the onset time after observing the full-length time series. For CCTS, the model need to learn time series from different advanced stages so that it can classify at every time: When the data changes, the model performance needs to maintain, and accuracy cannot reduce.

Requirement 2: potential for earlier classification with guaranteed accuracy. Early classification is necessary for many time-sensitive applications. For example, early detection is critical for sepsis, where each hour of delayed treatment has been associated with roughly an 4-8% increase in mortality[1]. But basic questions about the limits of early detection remain unanswered. If one wants to pursue higher accuracy, the model would tend to predict late as it wants to observe more data.

Requirement 3: offline learning and sustainable use. In many time-sensitive and privacy-related scenarios, we need to directly use the well-trained model instead of updating it while using it. Because if the model is updated in real time, it will lead to the unsupervised learning mode caused by the lack of timely access to labels and finally increase the risk of misjudgment[2]. Further, in subsequent applications, when a batch of new data is obtained, we hope to continue to use the current model instead of designing a new model. Because the data which has the old knowledge may still occur, while the new system cannot handle the old knowledge well.

Most methods use multi-model to learn multi-distribution, like SR[3] and ECEC[4]. They divide data by time stages and design different classifiers for different distributions. But the operation of data division and classifier selection will cause additional losses.

Currently, some sub-disciplines also study the mode of continuous learning or continuous classification. But their setting does not match our needs and their methods can't address our issues, as shown in Figure s2.

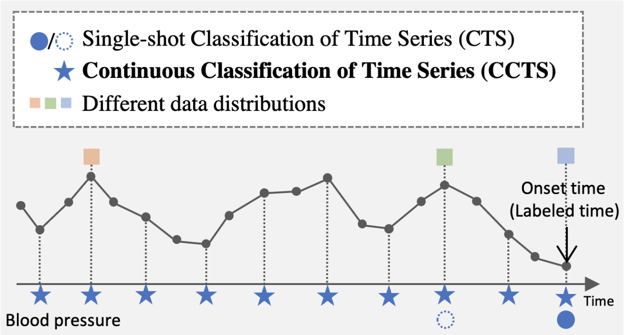

Figure s1: Continuous Classification of Time Series (CCTS)

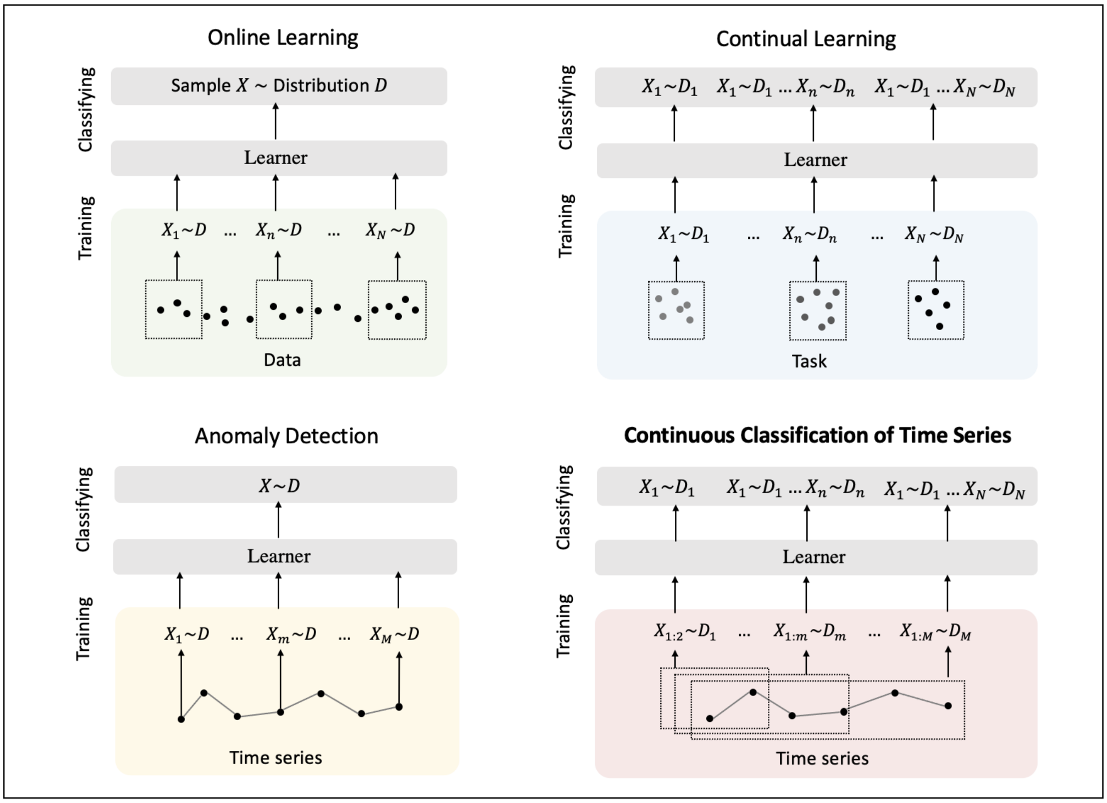

Figure s2: Differences and Similarities between CCTS and Other Concepts

Definition 4 (Online Learning, OL). *A OL issue has a sequence of dataset* $\mathcal{T} = \{\mathcal{T}^n\}_{n=1}^N$ *for one task* $\mathcal{M}$. *All datasets has the same distribution* D. *The goal is to find the optimal solution of* $\mathcal{M}$ *after* N *iterations by minimize the regret* $\mathcal{R} := \sum_{n=1}^N (f^n(\mathcal{T}^n) - \min f^n(\mathcal{T}^n))$.

Online Learning (OL) models the incoming data steam continuously to solve an overall optimization problem with the partially observed data. It focuses more on issues in data steam, rather than the dynamics of time series. Most importantly, it maintains only one data distribution, rather than learning multiple. Thus, OL cannot meet the Requirement 1 and Requirement 2.

Definition 5 (Continual Learning, CL). *A CL issue* $\mathcal{M} = \{\mathcal{M}^m\}_{m=1}^M$ *has a sequence of* M *tasks. Each task* $\mathcal{M}^m$ *has a dataset* $\mathcal{T}^m = \{X^n, C^n\}_{n=1}^N$ *with* N *samples and corresponding class labels. CL learns a new task at every moment. The goal is to control the statistical risk of all seen tasks* $\sum_{m=1}^M \mathbb{E}_{(X,C)\in\mathcal{T}^m}[\mathcal{L}(f((X;\theta), C)]$ *with loss* $\mathcal{L}$, *network function* f *and parameters* θ.

Continual Learning (CL) enables the model to learn new tasks over time without forgetting the old tasks. It learns a new task at every new moment and each new task corresponds to a new data distribution. Replay-based methods re-train the model by the old data to consolidate memory[5]; Regularization-based methods restrain parameter update of neural networks to limit forgetting[6]; Model-based methods change network structure or apply multiple models to response to different tasks[7]. But most of the above methods have the problems of storage limitation, distributions drifts and model overfitting. Most importantly, in CL, the definition of old and new tasks is clear and the division of distribution is fixed. But in CCTS, the distributions, that is, the tasks in CL, is not determined and need to be defined. While the dynamic time series has data correlation over time, which easily further causes the overfitting problem. Thus, CL cannot meet the partial Requirement 1 and the Requirement 2.

Definition 6 (Anomaly Detection, AD). *A sequence $X = \{X_n\}_{n=1}^{N}$ has N observations and maintains one data distribution D. AD task aims to find the abnormal observation X_a in X by evaluating whether X_a deviates from D.*

Anomaly Detection (AD) identifies data that does not conform to the expected pattern. It mainly maintains one data distribution and gives an alarm when an exception occurs. Thus, AD cannot meet Requirement 1 and partial Requirement 2.

Because the existing research can not meet the current demand, we propose a new concept CCTS.

2. Supplemental Mathematics

Assumption 1. *The compact convex set $C \subseteq \mathbb{R}^d$ has diameter D. $\forall \theta_1, \theta_2 \in C$,*

$$||\theta_1 - \theta_2|| \leq D \tag{1}$$

Assumption 2. *The stochastic gradient $\nabla F^m(\theta, \xi^m)$ is unbiased with $\mathbb{E}_{\xi^m}[\nabla F^m(\theta, \xi^m)] = \nabla f^m(\theta)$ and is L-Lipschitz continuous over the constraint set C with*

$$||\nabla F^m(\theta_1, \xi^m) = \nabla F^m(\theta_{2,\xi^m})|| \leq L||\theta_1 = \theta_2||, \forall \theta_1, \theta_2 \in C. \tag{2}$$

The above Assumption immediately implies that f^m is differentiable and has L-Lipschitz-continuous gradients.

In the stochastic online setting, we denote the expected loss function as the $\overline{f} = \mathbb{E}_{f^m \sim \mathcal{D}[f^m]}$. In order to obtain high probability results. We assume the following:

Assumption 3. *The distance between the stochastic gradient $\nabla F^m(\theta, \xi^m)$ and the exact gradient is bounded over the constraint set C, for any $theta \in C, t \in \{1, ..., T\}$, there exist $\sigma < \infty$ such that with probability 1,*

$$||\nabla F^m(\theta, \xi^m) - \nabla \overline{f}(\theta)||^2 \leq \sigma^2 \tag{3}$$

The difference of $f^m(\theta)$ and $\overline{f}^m(\theta)$ is bounded over the constraint set C. $\forall \theta \in C, t \in \{1, ..., T\}$, there exist $M^2 < \infty$ such that with probability 1,

$$|f(\theta) - \overline{f}(\theta)| \leq M^2 \tag{4}$$

We show that the norm of the gradient estimation error $\varepsilon^m := d^m - \nabla \overline{f}(\theta^m)$ converges to zero rapidly w.h.p.

First, we reformulate ε^m as the sum of a martingale difference sequence $\{\epsilon_{m,k}\}_{k=1}^{m}$ w.r.t. a filtration $\{\mathcal{F}^m\}_{k=0}^{m}$, i.e., $\varepsilon^m = \sum_{k=1}^{m} \epsilon_{m,k}$, where $\mathbb{E}[\epsilon_{m,k}|\mathcal{F}_{m-1}|] = 0$ and $\mathcal{F}_{m-1}$ is the θ-filed generate by $\{f_i, \xi_i\}_{i=1}^{k-1}$. By showing that $||\epsilon_{m,k}|| \leq c_{m,k}$ for some constant $c_{m,k}$, one can relate the Hoeffding-type concentration inequality. With carefully chosen $\{\rho^m\}_{m=1}^{M}$ and $\{\eta^m\}_{m=1}^{M}$, the quantity q^m can be shown to converge to 0 at a sublinear rate by induction. As a result, $||\varepsilon^m||$ converges to zero at a sublinear rate w.h.p. as stated in the following lemma. D is diameter of convex set, L is L-Lipschitz-continuous.

Lemma 1. *With $\rho^m = \eta^m = \frac{1}{(1+m)^a}$ for some $a \in (,1]$, if Assumptions are satisfied for any $t \geq 1$ and $\delta_0 \in (0,1)$ we have w.p.at least $1 - \delta_0$,*

$$||\varepsilon^m|| \leq 2(2LD) + \frac{3^a \sigma}{3^a - 1}(m+1)^{-\frac{a}{2}} \sqrt{2 \log(\frac{4}{\delta_0})}. \tag{5}$$

Lemma 1 shows that the gradient approximation error $||\varepsilon^m||$ converges to zero at a fast sublinear rate $\tilde{\mathcal{O}}(\frac{1}{m^{\frac{a}{2}}})$ w.h.p if $\rho^m = \eta^m = \frac{1}{(1+m)^a}$ for any $a \in (0,1]$. This result is critical to the regret analysis of our methods.

Theorem 1. *With $\rho^m = \eta^m = \frac{1}{1+m}$. If $\overline{f}$ is convex and Assumption are satisfied, then w.p. at least $1-\delta$ for any $\delta \in (0,1)$ for any $\delta \in (0,1)$,*

$$\begin{aligned}\mathcal{R}^m \leq &(logM+1)(f(\theta^1)-f(\theta^*)) \\ &+(16LD^2+16\sigma+4B)\sqrt{2Mlog\frac{8M}{\delta}} \\ &+\frac{1}{2}LD^2(logM+1)^2\end{aligned} \tag{6}$$

3. Supplemental Experimental Procedures

3.1. Datasets

- SEPSIS dataset[8] has 30,336 records with 2,359 diagnosed sepsis. Early diagnose is critical to improve sepsis outcome[1]. In this dataset, the time series are the changes of 40 related patient features, the label at each time is sepsis or non-sepsis. Early diagnose can improve sepsis outcome.
- COVID-19 dataset[9] has 6,877 blood samples of 485 COVID-19 patients from Tongji Hospital, Wuhan, China. Mortality prediction helps for treatment and rational resource allocation[10]. In this dataset, the time series are the changes of blood samples, the label at each time is mortality or survival. Mortality prediction helps for personalized treatment and rational resource allocation
- MIMIC-III dataset[11] has 19,993 admission records of 7,537 patients. We focus on 8 diagnoses (ICD-9): Diabetes(249), Hypertension (401), Heart Failure (428), Pneumonia (480-486), Gastric Ulcer (531), Hepatopathy (571), Nephropathy (580-589), SIRS (995.9). The time series are vital signs, and labels at each time are some diagnoses.
- USHCN dataset[12] has the daily meteorological data of 48 states in U.S. from 1887 to 2014. We focus on 4 weather conditions in New York: sunny, overcast, rainfall, snowfall. The time series are records of 4 neighboring states, labels at each time are weather after a weak. Rainfall warning is not only the demand of daily life, but also can help prevent natural disasters.
- UCR time series classification archive[13] consists of 128 time series datasets. We have selected 15 datasets covering multiple data types (spectro, sensor, image, motion, simulated) and classification tasks (binary-, three-, four-, five-, six-classification).
- UCR-EQ dataset has 471 earthquake records from UCR time series database archive. It is the univariate time series of seismic feature value. Natural disaster early warning, like earthquake warning, helps to reduce casualties and property losses.
- ACTIV dataset[14] consists of 3D positions collected from five individuals performing various activities including walking, sitting, lying, standing, etc. Each sample is a multivariate time series from 4 sensors and we focus on 11 types of activities.

3.2. Baselines

- ECTS-based methods:
  - LSTM trains a model by learning time series at every time stage.
  - SR[3] gives the fusion result of multiple models trained by the full-length data
  - ECEC[4] has trains a set of classifiers by data in different time stages.
- CL-based methods:
  - EWC[15] is a regularization-based method, training a model to remember the old tasks by constraining important parameters to stay close to their old values.

  - GEM[6] is a regularization-based method, updating parameters by finding gradients which are at acute angles to old gradients
  - CLEAR[16] is a replay-based method, using the reservoir sampling to limit the number of stored samples to a fixed budget.
  - CLOPS[5] is a replay-based method, re-learning old tasks wshen forgetting appears.
- OL-based methods:
  - OSFW[17] uses stochastic gradient estimator
  - ORGFW[18] uses recursive gradient estimator.

References

1. Seymour, C.W., Gesten, F., Prescott, H.C. (2017). Time to treatment and mortality during mandated emergency care for sepsis. New England Journal of Medicine 376, 2235–2244. 10.1016/j.jemermed.2017.08.088.
2. Si, N., Zhang, F., Zhou, Z., Blanchet, J. (2020). Distributionally robust policy evaluation and learning in offline contextual bandits. In: Proceedings of the International Conference on Machine Learning (ICML), pp. 8884–8894.
3. Mori, U., Mendiburu, A., Dasgupta, S., Lozano, J.A. (2018). Early classification of time series by simultaneously optimizing the accuracy and earliness. IEEE Transactions on Neural Networks and Learning Systems 29, 4569–4578. 10.1109/TNNLS.2017.2764939.
4. Lv, J., Hu, X., Li, L., Li, P. (2019). An effective confidence-based early classification of time series. IEEE Access 7, 96113–96124. 10.1109/ACCESS.2019.2929644.
5. Kiyasseh, D., Zhu, T., Clifton, D. (2021). A clinical deep learning framework for continually learning from cardiac signals across diseases, time, modalities, and institutions. Nature Communications 12, 4221. 10.1038/s41467-021-24483-0.
6. Lopez-Paz, D., Ranzato, M. (2017). Gradient episodic memory for continual learning. In: Proceedings of the Advances in Neural Information Processing Systems (NeurIPS), pp. 6467–6476.
7. Mallya, A., Lazebnik, S. (June 2018). Packnet: Adding multiple tasks to a single network by iterative pruning. In: Proceedings of the IEEE Conference on Computer Vision and Pattern Recognition (CVPR), pp. 7765–7773. 10.1109/CVPR.2018.00810.
8. Reyna, M.A., Josef, C., Seyedi, S., Jeter, R. (2019). Early prediction of sepsis from clinical data: the physionet/computing in cardiology challenge 2019. In: Computing in Cardiology, pp. 1–4. 10.23919/CinC49843.2019.9005736.
9. Yan, L., Zhang, H.T., Goncalves, J., Xiao, Y., Wang, M., Guo, Y., Sun, C., Tang, X., Jing, L., Zhang, M., et al. (2020). An interpretable mortality prediction model for covid-19 patients. Nature Machine Intelligence 2, 283–288. 10.1038/s42256-020-0180-7.
10. Sun, C., Hong, S., Song, M., Li, H., Wang, Z. (2020). Predicting covid-19 disease progression and patient outcomes based on temporal deep learning. BMC Medical Informatics and Decision Making 21, 45. 10.1186/s12911-020-01359-9.
11. Johnson, A.E., Pollard, T.J., Shen, L., Li-wei, H.L., Feng, M., Ghassemi, M. (2016). Mimic-iii, a freely accessible critical care database. Scientific Data 3, 160035. 10.1038/sdata.2016.35.
12. Menne, M., Williams Jr, C., Vose, R.. Long-term daily and monthly climate records from stations across the contiguous united states (us historical climatology network)(ndp-019). Tech. Rep., Environmental System Science Data Infrastructure for a Virtual Ecosystem (2016). 10.3334/CDIAC/CLI.NDP019.
13. Dau, H.A., Bagnall, A., Kamgar, K., Yeh, C.C.M., Zhu, Y., Gharghabi, S., Ratanamahatana, C.A., Keogh, E. (2019). The ucr time series archive. IEEE/CAA Journal of Automatica Sinica 6, 1293–1305. 10.1109/JAS.2019.1911747.
14. Rubanova, Y., Chen, T.Q., Duvenaud, D. (2019). Latent ordinary differential equations for irregularly-sampled time series. In: Proceedings of the Advances in Neural Information Processing Systems (NeurIPS), pp. 5321–5331.
15. Kirkpatrick, J., Pascanu, R., Rabinowitz, N.C., Veness, J., Desjardins, G., Rusu, A.A. (2017). Overcoming catastrophic forgetting in neural networks. Proceedings of the National Academy of Sciences 114, 3521–3526. 10.1073/pnas.1611835114.
16. Rolnick, D., Ahuja, A., Schwarz, J., Lillicrap, T.P., Wayne, G. (2019). Experience replay for continual learning. In: Proceedings of the Advances in Neural Information Processing Systems (NeurIPS), pp. 348–358.
17. Chen, L., Harshaw, C., Hassani, H., Karbasi, A. (2018). Projection-free online optimization with stochastic gradient: From convexity to submodularity. In: Proceedings of International Conference on Machine Learning (ICML), pp. 814–823.
18. Xie, J., Shen, Z., Zhang, C. (2020). Efficient projection-free online methods with stochastic recursive gradient. In: Proceedings of the AAAI Conference on Artificial Intelligence (AAAI), pp. 6446–6453. 10.1609/aaai.v34i04.6116.

Table s2
Classification Accuracy (AUC-ROC↑) at the First 5 Time Steps
*k% means the current classification time is k% of the total time of the full-length time series; Bold font indicates the highest accuracy.
Our method is significantly better than baselines by using Bonferroni-Dunn test. RU can classify more accurately at every time. The average accuracy is about 2% higher, especially in the early time, being 5% higher for 10%-length data. Take sepsis diagnosis as an example, compared with the best baseline, our method improves the accuracy by 1.4% on average, 2.2% in the early 50% time stage when the key features are unobvious. Each hour of delayed treatment increases sepsis mortality by 4-8%. With the same accuracy, we can predict 0.972 h in advance.

Dataset	Method	10%	20%	30%	40%	50%
SEPSIS	LSTM	0.576±0.063	0.629±0.035	0.735±0.064	0.736±0.064	0.745±0.056
	SR	0.626±0.035	0.659±0.015	0.768±0.013	0.791±0.026	0.803±0.018
	ECEC	0.623±0.024	0.669±0.019	0.761±0.016	0.793±0.016	0.811±0.015
	EWC	0.671±0.027	0.733±0.023	0.799±0.015	0.827±0.036	0.832±0.028
	GEM	0.670±0.026	0.730±0.024	0.802±0.018	0.826±0.033	0.834±0.026
	CLEAR	0.680±0.028	0.732±0.024	0.801±0.015	0.825±0.035	0.833±0.025
	CLOPS	0.684±0.025	0.733±0.025	0.802±0.017	0.824±0.036	0.830±0.023
	RU	**0.690±0.032**	**0.734±0.038**	**0.812±0.022**	**0.828±0.036**	**0.835±0.024**
COVID-19	LSTM	0.605±0.044	0.701±0.033	0.793±0.022	0.833±0.015	0.844±0.013
	SR	0.636±0.014	0.730±0.024	0.810±0.013	0.867±0.016	0.901±0.013
	ECEC	0.639±0.013	0.732±0.028	0.829±0.013	0.870±0.016	0.901±0.026
	EWC	0.703±0.022	0.769±0.015	0.870±0.014	0.888±0.028	0.915±0.017
	GEM	0.699±0.025	0.779±0.017	0.871±0.015	0.885±0.022	0.914±0.019
	CLEAR	0.710±0.013	0.785±0.019	0.870±0.016	0.879±0.016	0.916±0.024
	CLOPS	0.709±0.017	0.775±0.013	0.869±0.012	0.900±0.017	0.918±0.026
	RU	**0.712±0.021**	**0.790±0.023**	**0.872±0.013**	**0.901±0.022**	**0.919±0.016**
MIMIC-III	LSTM	0.600±0.042	0.651±0.030	0.683±0.024	0.701±0.019	0.764±0.013
	SR	0.656±0.012	0.682±0.019	0.720±0.013	0.750±0.012	0.791±0.012
	ECEC	0.650±0.011	0.679±0.021	0.715±0.014	0.748±0.013	0.783±0.011
	EWC	0.652±0.009	0.674±0.018	0.716±0.012	0.747±0.014	0.785±0.012
	GEM	0.653±0.012	0.675±0.014	0.714±0.012	0.745±0.012	0.786±0.010
	CLEAR	0.658±0.011	0.682±0.009	0.722±0.009	0.751±0.013	0.793±0.010
	CLOPS	0.659±0.010	0.681±0.010	0.724±0.010	0.753±0.012	0.794±0.008
	RU	**0.662±0.012**	**0.689±0.013**	**0.759±0.011**	**0.758±0.012**	**0.801±0.010**
UCR-EQ	LSTM	0.695±0.044	0.711±0.038	0.803±0.024	0.843±0.019	0.854±0.017
	SR	0.700±0.015	0.736±0.014	0.830±0.016	0.863±0.015	0.871±0.024
	ECEC	0.703±0.013	0.738±0.018	0.828±0.017	0.865±0.014	0.873±0.026
	EWC	0.724±0.015	0.768±0.018	0.848±0.014	0.874±0.016	0.883±0.025
	GEM	0.723±0.014	0.767±0.017	0.850±0.015	0.876±0.016	0.890±0.024
	CLEAR	0.729±0.015	0.770±0.015	0.852±0.019	0.880±0.013	0.899±0.026
	CLOPS	0.728±0.016	0.773±0.016	0.855±0.015	0.878±0.016	0.896±0.028
	RU	**0.730±0.022**	**0.774±0.023**	**0.856±0.015**	**0.882±0.022**	**0.900±.0017**
USHCN	LSTM	0.682±0.014	0.700±0.028	0.721±0.013	0.745±0.028	0.784±0.023
	SR	0.702±0.014	0.730±0.022	0.745±0.016	0.761±0.023	0.809±0.024
	ECEC	0.707±0.017	0.736±0.024	0.748±0.015	0.760±0.025	0.806±0.025
	EWC	0.727±0.018	0.736±0.025	0.768±0.017	0.798±0.024	0.805±0.022
	GEM	0.720±0.019	0.728±0.026	0.772±0.015	0.781±0.023	0.801±0.026
	CLEAR	0.728±0.016	0.738±0.025	0.773±0.018	0.784±0.024	0.802±0.027
	CLOPS	0.728±0.012	0.740±0.024	0.769±0.019	0.781±0.025	0.800±0.024
	RU	**0.730±0.018**	**0.742±0.017**	**0.775±0.016**	**0.791±0.021**	**0.810±.0133**
ACTIV	LSTM	0.701±0.022	0.720±0.019	0.743±0.020	0.751±0.021	0.766±0.017
	SR	0.718±0.020	0.723±0.014	0.739±0.016	0.753±0.015	0.768±0.016
	ECEC	0.720±0.010	0.722±0.012	0.742±0.012	0.752±0.010	0.769±0.010
	EWC	0.721±0.012	0.726±0.013	0.744±0.012	0.754±0.009	0.770±0.008
	GEM	0.721±0.011	0.727±0.010	0.745±0.015	0.757±0.007	0.771±0.006
	CLEAR	0.726±0.012	0.730±0.013	0.744±0.014	0.758±0.011	0.775±0.011
	CLOPS	0.724±0.012	0.728±0.007	0.747±0.014	0.760±0.013	0.776±0.010
	RU	**0.729±0.010**	**0.732±0.013**	**0.752±0.014**	**0.763±0.011**	**0.780±0.010**

Table s3
Classification Accuracy (AUC-ROC↑) at the Last 5 Time Steps
*k% means the current classification time is k% of the total time of the full-length time series; Bold font indicates the highest accuracy.
Our method is significantly better than baselines by using Bonferroni-Dunn test. RU can classify more accurately at every time. The average accuracy is about 2% higher, especially in the early time, being 5% higher for 10%-length data. Take sepsis diagnosis as an example, compared with the best baseline, our method improves the accuracy by 1.4% on average, 2.2% in the early 50% time stage when the key features are unobvious. Each hour of delayed treatment increases sepsis mortality by 4-8%. With the same accuracy, we can predict 0.972 h in advance.

Dataset	Method	60%	70%	80%	90%	100%
SEPSIS	LSTM	0.748±0.043	0.773±0.032	0.795±0.027	0.813±0.025	0.827±0.039
	SR	0.827±0.037	0.835±0.013	0.845±0.014	0.859±0.022	0.866±0.023
	ECEC	0.815±0.014	0.827±0.016	0.849±0.016	0.859±0.017	0.863±0.014
	EWC	0.838±0.024	0.842±0.030	0.848±0.017	0.850±0.014	0.854±0.016
	GEM	0.836±0.028	0.841±0.034	0.849±0.014	0.851±0.016	0.853±0.012
	CLEAR	0.839±0.028	0.842±0.031	0.847±0.010	0.850±0.019	0.848±0.016
	CLOPS	0.838±0.026	0.842±0.030	0.850±0.017	0.853±0.010	0.857±0.018
	RU	**0.842±0.034**	**0.852±0.023**	**0.857±0.012**	**0.866±0.014**	**0.872±0.012**
COVID-19	LSTM	0.888±0.013	0.918±0.033	0.925±0.014	0.939±0.005	0.944±0.015
	SR	0.900±0.018	0.935±0.010	0.946±0.006	0.952±0.017	0.962±0.005
	ECEC	0.904±0.014	0.937±0.008	0.948±0.015	0.952±0.008	0.963±0.017
	EWC	0.923±0.014	0.935±0.007	0.940±0.013	0.950±0.013	0.954±0.008
	GEM	0.924±0.018	0.936±0.009	0.939±0.010	0.949±0.017	0.953±0.005
	CLEAR	0.926±0.014	0.933±0.011	0.941±0.007	0.948±0.009	0.952±0.008
	CLOPS	0.925±0.015	0.935±0.013	0.940±0.007	0.947±0.006	0.954±0.006
	RU	**0.927±0.006**	**0.955±0.008**	**0.960±0.011**	**0.963±0.009**	**0.967±0.008**
MIMIC-III	LSTM	0.780±0.021	0.791±0.024	0.825±0.020	0.820±0.020	0.849±0.021
	SR	0.792±0.017	0.795±0.016	0.805±0.017	0.821±0.015	0.856±0.013
	ECEC	0.792±0.013	0.791±0.012	0.810±0.012	0.820±0.013	0.861±0.012
	EWC	0.793±0.013	0.795±0.013	0.812±0.010	0.822±0.012	0.860±0.013
	GEM	0.791±0.013	0.794±0.013	0.813±0.012	0.820±0.013	0.859±0.012
	CLEAR	0.797±0.012	0.801±0.012	0.814±0.011	0.823±0.011	0.861±0.013
	CLOPS	0.799±0.010	0.803±0.011	0.813±0.012	0.821±0.009	0.860±0.010
	RU	**0.809±0.010**	**0.806±0.012**	**0.820±0.007**	**0.824±0.010**	**0.863±0.009**
UCR-EQ	LSTM	0.874±0.012	0.913±0.034	0.909±0.014	0.919±0.008	0.924±0.012
	SR	0.888±0.017	0.924±0.010	0.928±0.105	0.936±0.103	0.941±0.104
	ECEC	0.890±0.015	0.923±0.013	0.929±0.107	0.936±0.006	0.940±0.009
	EWC	0.895±0.014	0.910±0.017	0.923±0.102	0.930±0.005	0.933±0.003
	GEM	0.900±0.015	0.920±0.015	0.929±0.008	0.935±0.003	0.934±0.004
	CLEAR	0.904±0.012	0.918±0.019	0.923±0.004	0.928±0.007	0.932±0.005
	CLOPS	0.902±0.015	0.915±0.010	0.917±0.006	0.921±0.009	0.925±0.005
	RU	**0.906±0.005**	**0.928±0.007**	**0.933±0.010**	**0.940±0.005**	**0.946±0.003**
USHCN	LSTM	0.820±0.015	0.837±0.024	0.852±0.014	0.869±0.025	0.891±0.002
	SR	0.836±0.016	0.886±0.023	0.902±0.013	0.921±0.026	0.933±0.009
	ECEC	0.837±0.016	0.887±0.027	0.906±0.017	0.920±0.028	0.931±0.009
	EWC	0.834±0.016	0.867±0.026	0.896±0.017	0.906±0.020	0.926±0.007
	GEM	0.838±0.013	0.868±0.029	0.899±0.010	0.910±0.021	0.928±0.005
	CLEAR	0.837±0.010	0.867±0.023	0.879±0.012	0.899±0.027	0.921±0.004
	CLOPS	0.835±0.016	0.861±0.024	0.877±0.011	0.895±0.016	0.919±0.013
	RU	**0.841±0.012**	**0.898±0.022**	**0.910±0.015**	**0.928±0.013**	**0.939±0.013**
ACTIV	LSTM	0.790±0.021	0.822±0.021	0.835±0.022	0.866±0.020	0.879±0.020
	SR	0.821±0.011	0.834±0.013	0.846±0.009	0.870±0.012	0.877±0.012
	ECEC	0.822±0.010	0.830±0.010	0.842±0.010	0.872±0.011	**0.886±0.009**
	EWC	0.821±0.011	0.832±0.014	0.844±0.011	0.874±0.010	0.877±0.011
	GEM	**0.825±0.007**	0.831±0.010	0.842±0.012	0.873±0.010	0.878±0.008
	CLEAR	0.820±0.010	0.829±0.006	0.843±0.008	0.870±0.009	**0.886±0.006**
	CLOPS	**0.825±0.009**	0.833±0.006	0.844±0.009	**0.875±0.006**	0.885±0.006
	RU	**0.825±0.010**	**0.834±0.009**	**0.846±0.007**	0.874±0.008	0.885±0.010

Table s4
Average Classification Accuracy↑ (Rank↓) of Methods on UCR Time Series Classification Archive

	Class	LSTM	SR	ECEC	EWC	GRM	CLEAR	CLOPS	RU
Coffee	2	0.82(8)	0.83(6)	0.83(6)	0.85(4)	0.85(4)	0.89(2)	0.88(3)	0.91(1)
Gun Point	2	0.88(8)	0.89(7)	0.91(5)	0.90(6)	0.93(1)	0.93(1)	0.93(1)	0.93(1)
MoteStrain	2	0.79(8)	0.82(6)	0.81(7)	0.83(1)	0.83(1)	0.82(4)	0.82(3)	0.82(3)
SonyAIBOrobotsurface1	2	0.80(8)	0.82(4)	0.82(4)	0.82(4)	0.82(4)	0.85(1)	0.84(3)	0.85(1)
SonyAIBOrobotsurface2	2	0.78(6)	0.78(6)	0.78(6)	0.81(1)	0.79(5)	0.80(2)	0.80(2)	0.80(2)
Wafer	2	0.94(3)	0.94(3)	0.93(8)	0.95(1)	0.95(1)	0.94(3)	0.94(3)	0.94(3)
Lightning2	2	0.68(8)	0.69(7)	0.71(5)	0.70(6)	0.73(1)	0.73(1)	0.72(3)	0.72(3)
Yoga	2	0.78(6)	0.78(6)	0.78(6)	0.80(1)	0.79(3)	0.79(3)	0.79(3)	0.80(1)
CBF	3	0.73(8)	0.74(7)	0.75(3)	0.77(1)	0.76(2)	0.75(3)	0.74(6)	0.75(3)
ChlorineConcentration	3	0.70(8)	0.71(6)	0.72(4)	0.72(4)	0.71(6)	0.73(1)	0.73(1)	0.73(1)
FaceFour	4	0.75(2)	0.75(2)	0.75(2)	0.74(7)	0.74(7)	0.76(1)	0.75(2)	0.75(2)
Oliveoil	4	0.90(6)	0.89(8)	0.90(6)	0.91(5)	0.92(3)	0.92(3)	0.93(1)	0.93(1)
Beef	5	0.69(4)	0.69(4)	0.68(8)	0.69(4)	0.70(2)	0.70(2)	0.71(1)	0.69(4)
Symbols	6	0.70(6)	0.69(8)	0.70(6)	0.71(1)	0.71(1)	0.71(1)	0.71(1)	0.71(1)
SyntheticControl	6	0.84(2)	0.84(2)	0.84(2)	0.84(2)	0.83(8)	0.84(2)	0.84(2)	0.84(1)
Average Rank		6.1	5.5	5.2	3.2	3.3	2.0	2.3	**1.9**

Table s5
Continual Learning Performance (BWT↑) of Methods
[1]LSTM, SR and ECEC are not listed as they have no CL strategy. It's pointless to use BWT and FBT to evaluate them.
Our strategy can alleviate the catastrophic forgetting and promote the overall performance by sub-distribution. RU has the best performance on the early time series, showing the ability of LM to alleviate catastrophic forgetting. RU has the highest BWT, meaning it has the lowest negative influence that learning the new tasks has on the old tasks. RU has the highest FWT, meaning it has the highest positive influence that learning the former data distributions has on the task, especially for Sepsis and COVID-19 datasets.

Dataset \ Method[1]	OSFW	ORGFW	GEM	CLOPS	RU
SEPSIS	–0.070	–0.066	+0.017	+0.006	**+0.032**
COVID-19	–0.026	–0.015	+0.012	+0.004	**+0.021**
MIMIC-III	–0.153	–0.161	+0.104	+0.043	**+0.125**
UCR-EQ	+0.109	+0.112	+0.123	+0.149	**+0.162**
USHCN	+0.065	+0.072	+0.098	+0.082	**+0.124**
ACTIV	–0.128	–0.107	+0.048	+0.039	**+0.075**
UCR	+0.072	+0.86	+0.101	+0.109	**+0.114**

Table s6
Continual Learning Performance (FWT↑) of Baselines
[1]LSTM, SR and ECEC are not listed as they have no CL strategy. It's pointless to use BWT and FBT to evaluate them.

Dataset \ Method	OSFW	ORGFW	GEM	CLOPS	RU
SEPSIS	+0.323	+0.309	+0.265	+0.237	**+0.415**
COVID-19	+0.469	+0.478	+0.421	+0.289	**+0.498**
MIMIC-III	+0.197	+0.217	+0.287	+0.246	**+0.364**
UCR-EQ	+0.312	+0.363	+0.343	+0.384	**+0.399**
USHCN	+0.300	+0.316	+0.322	+0.301	**+0.348**
ACTIV	+0.134	+0.167	+0.184	+0.192	**+0.201**
UCR	+0.210	+0.226	+0.240	+0.253	**+0.259**

Table s7

AUC-ROC↑, BWT↑, FWT↑ and Gradient Fluctuation R↓ of Ablation of RU

Both LM and PM strategies contribute to model performance. If we remove two strategies respectively, the model accuracy will decline, the relation between tasks will become worse, the model instability will increase.

Dataset	Method[1]	Time 4	Time 6	Time 8	Time 10	BWT	FWT	R
SEPSIS	w/o PM	0.750±.03	0.796±.02	0.812±.02	0.830±.02	-0.102	+0.165	0.401
	w/o LM	0.743±.03	0.790±.02	0.801±.01	0.825±.02	-0.111	+0.160	0.400
	RU	**0.828±.01**	**0.842±.01**	**0.857±.01**	**0.872±.01**	**+0.032**	**+0.415**	**0.247**
COVID-19	w/o PM	0.879±.02	0.924±.02	0.931±.01	0.948±.01	-0.058	+0.195	0.328
	w/o LM	0.870±.01	0.914±.01	0.925±.00	0.935±.01	-0.088	+0.190	0.306
	RU	**0.901±.00**	**0.927±.01**	**0.960±.00**	**0.967±.00**	**+0.021**	**+0.498**	**0.248**
MIMIC-III	w/o PM	0.746±.01	0.760±.01	0.805±.01	0.828±.01	+0.053	+0.272	0.344
	w/o LM	0.755±.01	0.770±.01	0.812±.01	0.829±.01	+0.103	+0.312	0.338
	RU	**0.758±.01**	**0.806±.00**	**0.820±.01**	**0.863±.00**	**+0.125**	**+0.364**	**0.333**
UCR-EQ	w/o PM	0.776±.01	0.812±.02	0.838±.00	0.885±.01	+0.053	+0.246	0.277
	w/o LM	0.775±.01	0.810±.02	0.840±.00	0.886±.01	+0.080	+0.338	0.249
	RU	**0.882±.01**	**0.906±.00**	**0.933±.01**	**0.946±.00**	**+0.162**	**+0.399**	**0.246**
USHCN	w/o PM	0.706±.01	0.808±.02	0.849±.00	0.905±.01	+0.053	+0.289	0.209
	w/o LM	0.771±.01	0.812±.02	0.850±.00	0.910±.01	+0.080	+0.294	0.212
	RU	**0.791±.01**	**0.841±.00**	**0.910±.01**	**0.939±.00**	**+0.124**	**+0.348**	**0.257**
ACTIV	w/o PM	0.738±.01	0.808±.02	0.810±.01	0.827±.01	+0.033	+0.120	0.350
	w/o LM	0.723±.02	0.812±.01	0.826±.01	0.875±.01	+0.032	+0.123	0.347
	RU	**0.763±.01**	**0.825±.01**	**0.846±.01**	**0.885±.01**	**+0.075**	**+0.261**	**0.323**
UCR	w/o PM	0.752	0.786	0.799	0.845	+0.104	+0.231	0.212
	w/o LM	0.753	0.793	0.810	0.844	+0.103	+0.220	0.204
	RU	**0.790**	**0.815**	**0.824**	**0.859**	**+0.114**	**+0.259**	**0.153**

Table s8

Performance (AUC-ROC↑, BWT↑, FWT↑) of RU under Different Class Number and Training Orders of MIMIC-III Dataset

Class number and training order will influence the result: Fewer classes lead to better performance of RU; A sound training order can improve the model performance. If we increase the diagnosis number in MIMIC-III, the accuracy will decrease. Besides, No matter what order is adopted, RU has stable accuracy. It shows the possibility of global optimization potential of PM in RU. Based on the Gaussian distribution $\mathcal{N}(\mu, \sigma^2)$ of each class and the similarity of class i and class j $S(i,j) = 1 - \sqrt{1 - \sqrt{\frac{2\sigma_i\sigma_j}{\sigma i^2 + \sigma j^2}} e^{-\frac{1}{4}\frac{(\mu_i-\mu_j)^2}{\sigma i^2+\sigma j^2}}}$, we can obtain a new class order in Figure s6.

	**2 Classes**	4 Classes	6 Classes	8 Classes	Random	ICD-9	Similarity
AUC-ROC	**0.859±.01**	0.831±.01	0.816±.01	0.797±.01	0.832±.01	0.830±.01	**0.845±.01**
BWT	**+0.153**	+0.142	+0.139	+0.135	+0.125	+0.122	**+0.133**
FWT	**+0.398**	+0.384	+0.379	+0.365	+0.364	+0.358	**+0.367**

Table s9

Performance (AUC-ROC↑, BWT↑, FWT↑) of RU under Different Training Order in Different Time Stages of MIMIC-III Dataset

Order	30%	60%	90%	BWT	FWT
Random	0.757±.01	0.788±.01	0.832±.01	+0.125	+0.364
ICD-9	0.759±.01	0.783±.01	0.830±.01	+0.122	+0.358
Similarity	**0.762±.01**	**0.796±.00**	**0.845±.01**	**+0.133**	**+0.367**

Table s10
COVID-19 Classification Accuracy with Non-uniform Training Sets and Validation Sets
↓ means the accuracy is greatly reduced. Bold font indicates the smallest decline in accuracy.

Subset	LSTM	SR	ECEC	EWC
Male	0.955±0.013	0.968±0.014	0.969±0.016	0.965±0.012
Female	0.924±0.013	0.945±0.004	0.947±0.015	0.939±0.018
Age 55-	0.954±0.013	0.965±0.014	0.967±0.015	0.967±0.013
Age 55+	0.923±0.014	0.941±0.007	0.943±0.018	0.931±0.008↓
Test	0.950±0.011	0.964±0.013	0.968±0.015	0.966±0.012
Valid.	0.944±0.014	0.962±0.006	0.963±0.014	0.954±0.003

Subset	GEM	CLEAR	CLOPS	RU
Male	0.965±0.004	0.978±0.009	0.978±0.014	0.971±0.010
Female	0.938±0.003	0.919±0.008↓	0.921±0.009↓	**0.947±0.002**
Age 55-	0.964±0.009	0.977±0.008	0.979±0.012	0.972±0.010
Age 55+	0.923±0.040↓	0.902±0.006↓	0.914±0.007↓	**0.945±0.006**
Test	0.962±0.006	0.979±0.009	0.978±0.010	0.970±0.007
Valid.	0.953±0.005	0.952±0.009↓	0.954±0.004↓	**0.967±0.006**

Table s11
Classification Accuracy (AUC-ROC↑) of RU under Training Sets with Different Data Size
k% means the volume of sub dataset is k% of the corresponding original dataset; Bold font indicates the highest accuracy; *means that the accuracy of RU is higher 2% than this method.

Dataset	Method	20%	40%	60%	80%	100%
SEPSIS	LSTM	0.658*	0.669*	0.691*	0.733*	0.747
	SR	0.682	0.700	0.725*	0.759*	0.768
	ECEC	0.679*	0.702	0.719*	0.755*	0.770
	EWC	0.685	0.708	0.729*	0.768*	0.772*
	GEM	0.693	0.704	0.740*	0.771*	0.781*
	CLEAR	0.687	0.705	0.741	0.776	0.789
	CLOPS	0.698	0.710	0.745	0.779	0.783*
	RU	**0.701**	**0.712**	**0.760**	**0.794**	**0.803**
COVID-19	LSTM	0.713*	0.730*	0.765*	0.819*	0.834*
	SR	0.751*	0.767*	0.806	0.822*	0.842*
	ECEC	0.755*	0.770*	0.796*	0.829*	0.856*
	EWC	0.763	0.785	0.794*	0.835*	0.849*
	GEM	0.769	0.772*	0.793*	0.849	0.856*
	CLEAR	0.776	0.791	0.810	0.856	0.866*
	CLOPS	0.775	0.789	0.809	0.848	0.874
	RU	**0.781**	**0.800**	**0.821**	**0.863**	**0.888**
UCR-EQ	LSTM	0.724*	0.765*	0.804*	0.809*	0.813*
	SR	0.758*	0.784*	0.828*	0.813*	0.831*
	ECEC	0.790	0.770*	0.815*	0.827*	0.838*
	EWC	0.785	0.791*	0.833*	0.855*	0.862*
	GEM	0.780	0.775*	0.840*	0.857*	0.863*
	CLEAR	0.784	0.808	0.859	0.864*	0.870*
	CLOPS	0.792	0.809	0.864	0.871	0.875*
	RU	**0.797**	**0.817**	**0.872**	**0.886**	**0.896**
USHCN	LSTM	0.701*	0.730*	0.732*	0.760*	0.763*
	SR	0.731*	0.769*	0.782*	0.801*	0.805*
	ECEC	0.747*	0.774	0.800*	0.807*	0.816*
	EWC	0.739*	0.768*	0.810	0.817	0.826*
	GEM	0.737*	0.772	0.809	0.811*	0.818*
	CLEAR	0.757	0.780	0.812	0.819	0.823*
	CLOPS	0.775	0.785	0.817	0.825	0.839
	RU	**0.776**	**0.790**	**0.821**	**0.835**	**0.843**
ACTIV	LSTM	0.732*	0.780	0.809	0.814	0.825
	SR	0.743*	0.783	0.814	0.815	0.823
	ECEC	0.745*	0.786	0.817	0.818	0.827
	EWC	0.742*	0.785	0.816	0.819	0.828
	GEM	0.748*	0.782	0.813	0.817	0.829
	CLEAR	0.752	0.787	0.816	0.823	0.832
	CLOPS	0.757	0.789	0.819	0.820	0.830
	RU	**0.770**	**0.795**	**0.820**	**0.830**	**0.834**
UCR	LSTM	0.663*	0.714*	0.744*	0.796*	0.813
	SR	0.672*	0.717*	0.754*	0.806	0.820
	ECEC	0.670*	0.721	0.755*	0.810	0.819
	EWC	0.676*	0.722	0.763*	0.814	0.821
	GEM	0.675*	0.721	0.765*	0.812	0.820
	CLEAR	0.681*	0.721*	0.770	0.813	0.822
	CLOPS	0.687	0.725	0.771	0.815	0.820
	RU	**0.705**	**0.741**	**0.788**	**0.819**	**0.824**

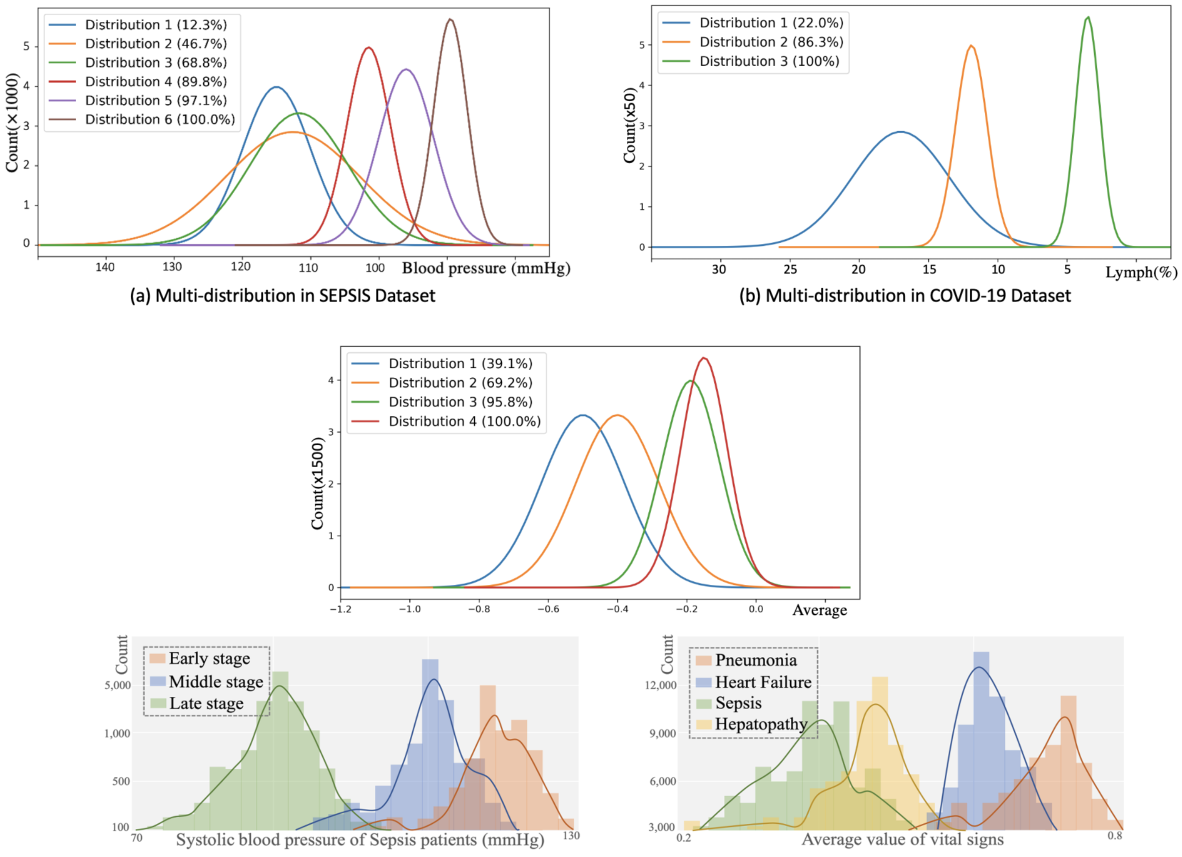

(a) Multi-distribution in SEPSIS Dataset

(b) Multi-distribution in COVID-19 Dataset

(c) Multi-distribution in MIMIC-III Dataset

Figure s3: Multi-distribution in Medical Datasets

Before discussing the method performance, we show the basic scenario of CCTS– multiple distributions. The data in different time stages have distinct statistical characteristics and finally form multi-distribution. The fundamental goal of the following experiment is to model them.

Sub-figure (a) shows six distributions in SEPSIS Dataset, each of them belongs to a time stage. Corresponding to the four stages in Figure 3, distribution 1, 2, 3 belong to stage 1, distribution 4 belongs to stage 2, distribution 5 belongs to stage 3, and distribution 6 belongs to stage 4.

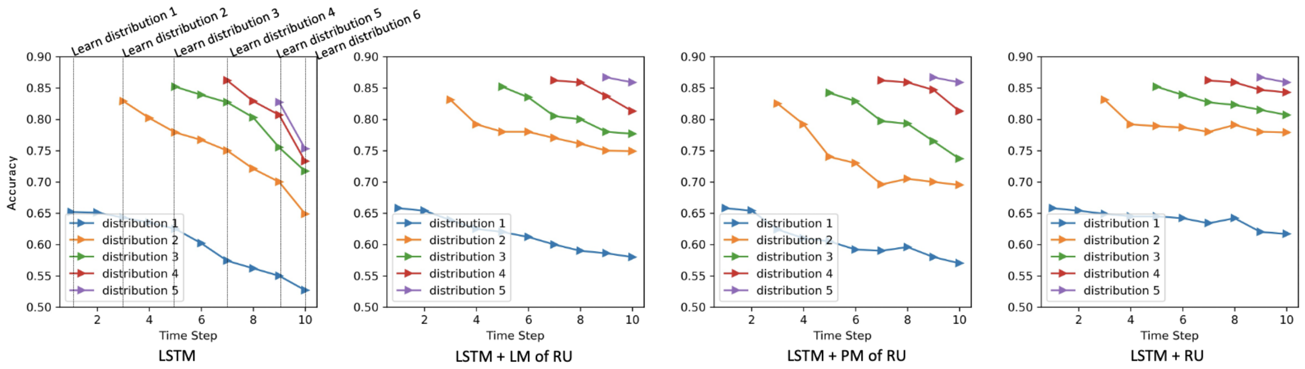

(a) The Accuracy Change when Learning SEPSIS Dataset

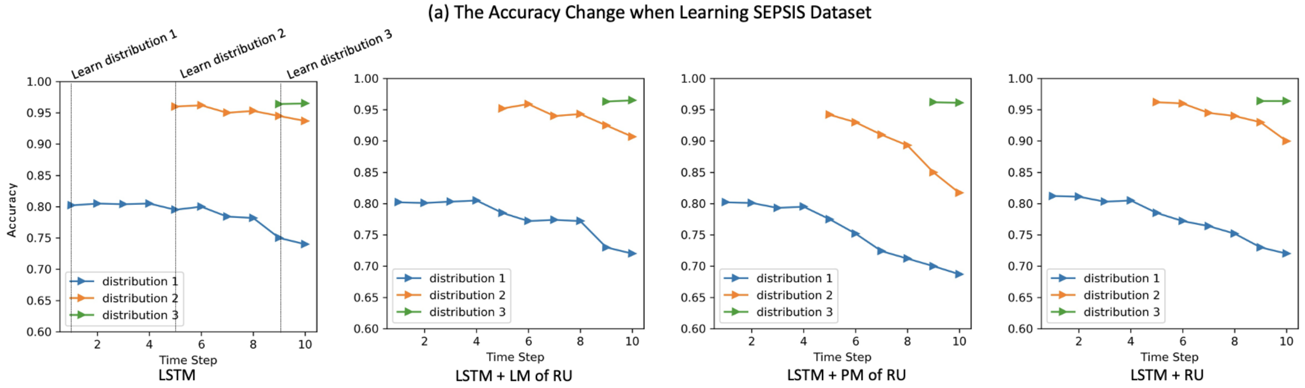

(b) The Accuracy Change when Learning COVID-19 Dataset

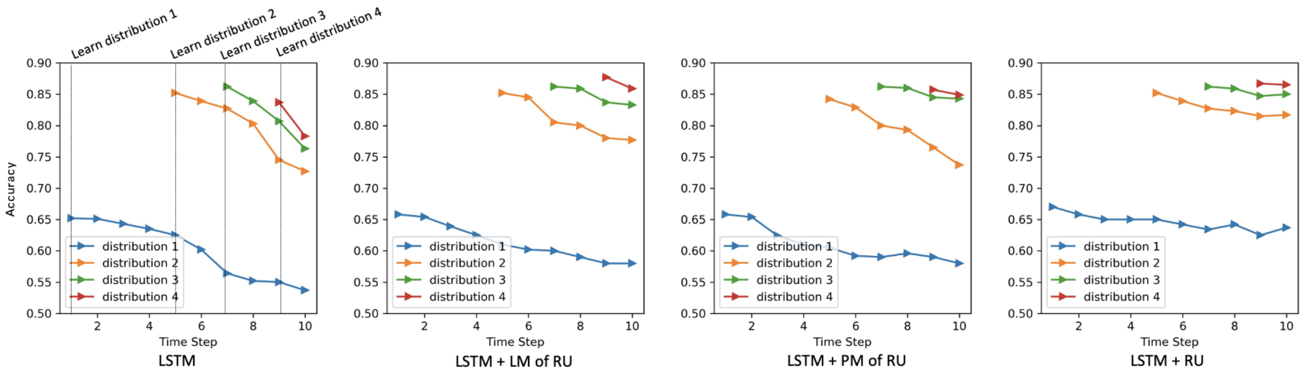

(c) The Accuracy Change when Learning MIMIC-III Dataset

Figure s4: The Accuracy Change in Continuous Classification
(1) When the model learns a new distribution, its performance on old distributions becomes worse (catastrophic forgetting); (2) After using the Limitation Mechanism (LM) of our method (RU), when learning a new distribution, the performance of the model on old distributions will not decline much; (3) After using the Promotion Mechanism (PM) of our method (RU), in some cases, e.g., at time step 8, learning the new distribution will help the model perform on the old distribution; (4) After using our method (RU, PM+LM), the accuracy of the model can be basically maintained in continuous learning

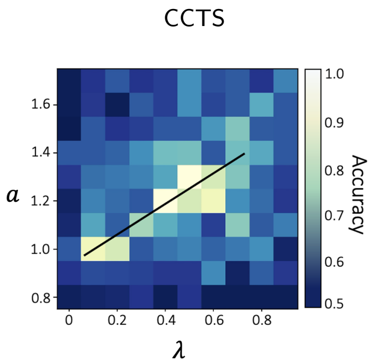

Figure s5: Parameter Test

Parameter λ decides the constraint degree on parameter update in Equation 5 of LM. In practice, we optimize it using the search method supplied by mature tools; Parameter a decides ρ in PM by $\rho_m = \eta_m = \frac{1}{(m+1)^a}$. ρ determines the correlation between current and previous gradients in Equation 12. We can optimize it using the search method supplied by mature tools.

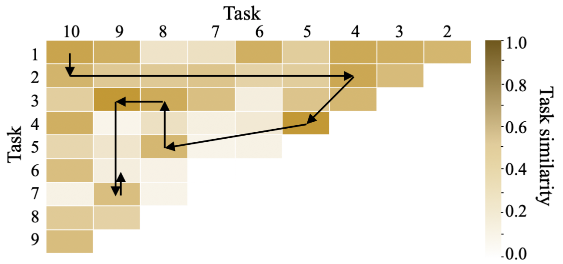

Figure s6: Task Similarity

In MIMIC-III dataset, the diagnoses with ICD-9 order are 1:HIV, 2:Brain Cancer, 3:Diabetes, 4:Hypertension, 5:Heart Failure, 6:Pneumonia, 7:Gastric Ulcer, 8:Hepatopathy, 9:Nephropathy, 10:SIRS. As we focus on eight diseases (3-10). The new similarity order is 10, 2, 4, 5, 8, 9, 7, 6.

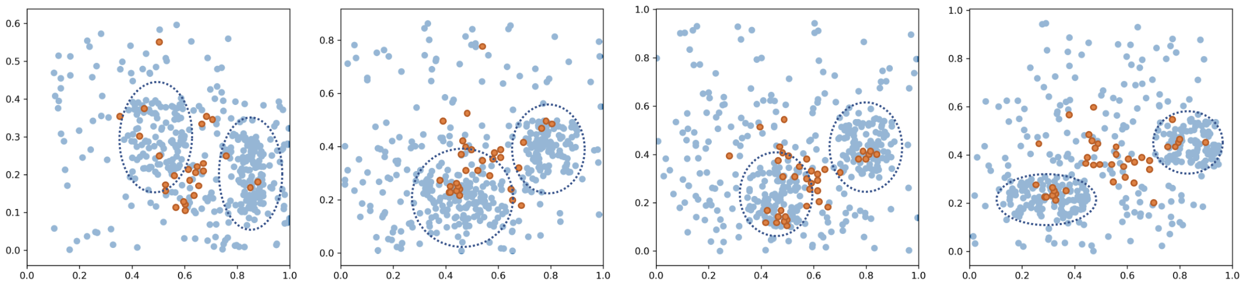

Figure s7: The Important Samples in Four SEPSIS Distribution Buffers when Using the Replay-based Method [5]

The four sub-figures are the sample representations of distribution 2,3,4,5 in Figure s3(a). Important samples should be in the circle, but the important samples found by this method are not completely correct.

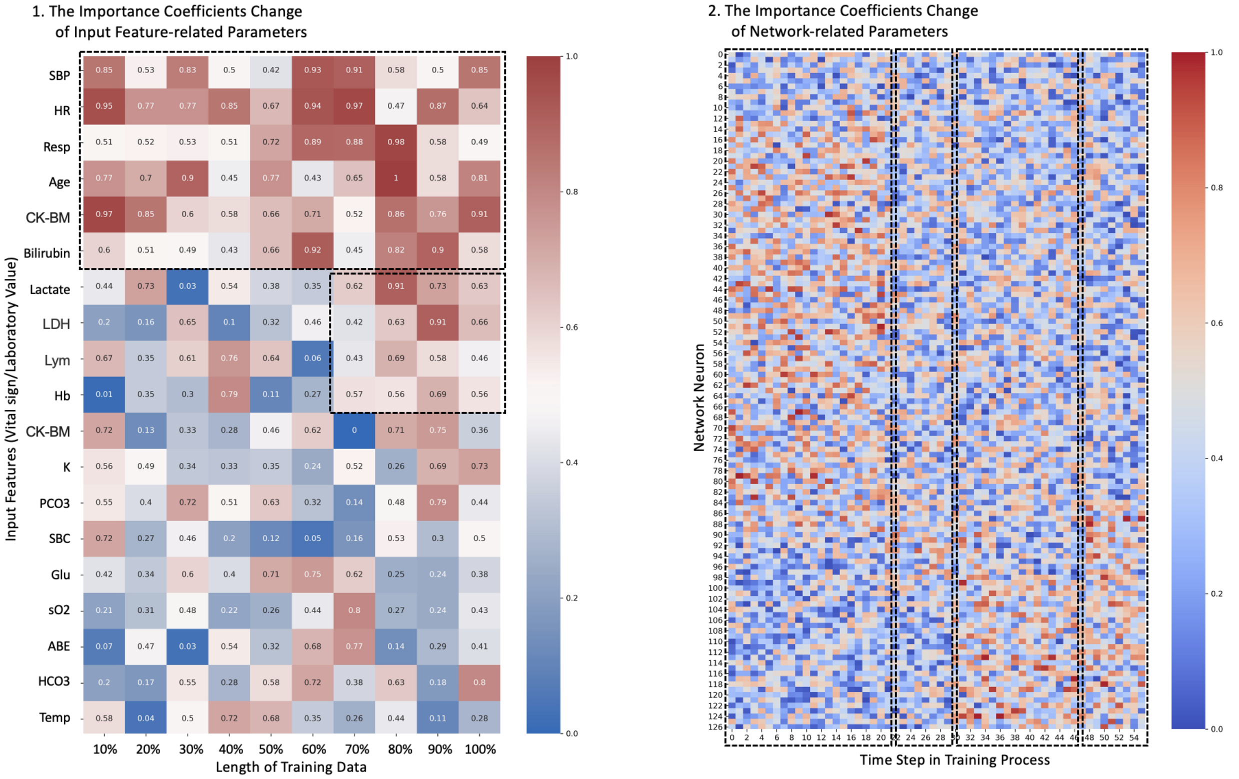

Figure s8: The Importance Change of Input Features and Network Parameters for MIMIC-III Eight Diseases Classification Subfigure 1 shows the important input features for eight diseases classification: SBP, HR, Resp, Age, CK-BM, Bilirubin, Lactate, LDH, Lym, Hb, etc. Among them, SBP, HR, Resp, Age, CK-BM, and Bilirubin are important in the whole process; Lactate, LDH, Lym, and Hb are gradually important in the middle and late stages. This shows the necessity of continuous vital sign monitoring and blood routine examination during hospitalization, as well as the importance of detailed laboratory examination in later stages. Meanwhile, these important features are more related to circulatory system diseases, kidney disease, and liver disease, which also shows that the extracted MIMIC-III dataset has more records about these diseases. For example, 9,012 of the 19,993 records are about circulatory system disease/heart disease.
Subfigure 2 shows the change of importance coefficients of network-related parameters. It forms four stages in eight diseases classification. Under the constraint of `RU`, there is a big difference between the last three stages and the first stage. It implies that the patient's state has changed significantly since the second stage.
Since this task is not specific to a single disease, we can only call them task stages and important features, not disease stages and biomarkers.
